# Melon *short internode* (*CmSi*) encodes an ERECTA-like receptor kinase regulating stem elongation through auxin signaling

**DOI:** 10.1038/s41438-020-00426-6

**Published:** 2020-12-01

**Authors:** Sen Yang, Kaige Zhang, Huayu Zhu, Xiaojing Zhang, Wenkai Yan, Nana Xu, Dongming Liu, Jianbin Hu, Yufeng Wu, Yiqun Weng, Luming Yang

**Affiliations:** 1 College of Horticulture, Henan Agricultural University, 63 Nongye Road, 450002 Zhengzhou, China; 2 State Key Laboratory of Crop Genetics and Germplasm Enhancement, Bioinformatics Center, Nanjing Agricultural University, 210095 Nanjing, China; 3 USDA-ARS, Vegetable Crops Research Unit, Horticulture Department, University of Wisconsin–Madison, Madison, WI 53706 USA

**Keywords:** Non-model organisms, Plant morphogenesis

## Abstract

Plant height is one of the most important agronomic traits that directly determines plant architecture, and compact or dwarf plants can allow for increased planting density and land utilization as well as increased lodging resistance and economic yield. At least four dwarf/semidwarf genes have been identified in different melon varieties, but none of them have been cloned, and little is known about the molecular mechanisms underlying internode elongation in melon. Here, we report map-based cloning and functional characterization of the first semidwarf gene *short internode* (*Cmsi*) in melon, which encodes an ERECTA-like receptor kinase regulating internode elongation. Spatial-temporal expression analyses revealed that *CmSI* exhibited high expression in the vascular bundle of the main stem during internode elongation. The expression level of *CmSI* was positively correlated with stem length in the different melon varieties examined. Ectopic expression of *CmSI* in *Arabidopsis* and cucumber suggested CmSI as a positive regulator of internode elongation in both species. Phytohormone quantitation and transcriptome analysis showed that the auxin content and the expression levels of a number of genes involved in the auxin signaling pathway were altered in the semidwarf mutant, including several well-known auxin transporters, such as members of the ABCB family and PIN-FORMED genes. A melon polar auxin transport protein CmPIN2 was identified by protein–protein interaction assay as physically interacting with CmSI to modulate auxin signaling. Thus, CmSI functions in an auxin-dependent regulatory pathway to control internode elongation in melon. Our findings revealed that the ERECTA family gene *CmSI* regulates stem elongation in melon through auxin signaling, which can directly affect polar auxin transport.

## Introduction

The deployment of dwarf or semidwarf genes was one of the main driving forces of the Green Revolution starting in the 1960s^1–3^. The reduction in plant height can increase lodging resistance, the harvest index and yield and more efficiently utilize resources^1,3^. Several phytohormones, such as auxins, gibberellic acids (GAs), brassinosteroids (BRs), and strigolactones (SLs), play important roles in regulating plant height^4–8^. GAs are well known to play significant roles in regulating stem elongation, leaf differentiation and seed germination. Most GA-insensitive and GA-deficient mutants are characterized by short internodes with rough and dark green leaves^2,4,9–11^. BRs and SLs are also extensively involved in regulating plant height^12,13^. A number of dwarfism genes involved in BR and SL biosynthesis and signal pathways have been characterized in various plant species^8,14,15^.

Auxin is a shoot-to-root phytohormone, and its roles in regulating several important agricultural traits, such as plant height and shoot branching, have also been well established^5,16^. The TRYPTOPHAN AMINOTRANSFERASE OF ARABIDOPSIS (TAA) and YUCCA (YUC) flavin monooxygenase-like enzymes control auxin biosynthesis in two independent pathways^17,18^. Polar auxin transport, mediated by the efflux-facilitating PIN-FORMED (PIN) family members, the influx carrier AUX1 protein family, and a number of the p-glyco-protein ABC transporters (also called ABCB family), plays an essential role in plant height regulation by building up the auxin maxima and gradient^19–22^. PIDs, encoding serine/threonine protein kinases belonging to the AGCVIIIa kinase family, have been reported to catalyze the efflux of auxin from cells through apical-basal PIN polar localization and/or phosphorylation of PIN proteins^23–25^. Overexpression of *ZmPIN1a* in maize resulted in reduced internode length, plant height, and ear height by increasing IAA transport from shoots to roots^26^. Overexpression of *PIN2* and *PIN5a* in rice enhanced auxin transport from shoots to roots, resulting in shorter plant height and larger tiller angle^27,28^.

The *Arabidopsis* ERECTA family receptor kinase genes *ERECTA* (*ER*), *ERECTA-LIKE1* (*ERL1*), and *ERECTA-LIKE2* (*ERL2*) all encode leucine-rich repeat receptor-like kinases, which have been shown to regulate stem elongation. The *erecta* mutant exhibits reduced stem and hypocotyl size, and the triple mutant *ererl1erl2* is extremely dwarfed^29^. The mechanisms of ER-family genes in regulating the development of leaf serrations and stomata have been intensively studied^30–32^. The EPIDERMAL PATTERNING FACTOR (EPF)/EPIDERMAL PATTERNING FACTOR LIKE (EPFL) family signaling peptides can physically interact with ER family receptor kinases to affect leaf teeth and stomatal development^30–32^. However, how stem elongation is regulated at the molecular level by ER-family genes remains unclear. Previous studies have shown that overexpression of the *Arabidopsis* auxin synthesis gene *YUCCA5* can rescue the dwarf phenotype of the *ERECTA* mutant *er-103* (ref. ^33^). Increasing endogenous or exogenous auxin levels could also partially rescue short hypocotyl defects in the *ererl1erl2* triple mutant^34^. However, how the genetic interaction between the ERECTA family and auxin signaling control stem and hypocotyl development is still poorly understood.

Melon (*Cucumis melo* L. 2*n* = 2*x* = 24), a member of the Cucurbitaceae family, is an economically important vegetable crop worldwide^35,36^. More than 32 million tons of melon was produced in 2017, and China was the largest melon producer and consumer, accounting for more than half of the total production (FAO; http://faostat.fao.org/). In China, melon is produced in either open fields (where it exhibits a creeping habit) or in protected environments (with trellis support). In recent years, the area of protected melon cultivation has been constantly increasing, accounting for 65.87% of the total cultivated areas in 2017. The semidwarf plant architecture (reduced internode length and few lateral branches) has great advantages for protected melon cultivation, with its increase in plant density and hence yield per unit land area and reduced labor cost due to less pruning. To date, four recessively inherited dwarf/semidwarf mutants, *si-1*, *si-2*, *si-3*, and *mdw1*, have been reported in melon^35,37^. However, only *mdw1* was loosely mapped on chromosome 7 by comparative mapping with cucumber^35^. None of these genes have been cloned, and little is known about the molecular mechanisms of plant height regulation in melon.

Here, we report the identification, map-based cloning, and functional characterization of a novel *short internode* semidwarf mutant in melon. We show that *CmSI* encodes an ERECTA-like receptor kinase and is mainly expressed in the vascular bundle of melon stems during internode elongation, and its overexpression in cucumber and *Arabidopsis* can promote stem elongation. We demonstrated that CmSI physically interacted with the polar auxin transport gene CmPIN2, which established the link between ERECTA family genes and auxin signaling in regulating stem elongation.

## Results

### Morphological characterization of short internode mutants in melon

**Fig. 1:**
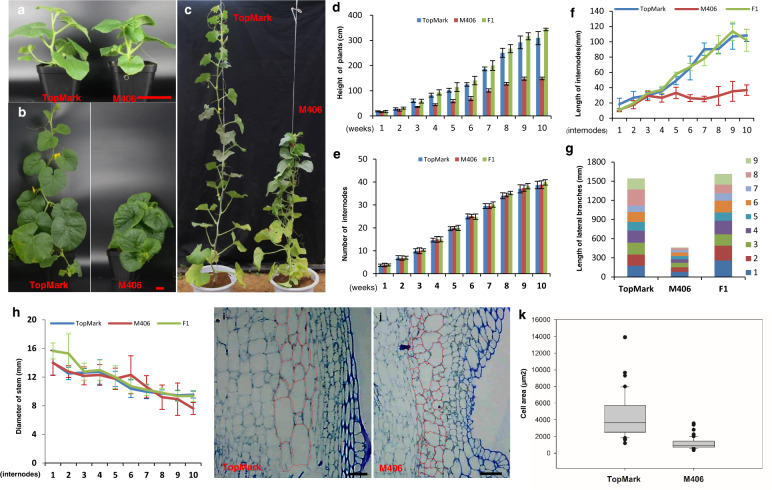
Morphological characterization and statistical analysis of two parental lines, TopMark and M406. **a**–**c** Morphological characterization of two melon lines at different stages (1, 3, and 6 weeks after transplanting). **d**, **e** Plant height and stem internode numbers of two melon lines at different stages (1–10 weeks) after transplanting. The internode length (**f**), lateral branch length (**g**), and stem diameter (**h**) for the first ten internodes of two parental lines at 45 days after transplanting. **i**, **j** Cytological characterization of the melon normal line TopMark (**i**) and semidwarf line M406 (**j**). **k** Quantification of the cell size of the eighth internodes in three different TopMark and M406 plants. The cell size in the M406 plants was significantly smaller than that in TopMark plants. The data are presented as the mean ± SD. Scale bars: 10 cm (**a**, **b**), 100 μm (**i**, **j**)

Compared with the wild-type (WT) inbred line TopMark, the stem length was significantly decreased in the semidwarf mutant M406. The plant height of M406 could be easily distinguished after the fifth true leaf stage (Fig. 1a–c). At full maturity, the stem length of the mutant plants was approximately half that of the WT plants (Fig. 1d). All F_1_ plants from the cross between TopMark and M406 showed normal plant height (Fig. 1d), suggesting the recessive nature of the mutation. There was no significant difference in the total number of internodes between the two parental lines. Therefore, the semidwarf phenotype of M406 was due to the reduced internode length (Fig. 1e, f). Furthermore, the length of lateral branches at each node was significantly longer in the WT than in the mutant, but a difference in the diameter of the main stem and lateral branches was not observed between the two parental lines (Fig. 1g, h).

Considering that the short internode may be caused by a decrease in cell number or a reduction in cell size, we examined the microscopic structure of the internodes for TopMark and M406. The cell structures of thin-sectioned eighth internodes of 40-day-old plants were examined under a light microscope (Fig. 1i, j). We found that the cells, including the parenchyma cells in particular, in the internodes of M406 were significantly smaller than those of TopMark (Fig. 1k), suggesting that the shorter internodes of the main stems of M406 were due to reduced cell size.

### Fine mapping of the Cmsi locus

**Fig. 2:**
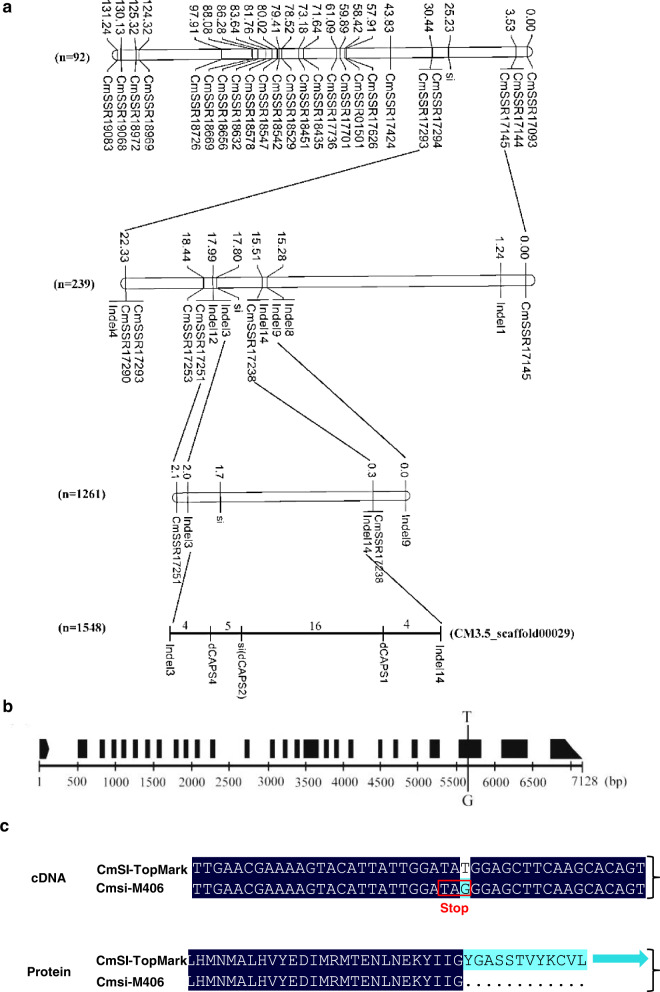
Map-based cloning of the *Cmsi* gene. **a** Fine mapping of the *Cmsi* gene. *Cmsi* was delimited in a 110 kb region between markers dCAPS4 and dCAPS1. Marker dCAPS2 cosegregates with the semidwarf phenotype in F_2_ plants. **b** Genomic structure of the candidate gene *MELO3C016916*, which contains 27 exons and is predicted to encode an LRR receptor-like serine/threonine protein kinase ERECTA. **c** The single base substitution in the 1995th CDS of *CmSI* results in premature termination of the protein in the mutant

Among 1261 TopMark × M406 F_2_ plants, 330 exhibited a semidwarf phenotype, and 931 had a WT phenotype, which was consistent with the 3 (WT):1 (semidwarf) segregation ratio (Table S1), indicating that the semidwarf mutation was controlled by a single recessive gene in melon, which was named *Cmsi*. Initial linkage analysis with SSR markers in 92 F_2_ plants indicated that the *Cmsi* locus was located on the short arm of melon chromosome (Chr) 7 (LGVII). The two flanking markers were CmSSR17145 and CmSSR17293, which were 21.70 and 5.21 cM to the *Cmsi* locus, respectively. According to their physical positions on the DHL92 draft genome assembly, CmSSR17145 and CmSSR17293 were both located in the same scaffold, CM3.5_scaffold00029, with a physical distance of 1.08 Mb. Among 30 additional SSR markers from this region, four were polymorphic between M406 and TopMark. The six markers were employed to genotype 239 F_2_ plants. The resulting genetic map is shown in Fig. 2a. The *Cmsi* locus was flanked by CsSSR17251 and CsSSR17238, which were physically 127.20 kb.

To identify new markers in the candidate region, TopMark and M406 were resequenced with Illumina HiSeq2500. After trimming low quality and short reads, 32,479,967 (9.73 Gb) and 32,479,967 (10.53 Gb) clean reads were obtained. Read alignment against the DHL92 sequence in the target region identified 14 polymorphic indels (>4 bp difference) between the two parental lines, 7 of which were successfully mapped with 239 F_2_ plants (Fig. 2a). To further confirm the mapping result, three indels and two SSR markers closely linked to *Cmsi* were selected for genotyping in an extended large F_2_ mapping population with 1,261 plants, and *Cmsi* was mapped between Indel 3 and Indel 14, which were 1.7 cM away from each other. The two Indel markers were used to genotype 1548 F_2_ plants, and 44 recombinants were identified. Four new SNP-derived dCAPS markers were developed in the candidate region between Indel 3 and Indel 14 and used to genotype these recombinants. The *Cmsi* locus was finally mapped in a 110 kb region defined by dCAPS1 and dCAPS4 (from 30,394,615 to 30,501,700 on CM3.5_scaffold00029) (Fig. 2a).

In the DHL92 reference genome, 14 genes were predicted in the 110 kb region (Table S2). From the resequencing data, only two SNPs were identified in the 110 kb region, with one in an intergenic region and the other in the exon of *MELO3C016916* (Table S2). A dCAPS2 marker was developed based on the SNP in *MELO3C016916*, which showed cosegregation with the semidwarf phenotype among all 1548 F_2_ plants used in this study, suggesting that *MELO3C016916* is a candidate gene for *Cmsi* (Fig. 2a).

### CmSI is a homolog of ERECTA family receptor kinases

The genomic DNA sequence of *MELO3C016916* in the DHL92 melon reference genome is 7128 bp, which was predicted to have 27 exons (Fig. 2b). The full length of the coding sequence (CDS) of *MELO3C016916* was 2976 bp, encoding a protein with 991 amino acid residues. The single nucleotide substitution from T to G in the 25th exon introduced a premature stop codon, which led to a truncated protein in M406 (Fig. 2c). Gene prediction and functional annotation revealed that *MELO3C016916* encoded an LRR receptor-like serine/threonine protein kinase ERECTA (Fig. S1a). Sequence alignment of CmSI and other members of the ERECTA protein family from *Arabidopsis* (AtERECTA, AtELK1, and AtELK2) and cucumber (CsERECTA) showed that CmSI shared the highly conserved leucine-rich repeat N-terminal domain (LRRNT), the LRR repeat region, and the S_TKc domain with CsERECTA (Fig. S1a). The amino acid sequence identity of CmSI to CsERECTA, AtERECTA, AtELK1, and AtELK2 was 98.99, 78.73, 62.20, and 61.09%, respectively (Fig. S1a).

To investigate the evolutionary relationship between CmSI and other ERECTA family proteins, a neighbor-joining phylogenetic tree was developed using protein sequences from 34 species (Fig. S1b). The phylogenetic tree of the ERECTA family can be divided into two main groups: monocotyledons and dicotyledons. CmSI was clustered within the dicotyledon group, which includes known ERECTA-like protein kinases, such as AtERECTA (*Arabidopsis thaliana*), PtERECTA (*Populus trichocarpa*), and VvERECTA (*Vitis vinifera*) (Fig. S1b). These results indicated that CmSI may have a similar function to other ERECTA proteins in melon.

### CmSI is highly expressed in the stem vascular bundle and ovary

**Fig. 3:**
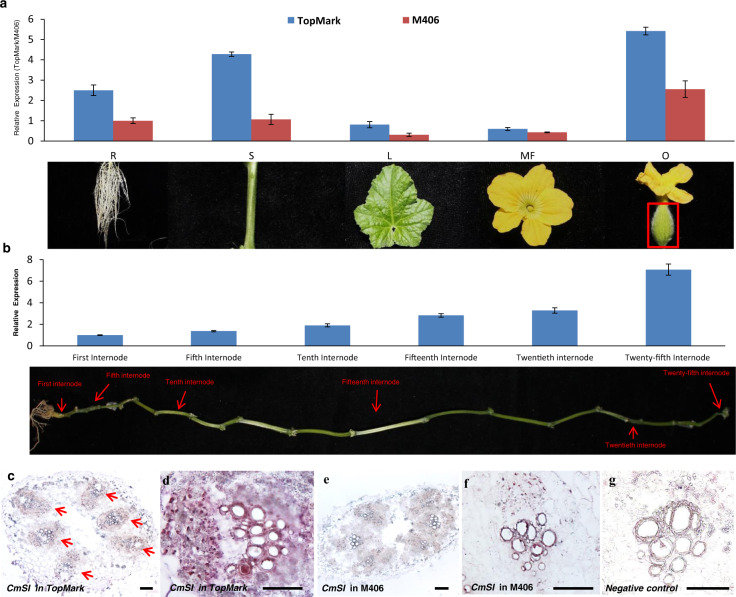
Expression analysis of *CmSI*. **a** qRT-PCR analysis of *CmSI* expression in different organs of melon. **b** *CmSI* expression in stem internodes at different developmental stages. The melon gene ACTIN was used as the internal control. Error bars indicate standard deviations of three biological replicates. R root, S stem, L leaf, MF male flower, O ovary on the day of flowering. **c**–**f** mRNA in situ hybridization of *CmSI* in stems at 1 week after transplanting. *CmSI* is highly expressed in the vascular bundle of TopMark stems (**c**, **d**) and is decreased in stems of the M406 mutant (**e**, **f**). **g** Negative controls hybridized in stem with the sense probe. Bars = 100 µm in **c**–**g**

We examined the temporal-spatial expression of *CmSI* in roots, stems, leaves, male flowers, and ovaries of TopMark and M406 using qRT-PCR. The expression level of *CmSI* was lower in all the tested organs in the mutant M406 than in TopMark (Fig. 3a). The highest expression of *CmSI* was detected in stems and ovaries of both lines (Fig. 3a). Furthermore, we analyzed the transcript level of *CmSI* in different internodes of the main stems of WT plants. The transcripts of *CmSI* were abundantly accumulated in the young internodes (upper part) but were rapidly reduced as the internode elongation stopped (Fig. 3b).

We further examined the expression pattern of *CmSI* in young stems of both TopMark and M406 with RNA in situ hybridization. *CmSI* transcripts were also detected in the epidermis and the vascular bundle of TopMark stems (Fig. 3c, d) but not in the vascular bundles of M406 stems (Fig. 3e, f), which was consistent with the qRT-PCR results. These observations suggested that *CmSI* may be involved in regulating internode elongation in melon.

### Nucleotide variation in CmSI among melon varieties

**Fig. 4:**
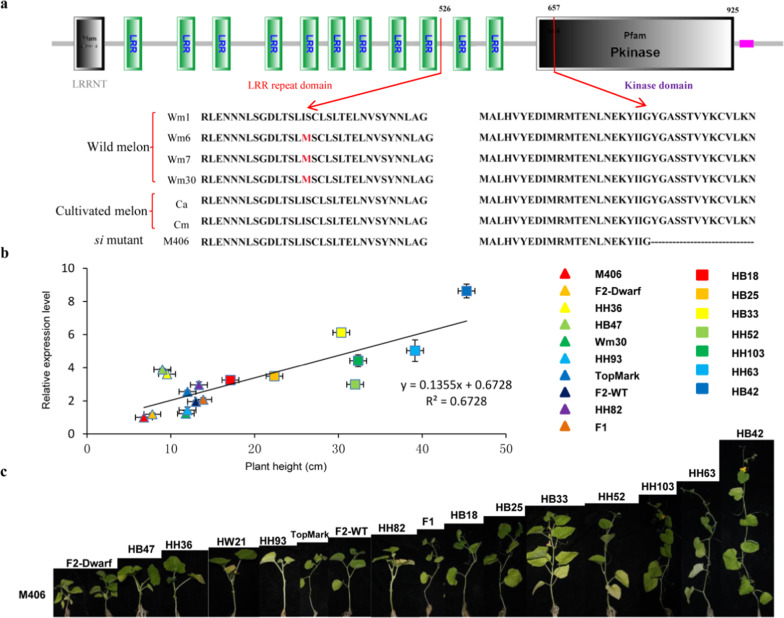
Allelic diversity and expression pattern of the *CmSI* gene in different melon germplasms. **a** Nucleotide variation at the *si* locus among 200 resequenced lines. The *si* allele was found in only M406, which suggested that the *si* allele was a spontaneous mutation and was not under selection during domestication or diversifying selection. **b**, **c** The expression pattern of the *CmSI* gene in 14 different melon germplasms and F_1_ and F_2_ of TopMark and M406. High *CmSI* expression levels were closely correlated with increased plant height among 14 different melon lines

To examine the allelic diversity of the *CmSI* gene in natural melon populations, we examined the nucleotide variation of the *CmSI* locus among 200 resequenced accessions, including 36 wild melons. The 164 cultivated accessions were further classified into two subspecies: 66 subsp. *melo* accessions and 98 subsp. *Agresti*s accessions. Eight SNPs were identified within the coding sequence of *CmSI*, of which seven were synonymous and only one was nonsynonymous (Fig. 4a). Interestingly, the only polymorphism resulting in amino acid substitutions occurred only in the wild melon group. However, there was no obvious difference in plant height between the wild melon group and cultivated melon group, indicating that this mutation may not be in the functional domains and did not affect internode elongation. Moreover, the *Cmsi* mutant allele was only found in the dwarf line M406, indicating that the *Cmsi* allele was a spontaneous mutation that was not under selection during domestication or diversifying selection (Fig. 4a).

The relationship between plant height and the expression level of *CmSI* was also analyzed in another 12 melon varieties, the F_1_ and F_2_ plants of TopMark and M406. The expression levels of *CmSI* were the lowest in M406 and the F_2_ semidwarf plants, indicating that the *Cmsi* special mutation caused a decreased expression level of the *CmSI* gene. The relationship between the *CmSI* expression level and plant height in different melon varieties was further demonstrated by the observed correlation of *CmSI* expression level and plant height (*R*^2^ = 0.6728; Fig. 4b, c). This further confirmed that CmSI functions in an expression-dependent manner in plant height regulation.

### Ectopic overexpression of CmSI increases plant height in Arabidopsis and cucumber

**Fig. 5:**
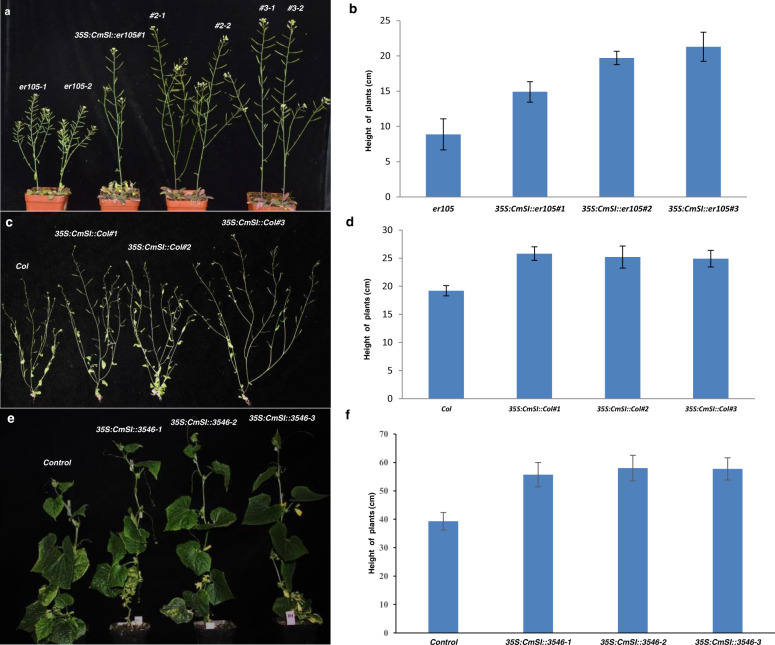
Ectopic expression of *CmSI* in *Arabidopsis* and cucumber. **a**, **b** Phenotypic comparison and statistical analysis of plant height of *er105*, 35S:*CmSI*::*er105* lines of *Arabidopsis*. Overexpression of *CmSI* in *er105* rescues the dwarf phenotype. **c**, **d** Phenotypic comparison and statistical analysis of plant height of *col*, 35S:*CmSI*::*col* lines of *Arabidopsis*. Overexpression of *CmSI* in wild-type col can also increase plant height. **e**, **f** Phenotypes and statistical analysis of *35S*:*CmSI* transgenic cucumber plants. Overexpression of *CmSI* increases plant height in cucumber

To investigate the functional conservation of *CmSI* in other plants, *35S:CmSI* overexpression vectors were constructed and then introduced into the *Arabidopsis ERECTA* mutant *er105*. The 35S promoter was used instead of the *AtERECTA* promoter because *AtERECTA* showed a different expression pattern from *CmSI*^29,38^. Seven independent transgenic lines were obtained, and three representative overexpression (OE) lines (*35S:CmSI::er105#1*, *35S:CmSI::er105#2* and *35S:CmSI::er105#3*) were selected for detailed analysis. We observed that all three OE lines partially rescued the dwarf phenotype of the *er105* mutant (Fig. 5a); their plant heights were 68, 160, and 134% higher in lines 1, 2, and 3 than in the *er105* mutant (Fig. 5b). To further validate the function of *CmSI*, we also introduced the overexpressed *CmSI* construct into WT *Arabidopsis* (Col). In all six independent transgenic lines, the plant height was higher than that in the WT (Fig. 5c, d). These data confirmed the function of *CmSI* in promoting internode elongation and increasing plant height. These observations suggest that homologs of *CmSI* may perform similar functions in *Arabidopsis* and melon.

Considering the difficulty of transformation technology in melon, we transformed the *CmSI* overexpression construct into the WT cucumber line 3546. Eight overexpression transgenic lines were generated, and the expression level of *CmSI* was much higher in three representative overexpression lines (*35S:CmSI::3546#1*, *35S:CmSI::3546#2*, and *35S:CmSI::3546#3*) than in the WT line (Fig. S2a). We observed that overexpression of *CmSI* increased the plant height in cucumber due to increased internode length (Fig. 5e, f and Fig. S2b). However, there was no significant difference in the total number of internodes and the diameter of the main stem in the control plants and that in *35S:CmSI* transgenic plants (Fig. S2c, d).

### Transcriptome profiling reveals important roles of auxin in internode elongation

To reveal the regulatory network of internode development, two RNA-Seq datasets were analyzed using the eighth internode of the main stem. One dataset was from the comparison of the transcriptomes of the eighth internodes of TopMark and M406 plants. The second dataset is from the comparison between transcriptomes of the D-bulk (dwarf) and N-bulk (WT) plants, which were developed by pooling dwarf and WT bulks in F_2_ plants (see “Experimental procedure” for details). Ten RNA-Seq libraries were subjected to high-throughput sequencing, which generated an average of 6.84 and 17.07 Gb clean data for the two parental lines and two bulks, respectively (Fig. S3a and Table S3). Using a false discovery rate (FDR) of 0.05 as the cutoff, 643 down- and 633 upregulated differentially expressed genes (DEGs) were identified in the semidwarf mutant M406 compared with the WT, respectively (Fig. S3b and Table S4). Compared with the N-bulk (WT), among 1161 DEGs, 558 and 603 were up- and downregulated, respectively, in the D-bulk (Fig. S3b and Table S5). From the two datasets, 228 common DEGs with the same expression patterns were identified, including 94 up- and 134 downregulated DEGs (Fig. S3b and Table S6).

**Fig. 6:**
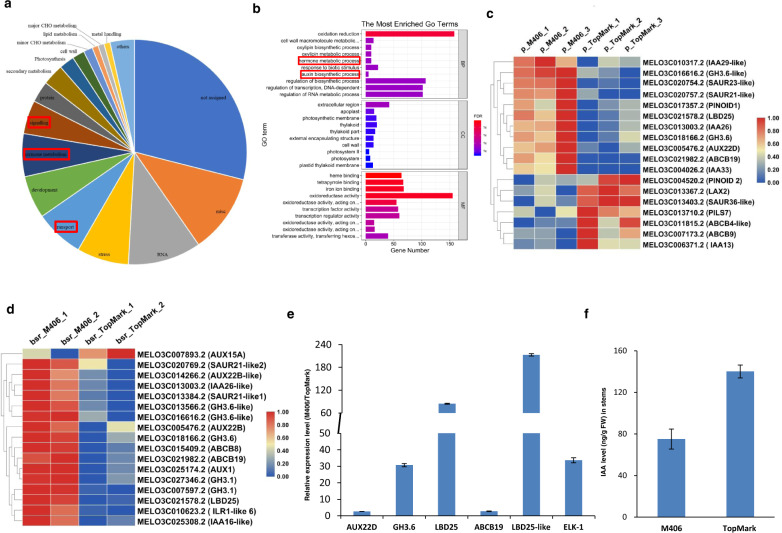
CmSI alters the expression of auxin-related genes and the auxin content in stems of M406. **a** Functional categories for common DEGs. **b** GO enrichment analysis of DEGs in two bulks. **c**, **d** Heat maps of auxin-related genes that were differentially expressed in the M406 mutant (**c**) and dwarf bulks (**d**). **e** Quantitative RT-PCR of auxin-related genes in stems of TopMark and M406 mutants. **f** IAA concentration in the stems of TopMark and M406 mutant plants

To analyze the functions of these DEGs, functional categorization of the DEGs was carried out by MapMan and Gene Ontology (GO) term enrichment analyses. MapMan analysis revealed that a large number of these DEGs were related to “hormone metabolism”, “transport”, “development”, and “signaling”, which were the most significantly enriched between two parental lines, two bulks and common DEGs (Fig. 6a and Tables S4–6). The GO enrichment analysis of the DEGs also identified various hormone metabolism-related terms, particularly those related to auxin biosynthetic processes, which were significantly enriched in the DEGs of the two parental lines and two bulks (Fig. 6b and Tables S4–6). A number of DEGs identified in this study have been shown to be involved in the auxin signaling pathway, including several auxin biosynthesis and polar transport genes, such as *PINs* and *ABCBs* (Fig. 6c, d). Quantitative real-time PCR analysis verified that the relative expression levels of auxin biosynthesis and polar transport genes were significantly higher in the semidwarf mutant M406 than in TopMark (Fig. 6e). Moreover, we measured the auxin levels in the stems of the two parental lines. As expected, the auxin concentration was significantly reduced in the semidwarf mutant M406 (Fig. 6f), which is consistent with the promoting role of auxin in internode elongation. These results suggested that CmSI may affect auxin transport or signaling in stems.

### CmSI interacts with CsPIN2 at the protein level

**Fig. 7:**
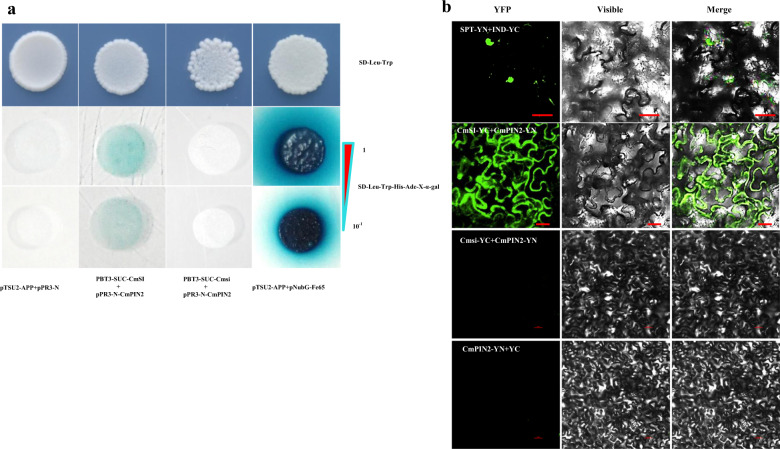
Yeast two-hybrid assay for the physical interaction between CmSI and CmPIN2. **a** The protein interaction was examined using various combinations of prey and bait vectors. The vector pPR3-N and pTSU2-App was used as a negative control, and the interaction between pTSU2-APP and pNubG-Fe65 was used as a positive control. Yeast cells were grown on SD/-Leu-Trp medium and interactions were confirmed by an SD/-Leu-Trp-His-Ade-X-a-GAL assay on medium. Dilutions (1 and 10^−1^) of saturated cultures were spotted onto the plates. Yeast two-hybrid assay showing that CmSI rather than Cmsi interacted with CmPIN2 by growth on SD/-Leu/-Trp/-His/-Ade/X-α-gal plates. **b** BiFC analysis of the physical interaction between CmSI and Cmsi (fused with the C-terminal fragment of YFP) and CmPIN2 (fused with the N-terminal fragment of YFP). INDEHISCENT (IND)-YFPC and SPATULA (SPT)-YFPN were used as positive controls. CmPIN2-YFPN and the empty YFPC were used as negative controls. Different combinations of the fused constructs were co-expressed in leaves of *Nicotiana tabacum*, and the cells were then visualized using confocal microscopy

To identify the putative interacting proteins of CmSI, we conducted yeast two hybridization (Y2H) assays to detect interactions of CmSI with the differentially expressed auxin-related genes and transcriptional regulators required for plant organ development, including the efflux-facilitating PIN-FORMED2 (MELO3C004520.2), three auxin response factors (MELO3C013003.2, MELO3C005476.2, MELO3C004026.2), two boundary genes (MELO3C021578.2, MELO3C026269.2), two Gretchen Hagen 3 genes (MELO3C018166.2, MELO3C016616.2), a member of pleiotropic drug resistance ABC transporter (MELO3C009686.2), five different transcription factors (MELO3C004181.2, MELO3C010984.2, MELO3C021426.2, MELO3C026299.2, MELO3C030287.2). Among them, only CmPIN2 was found to interact with CmSI (Fig. 7a). Moreover, the interactions between CmSI and CmPIN2 were further confirmed by BiFC analysis, which showed that CmPIN2 could direct interact with CmSI rather than the mutated protein Cmsi (Fig. 7b). These results suggested that CmSI may regulate stem elongation via interacting with CmPIN2 in melon.

## Discussion

### Identification and map-based cloning of a novel semidwarf mutant in melon

Plant height is an important agronomic trait affecting crop architecture, resistance to lodging, tolerance to crowding, fruit yield, and mechanical harvesting. Almost all commercial melon varieties have long main stems and many lateral branches, which requires pruning in standard cultivation practices. Pruning of these plants is very labor intensive and time consuming. Therefore, the development of semidwarf cultivars with short branches is an important objective in melon breeding, especially for production under protected environments. To date, four recessive dwarf/semidwarf mutants, *si-1*, *si-2*, *si-3*, and *mdw1*, have been identified^35,37^, but only *mdw1* was genetically mapped on Chr7 (ref. ^35^). In this study, *Cmsi* was also mapped on Chr7 in a 110.00 kb region, and *CmERECTA* was identified as the candidate gene (Fig. 2). Hwang et al. (2014) found that the *mdw1* locus was closely linked with the *mERE* gene, which is the same as *CmERECTA* (*MELO3C016916*). Therefore, it is reasonable to speculate that *Cmsi* and *mdw1* probably belong to the same locus, but whether the two mutants have the same mutant allele is not known. The only SNP in the *CmERECTA* gene between the two parental lines located in the exon resulted in a premature termination codon in the semidwarf line M406. The SNP-derived dCAPS2 marker cosegregated in the F_2_ population. Furthermore, we examined the diversity of nucleotides and amino acids at the *CmSI* locus in a panel of 200 melon accessions of the global collection. The SNP allele in *CmERECTA* was only present in the mutant M406 among accessions from wild melon and cultivated melons. It should be noted that there were also seven other SNP variants among the 200 melon accessions. All seven SNPs were synonymous mutations in 164 cultivated melon accessions. Although there was only one nonsynonymous SNP in some wild melon accessions, the mutant allele was not associated with stem length variation (Fig. 4), suggesting that this SNP site is probably not located in the functional domain. Therefore, the cosegregated marker dCAPS2 of *CmSI* will be very useful in marker-assisted selection for use of this *Cmsi* allele in melon breeding.

### CmSI encodes an ERECTA-like protein of the leucine-rich repeat (LRR) subfamily

The ERECTA gene family, *ERECTA*, *ERL1*, and *ERL2*, which encode leucine-rich repeat receptor-like kinases, has been found in many monocots and dicots. These genes play important roles in different developmental processes, including inflorescence architecture, hypocotyl development, seed germination, ovule development, and stomatal and trichome formation^34,39–41^. We show that *CmSI* encodes an ERECTA protein (Fig. 2) that shares a high level of sequence identity with the AtERECTA protein (Fig. S1). Overexpression of *CmSI* in *Arabidopsis* and cucumber increased their plant height (Fig. 5). This convincing evidence suggests that *CmSI*, like other ERECTA-like homologs, regulates cell development and stem internode elongation in melon. The expression of *CmSI* was much higher in the young internodes during internode elongation and declined rapidly when internode elongation stopped (Fig. 3b). Moreover, the *CmSI* transcript was more abundant in stems and ovaries than in other tissues in both WT and semidwarf plants (Fig. 3). In previous studies, the expression levels of grape *VvERECTA* and tomato *SlERECTA* were higher in young fruits than in mature fruits^40,42^, indicating that *ERECTA-like* genes play important roles in early organ development in different species. However, the temporal and spatial expression pattern of *CmSI* in melon was not exactly the same as that of *AtERECTA* in *Arabidopsis*. The expression of *AtERECTA* was higher in developing above-ground organs such as flowers and young rosettes but lower in stems and almost undetectable in roots^29,38,43^. In our study, the expression level of *CmSI* was high in roots and stems, indicating that *CmSI* and *AtERECTA* may play distinct roles in different developmental processes between the two species. Moreover, the semidwarf mutant M406 has shorter primary roots than the normal line TopMark, indicating that *CmSI* also affects root development in melon (Fig. S4).

In *Arabidopsis*, *AtERECTA* has partial functional redundancy with its two homologous genes *ERL1* and *ERL2* in hypocotyl and stem elongation, leaf serration development, and stoma and trichome formation. The triple mutant *ererl1erl2* intensifies the *erecta* mutant phenotype, and the *ererl1erl2* plant is extremely dwarfed compared with *erecta*^29,44^. Compared with the WT TopMark, the length of the main stem and lateral branches was significantly decreased in the mutant M406 (Fig. 1). However, the trichome numbers on the stems of the semidwarf mutant did not show a significant change (Fig. S5c, d). In this study, we found that the transcript of the *CmERL1* gene was significantly increased in the mutant M406 (Fig. 6e and Fig. S5b), indicating that *CmERL1* may play a redundant role with *CmSI* in melon trichome development.

### CmSI regulates stem elongation through the auxin pathway

Plant height is regulated by multiple genes and associated complex regulatory networks. As a classical phytohormone, auxin plays important roles in regulating key agricultural traits associated with plant height and shoot branching^16,45^. Previous studies have shown that overexpression of the *Arabidopsis* auxin biosynthesis gene *YUCCA5* can rescue the dwarf phenotype of the *ERECTA* mutant *er-103* (ref. ^33^). Increasing endogenous or exogenous auxin levels could also partially rescue short hypocotyl defects of the *ererl1erl2* triple mutant^34^. Here, transcriptomic analysis showed that “auxin biosynthetic process” and “auxin metabolic process” were the most significantly enriched GO terms for these DEGs in stem elongation (Table S4), and the functions of a number of DEGs were related to “hormone metabolism”, “transport”, and “signaling”, suggesting that auxin plays an important role in regulating internode elongation in melon. Moreover, the auxin content was decreased in the stem of M406, and the cell size in the stem of semidwarf plants was also smaller than that of TopMark. This evidence indicated that *CmSI* may regulate stem elongation by affecting the auxin content in melon stems. From the Y2H assay, we found that CmSI directly interacted with the efflux-facilitating PIN-FORMED protein CmPIN2. The homologs of CmPIN2 in rice, maize, and many other species have been shown to positively regulate auxin transport during organ formation^23–25^. Overexpression of *OsPIN2* in rice can enhance auxin transport from stems to roots and thus decrease plant height^27^. *ZmPIN1a* overexpression also increased IAA transport from shoots to roots and reduced plant height, ear height, and internode length in maize^26^. More importantly, CmSI rather than Cmsi could interact with CmPIN2, indicating that CmSI may regulate stem development by interacting with CmPIN2, and the single nucleotide mutation of *Cmsi* in the semidwarf mutant M406 disrupted the interaction between CmSI and CmPIN2. Taken together, our data elucidated a novel link between the ERECTA protein and auxin polar transport in melon.

## Experimental procedures

### Plant material and growth conditions

The *short internode* mutant line M406 displays shorter internodes and fewer lateral branches than the normal line. To confirm the inheritance mode and fine mapping of the *Cmsi* gene, M406 was crossed with the WT muskmelon inbred line TopMark to generate a large F_2_ mapping population. The *χ*^2^-test for goodness-of-fit was used to test for deviation of the observed data from the theoretically expected segregation for semidwarf phenotype data in F_2_ plants. All plant materials were grown in greenhouses at the Maozhuang Research Station of Henan Agricultural University (Zhengzhou, China). The plant height (stem length) of each plant was qualitatively recorded as either normal or mutant at 30 and 60 days after transplanting. In addition, another 12 melon inbred lines from different regions and a monoecious cucumber (*Cucumis sativus* L.) inbred line 3546 (WT) used in this study were also cultivated in a greenhouse under normal conditions. The *Arabidopsis* mutant *er105* (Col background) and Col WT were used for functional complementary verification. *Arabidopsis* seeds were germinated on Murashige–Skoog (MS) medium containing 0.2% Phytagar and 1% sucrose. The seeds were kept at 4 °C for 3 days and then moved to 22 °C under a 16 h light/8 h dark light regime. Seedlings were transferred to soil 7–10 days after germination.

Unexpanded young leaves from test plants were collected into 1.5 mL microcentrifuge tubes, lyophilized in a freeze dryer, and ground into fine powder. Genomic DNA was extracted using the CTAB method^34^.

### Microscopic examination of internodes

To compare the microscopic structure of internodes in WT and mutant plants, the eighth internodes of the stems of 40-day-old plants of TopMark and M406 were fixed, rinsed, postfixed, washed, dehydrated, and embedded. Semithin sections were prepared with Formvar-coated gold grids and observed with an Olympus-BX53 light microscope as previously described^46^.

### Molecular marker analysis and fine mapping

For initial mapping of *Cmsi*, a linkage map was developed using 92 TopMark × M406 F_2_ plants. Linkage analysis placed the *Cmsi* locus on the short arm of melon chromosome 7 (LGVII) flanked with CmSSR17145 and CmSSR17293, which were used as the starting point for fine mapping. In the target region, we first explored SSR markers^47^. Additional SNPs and Indels were identified through bioinformatics analysis of resequencing reads of TopMark and M406. The resequencing of TopMark and M406 was carried out following the standard Illumina protocol, and the library was used for paired-end sequencing on the Illumina HiSeq2500 analyzer. After removing short reads and low-quality reads, the clean reads from the two parental lines were used for mapping to the melon reference genome DHL92 (https://melonomics.net/)^48^ using BWA software^49^. SNPs and small Indels detected from the alignments were called using Samtools, and output was given in pileup format^50^. The SNPs and small Indels between two parental lines were detected using the GATK software tool package^51^, and reliable SNPs and small Indels were noted and predicted using SnpEff software^52^. Only those Indels with ≥3 bp differences were selected for primer design with Primer3 software (http://primer3.ut.ee/), and dCAPS markers were developed for SNP genotyping by dCAPS Finder 2.0 (ref. ^53^).

Polymorphic SSR and Indel markers were used for chromosome walking and fine mapping in a large population containing 1261 F_2_ plants. Finally, additional dCAPS makers were used for genotyping those F_2_ plants to identify recombinants identified from 1548 F_2_ plants. The PCR amplification of molecular markers and subsequent gel electrophoresis were performed as previously described^47^. Linkage analysis of the *Cmsi* locus with molecular markers was performed with the Kosambi mapping function using JoinMap 3.0.

### Sequence alignments and phylogenetic analysis

The coding sequence (CDS) of *CmSI* was amplified by PCR from stem cDNA using gene-specific primers (Supplementary Table S7). The amino acid sequences of the related ERECTA-like proteins from *Arabidopsis*, cucumber, and other species were obtained by BLAST searches of the National Center for Biotechnology Information nucleotide database (http://www.ncbi.nlm.nih.gov/nucleotide/). A multiple sequence alignment of CmSI and the related ERECTA-like proteins was carried out as previously described^54^. A phylogenetic tree was developed using the neighbor-joining (NJ) method^55^ in the MEGA5 software package.

To examine the allelic diversity of the *CmSI* gene in natural melon populations, the clean reads of 200 resequenced melon accessions were aligned to the reference sequence of the *CmSI* gene (DHL92 draft genome) for SNP calling as previously described^56^. Furthermore, the plant height of five plants from each of the 200 melon accessions was recorded as normal height or dwarf height at 30 and 60 days in the field in 2017 and 2018.

### Spatial and temporal expression analysis by quantitative real-time PCR

Total RNA of the stems, roots, leaves, male flowers, ovaries, and internodes was extracted using the Quick RNA isolation kit (Huayueyang, China) and reverse transcribed to first strand cDNA using the PrimeScript First Strand cDNA Synthesis Kit (Takara). SYBR® Premix Ex Taq from TaKaRa was used for qPCR with the Applied Biosystems StepOne™ Real-Time PCR System. The melon *ACTIN* gene was used as the internal control^57^ in all qPCR reactions. All experiments were performed with three biological and three technical replicates. The *CmSI*-specific qPCR primers are listed in Supplementary Table S7.

### RNA in situ hybridization

Tender stems of WT and mutant plants were fixed, dehydrated, dewaxed, embedded, sectioned, and hybridized with digoxigenin-labeled probes as previously described^58^. Digoxigenin-labeled sense and antisense RNA probes were obtained using T7 and SP6 RNA polymerases (Roche). The primer pairs used are listed in Supplementary Table S7.

### Ectopic expression of CmSI in *Arabidopsis* and cucumber

The full-length coding regions of *CmSI* were cloned without the stop codon and inserted into the SuperpCAMBIA1300 vector^59^ between the *Spe*I and *Sma*I sites. The *CmSI*-SuperpCAMBIA1300-overexpressing vector was transformed into *er105* mutant and Col (WT) plants using the floral dip method^60^. The transgenic *Arabidopsis* plants were screened on Murashige and Skoog (MS) medium with 25 mg l^–1^ hygromycin.

The *CmSI*-SuperpCAMBIA1300 fusion vector was transformed into cucumber line 3546 (WT) using a cotyledon transformation method as previously described^61^. In brief, the Agrobacterium strain AGL1 was transformed with the *CmSI*-SuperpCAMBIA1300 fusion vector and used to transform embryonic callus of cucumber by cocultivation. MS medium supplemented with 10 mg/l hygromycin was used to select transformants. The positive transgenic plants were verified by PCR using specific primers. The primers are listed in Supplementary Table S7. At least three representative transgenic lines and three plants in each line were used for further analysis.

### Transcriptome analysis

To investigate the regulatory network of the *CmSI* gene, we used a strategy combining the RNA-seq of two parental lines and bulked-segregant analysis (BSR-seq) of the F_2_ population. For RNA-Seq, two bulks, the dwarf bulk (D-bulk) and the normal bulk (N-bulk), were constructed by pooling 20 dwarf and 20 WT F_2_ plants, respectively.). Total RNA of the main stems of the two bulks and the eighth nodes of both parents were extracted and used for strand-specific RNA-Seq library construction and next-generation sequencing on an Illumina HiSeqTM 4000 platform. Three biological and two technical replications were sequenced for the parental lines and bulks, respectively.

After removing the adapters and low-quality reads, the clean data were used for alignment to the melon reference genome DHL92 using TopHat v2.1.1 (ref. ^62^). The read numbers of annotated genes were counted by the HTSeq program (v0.9.1)^63^. The number of transcripts per million reads (TPM) for each gene was calculated based on the length of the gene and mapped read counts. DEGs between two parental lines and two bulks were identified using the DEGSeq R package (1.12.0). A corrected *P* value of 0.05 was set as the threshold for DEG selection. GO terms for these DEGs were determined using InterProScan program^64,65^. Then, GO functional enrichment analysis was performed to identify DEGs with significantly enriched GO terms. The GO analysis was carried out with AgriGO with an FDR ≤ 0.05 to obtain the GO annotations based on the biological process, molecular function, and cellular component categories^66^. The functional categorization of these DEGs was classified using MapMan.

### Auxin quantitation

For measurement of the auxin content, fresh stems (50 mg) of 30-day-old plants were frozen in liquid nitrogen. Quantification of endogenous auxin levels was performed using high-performance liquid chromatography electrospray ionization tandem mass spectrometry (HPLC-ESI-MS/MS)^67^.

### Yeast two-hybrid assay

For the yeast two-hybrid assay, we cloned the CDS without an N-terminal cleavable signal sequence, and the stop codon of *CmSI* and *Cmsi* fused them into the pBT3-SUC vector. The full-length CDSs of *CmPIN2* were cloned and fused with the pPR3-N vector. The combination of pTSU2-APP and pNubG-Fe65 was used as a positive control, and the combination of pTSU2-APP and pPR3-N was used as a positive control. All recombinant constructs were separately transformed into the yeast strain NMY51. According to the DUAL membrane starter kit user manual, the transformed yeast cells were grown on synthetic defined (SD) plates lacking tryptophan and histidine (SD/−Trp−His) and lacking tryptophan, histidine, and adenine (SD/−Trp−His−Ade) with α-gal.

### Accession numbers

GenBank accession numbers of ERECTA-LIKE protein sequences used in this study included Arabidopsis ERECTA (AT2G26330), ERECTA-LIKE1 (AY244745), ERECTA-LIKE2 (AY244746), and cucumber ERECTA-LIKE (EST241733).

## Supplementary information

Supplementary Figures S1-S5.docx

Supplementary Tables S1-S7

## Data Availability

The resequencing data and transcriptome sequencing data of two parental lines and two bulks are available from the NCBI Short Read Archive (SRA PRJNA608205).
