# Supplementary material for: Melon short internode (CmSi) encodes an ERECTA-like receptor kinase regulating stem elongation through auxin signaling
Source: Hortic Res. 2020 Dec 1;7:202. doi: 10.1038/s41438-020-00426-6 (PMC7705010; doi:10.1038/s41438-020-00426-6)
Supplement: Supplementary file 1 — Supplementary Figures S1-S5.docx [file 41438_2020_426_MOESM1_ESM.docx]

**Supplementary data
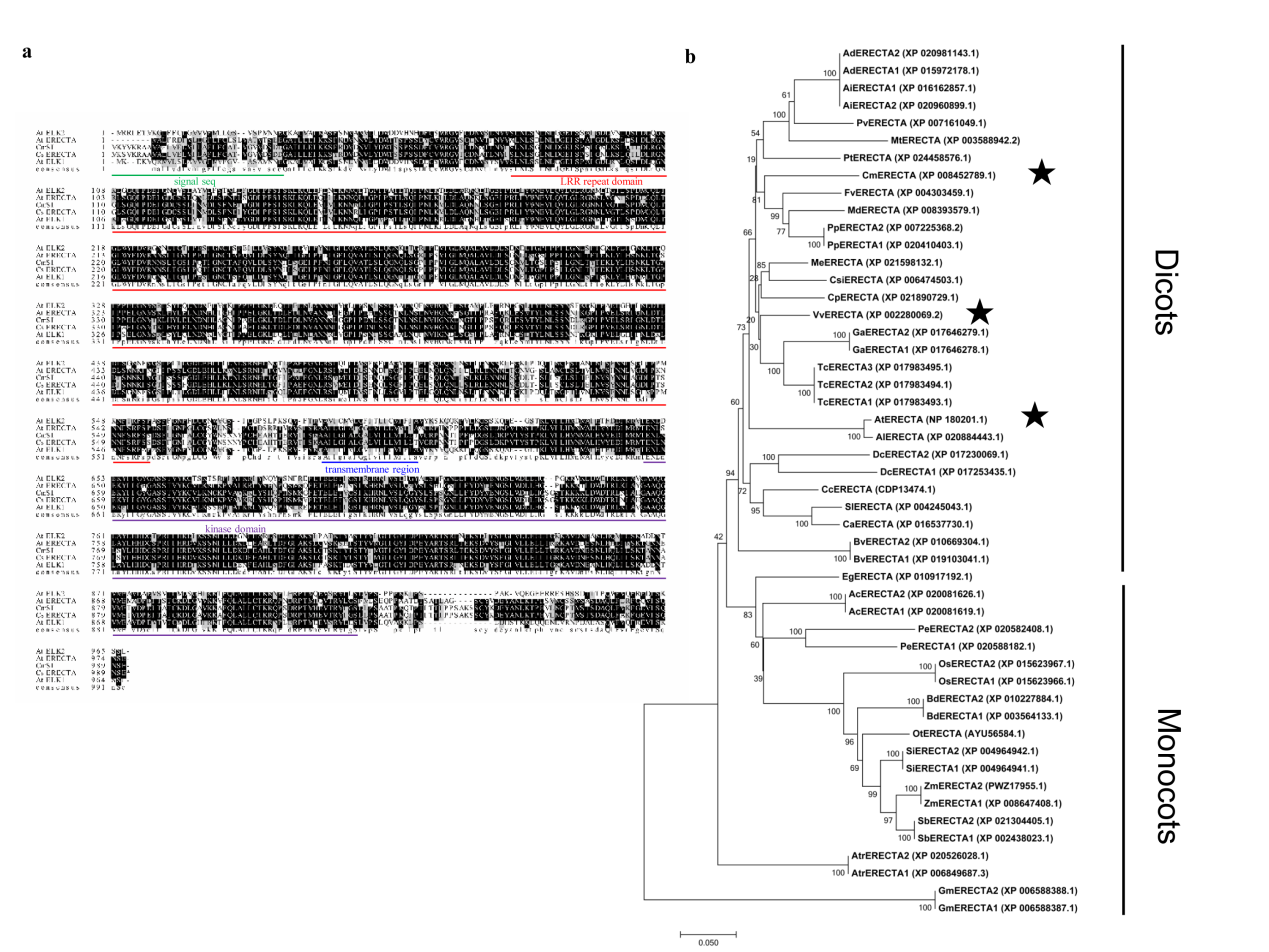
**

**Figure. S1.** Phylogenetic analyses of *CmSI* and its homologues in various species. (a) Protein sequence alignment of *CmSI* and its homologues from cucumber (CsERECTA) and Arabidopsis (AtERECTA, AtERECTA-LIKE1, AtERECTA-LIKE2). The underlined indicate the conserved domain. (b) Phylogram of CmSI and related ERECTA-LIKE proteins from *Arabidopsis thaliana, Ostreococcus lucimarinus, Amborella trichopoda, Phalaenopsis equestris, Elaeis guineensis, Ananas comosus, Oropetium thomaeum, Zea mays, Sorghum bicolor, Setaria italic, Brachypodium distachyon, Oryza sativa, Beta vulgaris, Capsicum annuum, Solanum Lycopersicon, Coffea canephora, Daucus carota, Vitis vinifera, Arachis duranensis, Arachis ipaensis, Medicago truncatula, Glycine max, Phaseolus vulgaris, Malus domestica, Prunus persica, Fragaria vesca, Manihot esculenta, Populus trichocarpa, Citrus sinensis, Theobroma cacao, Gossypium arboretum, Carica papaya, Arabidopsis thaliana, Arabidopsis lyrata, Capsella rubella.* The number at each node is the probability supporting each node with 1000 bootstrapping values.

**
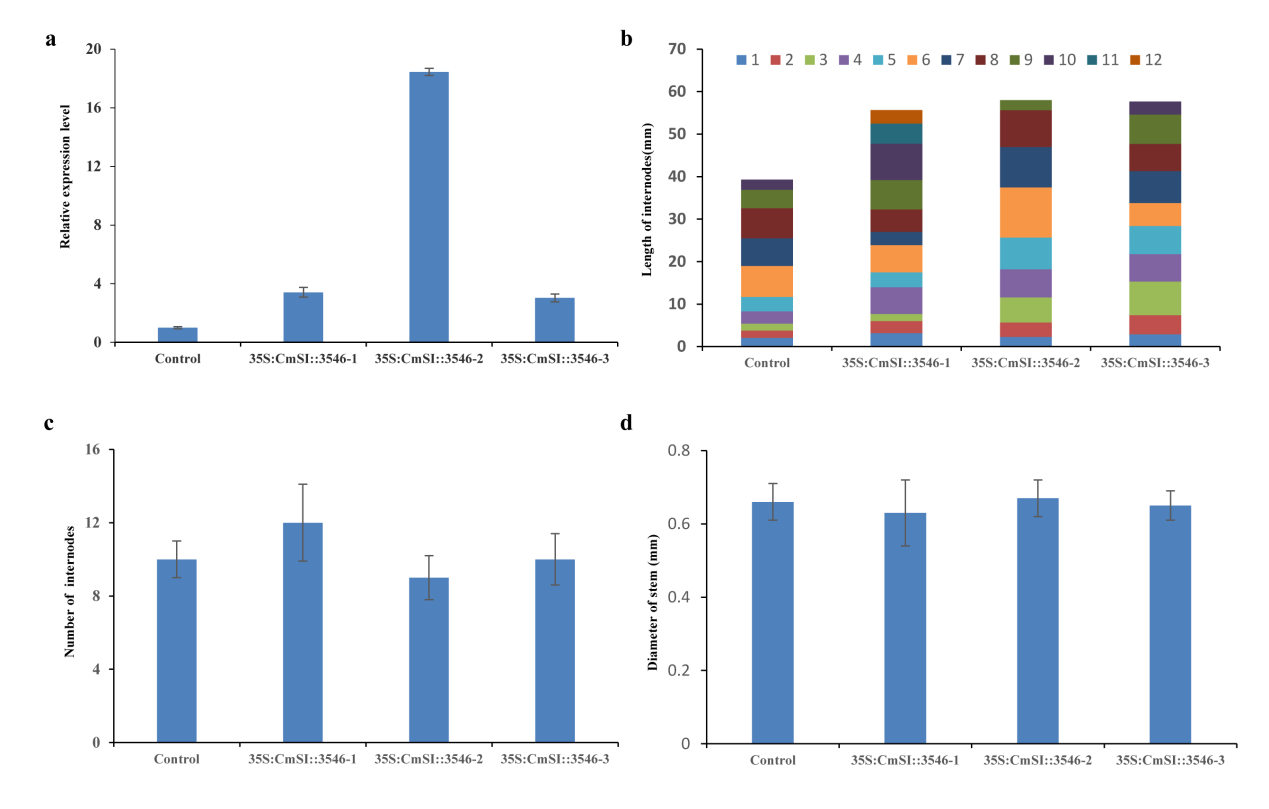
Figure. S2.** Gene expression and phenotypes analysis of three representative *35S:CmSI* transgenic cucumber plants. (a) qRT–PCR analyses of *CmSI* in control plants and transgenic overexpression lines. Overexpression of *CmSI* increased the plant height in cucumber, due to the increased internode length (b). However，there was no significant difference for total number of nodes and the diameter of the main stem in control plants and 35S:CmSI transgenic plants (c, d).


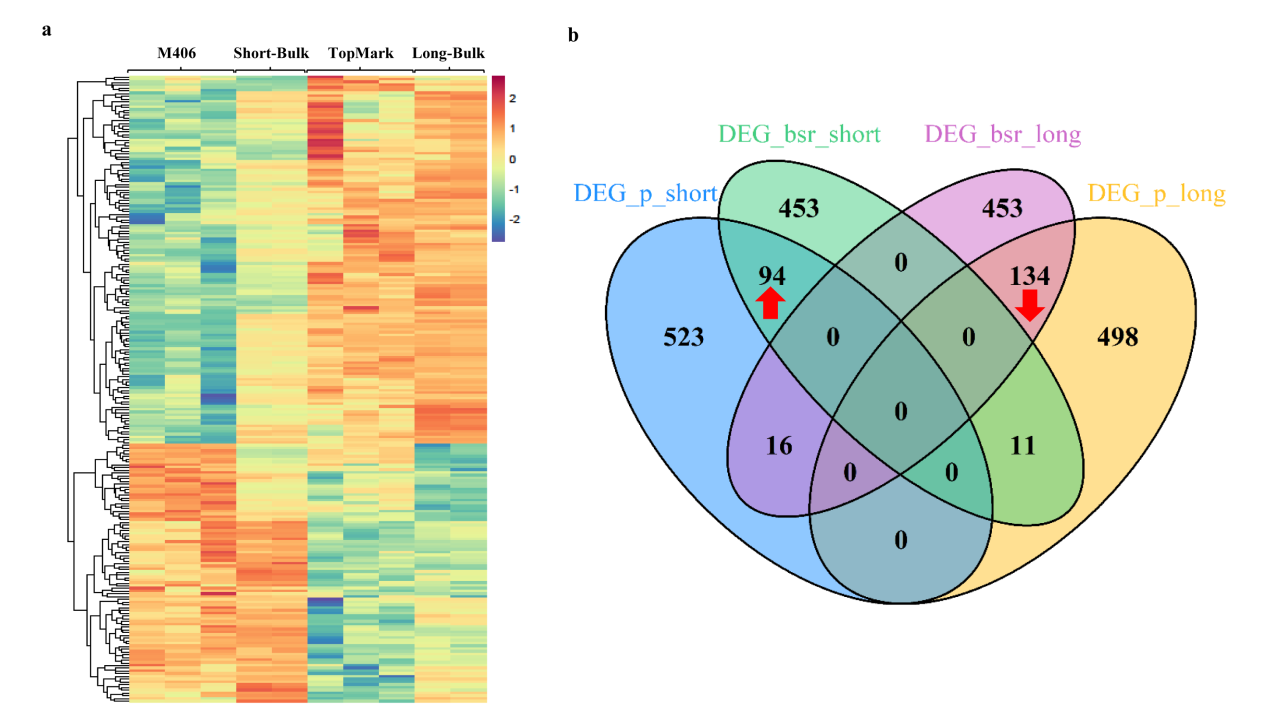


**Figure. S3.** Hierarchical clustering (a) and Venn diagram analysis (b) of the DEGs between two parental lines and two bulks.

**
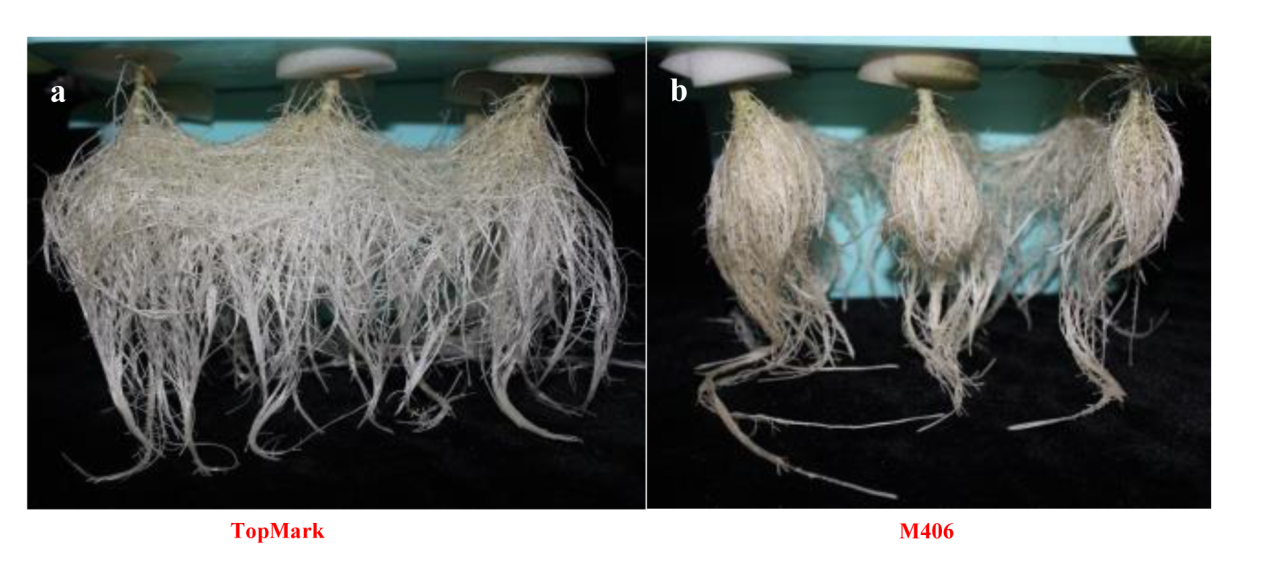
**

**Figure. S4.** Morphological characterization of roots in melon normal line TopMark (a) and semi-dwarf line M406 (b).

**
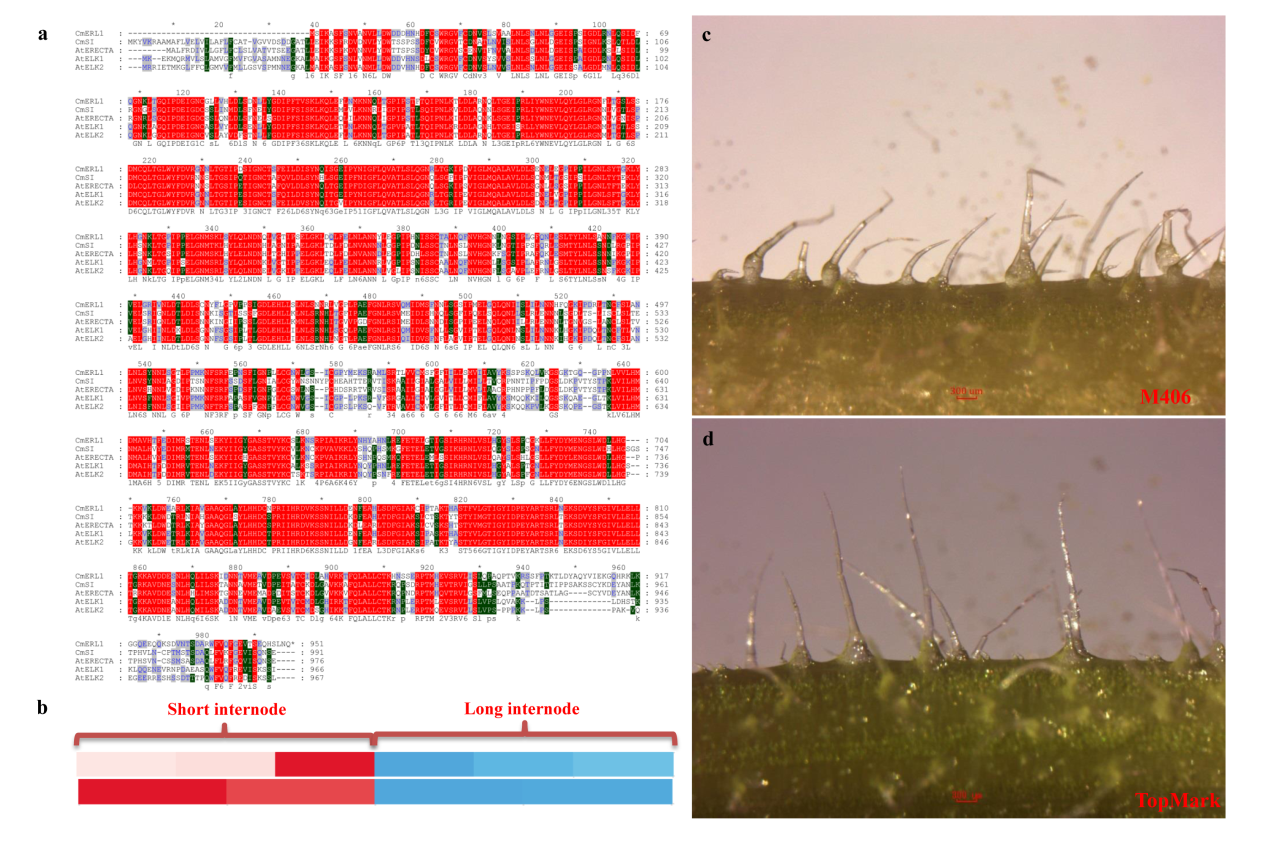
Figure. S5. Increased expression of *CmERL1* may result in the same phenotype of trichome in TopMark and M406.** (a) Protein sequence alignment of *CmERL1* and its homologues from Arabidopsis (AtERECTA, AtERECTA-LIKE1, AtERECTA-LIKE2). (b) Heat maps expression of *CmERL1* increased in M406. (c-d) The same phenotype of trichome between TopMark and M406.
