# Supplementary material for: Melon short internode (CmSi) encodes an ERECTA-like receptor kinase regulating stem elongation through auxin signaling
Source: Hortic Res. 2020 Dec 1;7:202. doi: 10.1038/s41438-020-00426-6 (PMC7705010; doi:10.1038/s41438-020-00426-6)
Supplement: Supplementary file 2 — Supplementary Tables S1-S7 [file 41438_2020_426_MOESM2_ESM.pdf]

Table S1. Segregation analysis of the normal/dwarf in the F_2 progenies.

Combinnation	Population	Total	normal	dwarf	Expected	χ^2 value[a]
M323×M406	F_1	12	12			
	F_2	1261	931	330	3:01	0.8588

$\chi 2_{(0.05, 1)} = 3.84$

Table S2. Gene Ontology of 14 predicted genes between markers dCAPS1 and dCAPS4.

Gene ID	Physical distance	Gene Ontology
MELO3C016928	1081649..1083276	Protein ROOT PRIMORDIUM DEFECTIVE 1
MELO3C016927	1089185..1089763	Ethylene-responsive Transcription Factor Erf017-like
MELO3C016926	1092715..1099611	DNA Cross-link Repair Protein SNM1
MELO3C016925	1100697..1103705	Pentatricopeptide Repeat-containing Protein At4g33170-like
MELO3C016924	110486..1109451	Smr (Small MutS-related) domain protein
MELO3C016923	1115764..1117102	Protein of unknown function (DUF1677)
MELO3C016922	1117577..1117792	DNA-directed RNA polymerase subunit omega
MELO3C016921	1122738..1127171	G-protein Coupled Receptor 1
MELO3C016920	1127899..1129846	Putative Receptor-like Protein Kinase At1g72540
MELO3C016919	1146652..1152145	Guanine Nucleotide-binding Protein Alpha-1 Subunit
MELO3C016918	1153115..1157656	Fatty-acid-binding Protein 2
MELO3C016917	1160371..1162944	Vitellogenin-2
MELO3C016916	1163457..1171401	LRR Receptor-like Serine/Threonine-protein Kinase ERECTA
MELO3C016915	1178459..1187249	Copper-transporting Atpase PAA2, Chloroplastic

Table S3 Statistics of ten RNA-seq libraries.

Sample ID	Clean Reads	Clean Bases (Gb)	Q30 (%)	GC (%)	Mapped reads (%)
TopMark-1	21025977	6.27	94.67	44.59	88.10%
TopMark-2	19928999	5.95	94.28	44.29	88.10%
TopMark-3	25449750	7.59	94.18	44.31	87.60%
M406-1	25989708	7.75	93.55	44.03	88.10%
M406-2	23583950	7.01	93.94	43.61	87.60%
M406-3	21772093	6.48	94.25	44.28	88.70%
Normal Bulk-1	42959696	12.83	94.34	44.07	88.90%
Normal Bulk-2	64567667	19.28	94.41	44.11	88.70%
Dwarf Bulk-1	61625660	18.39	92.45	44.96	87.50%
Dwarf Bulk-2	59521178	17.77	93.75	44.93	84.00%

Table S4 The DEGs information, enriched Gene Ontology terms and functional categorization of these DEGs between two parental lines

DEGs identified between two parental lines.

Gene_name	log2FoldChange	P-value	Status	Function annotation
MELO3C000027.2	-1.475127948	0.000000457	down	eukaryotic_translation_initiation_factor_3_subunit_M
MELO3C000093.2	-2.588035512	3.06E-09	down	casein_kinase_I
MELO3C000147.2	1.44214094	0.000000108	up	Aaa-atpase
MELO3C000200.2	-1.546921553	0.003713017	down	Bidirectional_sugar_transporter_SWEET
MELO3C000213.2	1.68010636	1.37E-16	up	Rab_proteins_geranylgeranyltransferase_component_A
MELO3C000237.2	1.418241436	0.003978294	up	Major_facilitator_superfamily_domain-containing_protein_12
MELO3C000296.2	8.085307828	1.95E-10	up	DnaJ_like_protein
MELO3C000298.2	-1.499662539	0.00000117	down	Protein_trichome_birefringence
MELO3C000325.2	5.739766187	0.0000527	up	DnaJ-like_protein
MELO3C000335.2	3.438032391	0.0000158	up	MRGH13
MELO3C000336.2	-1.251140529	0.002652248	down	Transcription_factor
MELO3C000413.2	1.535775711	1.15E-12	up	Cytosolic_Fe-S_cluster_assembly_factor_NARFL
MELO3C000589.2	-1.991634341	0.002661773	down	Kinase_family_protein
MELO3C000609.2	-1.674383244	0.002253205	down	HMG-Y-related_protein_A
MELO3C000668.2	-1.808914277	0.001092705	down	Protein_DETOXIFICATION
MELO3C000849.2	1.328426204	0.000806569	up	Subtilisin-like_protease
MELO3C000897.2	-9.092658589	1.44E-13	down	BnaA07g12620D_protein
MELO3C000909.2	6.366467111	0.000417073	up	Ankyrin_repeat_family_protein,_putative
MELO3C000922.2	-2.203374008	0.00294319	down	NAC_domain-containing_protein,_putative
MELO3C000994.2	6.020306169	0.0000721	up	wall-associated_receptor_kinase_2-like
MELO3C001014.2	1.012748798	0.001357803	up	protein_NRT1/_PTR_FAMILY_4.6-like
MELO3C001058.2	-4.954551531	0.002171589	down	Lipoxygenase
MELO3C001150.2	-2.240455695	0.000187064	down	Non-heme_dioxygenase_N-terminal_domain-containing_protein
MELO3C001165.2	-1.347035641	0.000489632	down	eukaryotic_translation_initiation_factor_3_subunit_M
MELO3C001244.2	-7.034067778	0.0000112	down	Unknown_protein
MELO3C001321.2	-1.258734513	0.001161588	down	Unknown_protein
MELO3C001365.2	2.868970999	0.000453694	up	Unknown_protein
MELO3C001419.2	3.782433957	1.28E-35	up	Aaa-atpase
MELO3C001494.2	-6.356779604	0.0000403	down	Pectinesterase
MELO3C001614.2	-4.658883261	3.66E-11	down	Class_I_heat_shock_protein
MELO3C002020.2	-1.619608451	0.0000424	down	Chaperone_protein
MELO3C002064.2	1.236740337	0.001837199	up	phospholipid-transporting_ATPase_10-like
MELO3C002072.2	1.078098295	0.0000407	up	Adenine_nucleotide_alpha_hydrolases-like_superfamily_protein
MELO3C002076.2	5.673859381	0.002833359	up	Transcription_factor_TEOSINTE_BRANCHED_1
MELO3C002090.2	-1.912458249	0.00000304	down	transcription_factor_MYB108-like
MELO3C002101.2	-1.289452222	0.000902895	down	Receptor-like_kinase
MELO3C002161.2	10.24051176	2.89E-17	up	SufE-like_protein_1,_chloroplastic/mitochondrial
MELO3C002189.2	2.233051647	5.34E-10	up	Alcohol_dehydrogenase_family_protein
MELO3C002190.2	-1.188957003	0.0000272	down	Protein_DETOXIFICATION
MELO3C002191.2	-5.837193136	0.000326224	down	Short-chain_dehydrogenase/reductase
MELO3C002220.2	1.194935483	0.000000469	up	AAA-ATPase_At3g28580-like
MELO3C002221.2	1.855895691	0.004201009	up	AAA-ATPase_At3g28580-like
MELO3C002224.2	1.144104031	3.93E-09	up	Dynein_light_chain
MELO3C002253.2	1.008431842	0.004310748	up	RING-type_E3_ubiquitin_transferase
MELO3C002272.2	1.351372719	8.27E-09	up	Transmembrane_protein,_putative
MELO3C002280.2	-1.483996161	3.43E-08	down	LRR_receptor-like_serine/threonine-
MELO3C002282.2	-1.396139471	1.81E-11	down	Nucleoside_phosphatase_GDA1/CD39
MELO3C002286.2	1.808080611	1.88E-12	up	Receptor_protein_kinase,_putative
MELO3C002310.2	-8.317081032	3.49E-09	down	cytochrome_P450_71A1-like
MELO3C002314.2	2.883501703	0.00066187	up	cytochrome_P450_71A1-like
MELO3C002365.2	2.12419803	1.17E-18	up	Molybdenum_cofactor_sulfurase
MELO3C002374.2	4.074433792	0.0000194	up	11-beta-hydroxysteroid_dehydrogenase-like_protein
MELO3C002413.2	-5.070342143	0.001547714	down	Ankyrin_repeat_family_protein
MELO3C002420.2	2.018898501	0.0000702	up	Retrovirus-related_Pol_polyprotein_from_transposon_TNT_1-94
MELO3C002437.2	-1.452591653	0.000000156	down	p55
MELO3C002460.2	1.867715451	0.0000915	up	At3g20340
MELO3C002474.2	1.818774579	0.003057446	up	beta-glucosidase_47
MELO3C002501.2	3.540261778	0.0000402	up	cysteine-rich_receptor-like_protein_kinase_26_isoform_X1
MELO3C002513.2	-1.467432361	0.000126708	down	Phosphosulfolactate_synthase

MELO3C002514.2	1.880153214	0.0000675	up	BTB/POZ_domain-containing_protein_At3g22104
MELO3C002536.2	2.234457364	0.0000461	up	Serine/threonine-protein_kinase
MELO3C002553.2	-1.192070872	0.0000347	down	sugar_carrier_protein_C-like
MELO3C002575.2	-3.497679246	0.000000102	down	Hydroxyproline-rich_glycoprotein_family_protein
MELO3C002609.2	-1.023940687	0.003766249	down	serine/threonine-protein_kinase_HT1-like
MELO3C002630.2	-1.094048874	0.000448573	down	SNI1
MELO3C002641.2	-1.46707903	0.00000142	down	Protein_trichome_birefringence
MELO3C002694.2	2.611327721	3.06E-26	up	Trehalose-6-phosphate_synthase,_putative
MELO3C002727.2	-1.893111645	0.004193107	down	Chlorophyll_a-b_binding_protein,_chloroplastic
MELO3C002735.2	-1.052421003	0.001815496	down	Gibberellin_2-oxidase
MELO3C002747.2	-2.141847212	0.003156589	down	LRR_receptor-like_serine/threonine-
MELO3C002765.2	-11.99708053	4.26E-23	down	Myb/SANT-like_DNA-binding_domain_protein
MELO3C002875.2	2.792174882	0.0000539	up	WRKY_family_transcription_factor
MELO3C002917.2	3.501005462	0.000514063	up	Unknown_protein
MELO3C002945.2	-5.785027285	4.06E-16	down	Terpene_cyclase/mutase_family_member
MELO3C002976.2	2.531202683	0.000000556	up	transcription_termination_factor_MTERF5,_chloroplastic-like
MELO3C002980.2	1.065789215	3.42E-08	up	glutamate_5-kinase_isoform_X1
MELO3C003112.2	-1.049798334	0.001933172	down	Forkhead_box_protein_G1
MELO3C003155.2	-5.069111484	2.09E-12	down	aldo-keto_reductase_family_4_member_C9-like
MELO3C003164.2	-2.180408261	6.77E-08	down	Cysteine/Histidine-rich_C1_domain_family_protein,_putative
MELO3C003184.2	1.426263483	0.001192689	up	Transforming_growth_factor_beta_receptor_type_3
MELO3C003188.2	-3.522495588	0.000347873	down	glutathione_S-transferase_U8-like
MELO3C003195.2	-6.095308748	2.8E-14	down	17.5_kDa_class_I_heat_shock_protein
MELO3C003202.2	7.387115372	5.36E-08	up	beta-galactosidase
MELO3C003275.2	2.015567284	0.000000186	up	Peroxidase
MELO3C003279.2	-9.227543321	1.37E-13	down	Unknown_protein
MELO3C003304.2	7.325830763	2.84E-16	up	NF-E2_inducible_protein-like
MELO3C003331.2	-1.695433024	0.0025093	down	BAX_inhibitor-1
MELO3C003394.2	1.038456933	0.003523969	up	Protein_LSD1
MELO3C003401.2	-6.225290607	0.0000279	down	transcriptional_regulator_SUPERMAN-like
MELO3C003402.2	4.292430119	0.000396083	up	AT-hook_motif_nuclear-localized_protein_23
MELO3C003419.2	-1.700514746	0.002815772	down	Tetraspanin
MELO3C003426.2	-1.256651494	0.000514167	down	Inositol-tetrakisphosphate_1-kinase
MELO3C003441.2	-2.419046828	0.00000524	down	Xyloglucan_endotransglucosylase/hydrolase
MELO3C003467.2	1.04634427	6.63E-12	up	thioredoxin-related_transmembrane_protein_2
MELO3C003497.2	1.475773224	4.29E-10	up	Phosphoglycerate_mutase-like_protein_1
MELO3C003585.2	-3.170278508	0.000942104	down	Pentatricopeptide_repeat-containing_protein
MELO3C003598.2	1.324058282	0.000790453	up	Plant/F12B17-70_protein
MELO3C003620.2	-1.828959609	0.000118323	down	Sarcosine_oxidase_family_protein
MELO3C003720.2	7.86878028	1.11E-09	up	Exostosin_family_protein
MELO3C003731.2	-1.601079826	0.000119285	down	Protein_disulfide_isomerase_(PDI)-like_protein
MELO3C003732.2	1.438914351	0.004210638	up	Lactoylglutathione_lyase_/_glyoxalase_I_family_protei
MELO3C003735.2	2.823987447	1.24E-13	up	Alcohol_dehydrogenase,_putative
MELO3C003737.2	1.347423922	0.001490155	up	Subtilisin-like_protease
MELO3C003738.2	-1.064030814	0.002319744	down	KRR1_family_protein
MELO3C003770.2	1.733882728	0.0000576	up	TLD-domain_nucleolar_protein
MELO3C003781.2	1.158271326	0.0000757	up	Protein_SHI_RELATED_SEQUENCE_1
MELO3C003817.2	1.877688389	0.000000733	up	DNA_polymerase_epsilon_catalytic_subunit_A
MELO3C003821.2	2.1281238	0.000721315	up	agglutinin-like
MELO3C003823.2	-2.820840137	7.97E-08	down	jacalin-related_lectin_3-like
MELO3C003917.2	-5.793332327	3.09E-19	down	Heat_shock_70_kDa_protein
MELO3C004003.2	-1.397142893	0.00000303	down	La-related_protein_6_isoform_1
MELO3C004026.2	5.773346251	0.001798544	up	Auxin-responsive_protein
MELO3C004135.2	-4.934847323	0.000422815	down	leucine_aminopeptidase_2,_chloroplastic-like
MELO3C004137.2	1.63479704	1.26E-11	up	Purine_permease-related_family_protein
MELO3C004145.2	-1.340103006	0.001278014	down	transcription_factor_MYB86-like
MELO3C004181.2	2.243126285	0.0000305	up	homeobox_protein_knotted-1-like_1_isoform_X1
MELO3C004184.2	6.203518671	0.000000211	up	nicotianamine_synthase-like
MELO3C004193.2	-2.613242005	0.004448284	down	pyrophosphate-energized_vacuolar_membrane_proton_pump
MELO3C004214.2	-1.038368895	0.00330696	down	Chlorophyll_a-b_binding_protein,_chloroplastic
MELO3C004233.2	-1.053720921	0.001704643	down	psbP_domain-containing_protein_7,_chloroplastic_isoform_X1
MELO3C004242.2	3.998277761	0.0000937	up	Lipoxygenase
MELO3C004244.2	6.139279265	5.92E-29	up	Lipoxygenase
MELO3C004245.2	-4.003039289	0.000726313	down	Lipoxygenase
MELO3C004247.2	2.541492088	0.003168588	up	Lipoxygenase
MELO3C004249.2	9.013987283	2.79E-12	up	Lipoxygenase
MELO3C004250.2	3.826133681	0.0000923	up	Lipoxygenase
MELO3C004289.2	1.285196573	0.000496171	up	TMV_resistance_protein_N-like

MELO3C004313.2	-2.67857887	1.11E-15	down	TMV_resistance_protein_N-like
MELO3C004314.2	1.587660128	0.002259709	up	FANTASTIC_four-like_protein_(DUF3049)
MELO3C004385.2	-3.685843838	0.000000525	down	pathogenesis-related_protein_PR-4-like
MELO3C004401.2	4.996929541	0.000936041	up	protein_ACCELERATED_CELL_DEATH_6-like
MELO3C004507.2	-1.083388588	1.76E-08	down	DUF3119_family_protein
MELO3C004513.2	-1.307455018	0.003490065	down	protein_NRT1/_PTR_FAMILY_6.2
MELO3C004520.2	-1.477928973	0.002486457	down	protein_kinase_PINOID_2
MELO3C004541.2	1.261308995	0.000295572	up	glucan_endo-1,3-beta-glucosidase_8
MELO3C004551.2	-4.625401861	8.31E-30	down	ARF_guanine-nucleotide_exchange_factor_GNL2
MELO3C004556.2	-2.915490691	2.9E-42	down	heat_stress_transcription_factor_C-1-like
MELO3C004584.2	2.45405035	0.000000168	up	2-oxoglutarate_(2OG)_and_Fe(II)-dependent_oxygenase-like_protein
MELO3C004602.2	1.379235652	0.000882617	up	Leucine-rich_repeat_receptor-
MELO3C004624.2	1.511594247	0.000266977	up	Lysine-specific_histone_demethylase_1-like_protein
MELO3C004753.2	6.499114958	0.000000156	up	subtilisin-like_protease_SBT3.18
MELO3C004801.2	-5.1152111	8.27E-31	down	cytochrome_P450_71A1-like
MELO3C004825.2	5.274949408	3.64E-25	up	Ser/thr-rich_protein_T10_in_DGCR_region-
MELO3C004856.2	2.645815146	0.002801954	up	Protein_NDR1
MELO3C004914.2	6.173798818	1.24E-15	up	Cysteine_proteinase_inhibitor
MELO3C004941.2	-3.372013315	2.21E-08	down	Xyloglucan_endotransglucosylase/hydrolase
MELO3C004957.2	1.897906774	2.92E-09	up	ARM_repeat_superfamily_protein
MELO3C005186.2	10.78896221	2.26E-18	up	Germin-like_protein_1
MELO3C005190.2	-2.032186526	0.00000417	down	chitinase_10
MELO3C005193.2	-1.296964217	7.01E-10	down	Proline--tRNA_ligase
MELO3C005206.2	1.01941904	0.003007124	up	Glutaredoxin_domain-containing_protein/DEP_domain-
MELO3C005209.2	-1.060880353	0.0000251	down	Phytosulfokine_receptor,_putative
MELO3C005212.2	2.181527321	0.000000842	up	Unknown_protein
MELO3C005224.2	1.610462708	0.000139087	up	Glutamate_decarboxylase
MELO3C005237.2	1.638419748	0.00000318	up	Zinc_finger_family_protein
MELO3C005257.2	1.521586274	0.003712277	up	RNA-dependent_RNA_polymerase
MELO3C005263.2	1.031155749	0.000696497	up	Serine/threonine-protein_kinase
MELO3C005284.2	-1.930859687	0.000322035	down	RNA-dependent_RNA_polymerase
MELO3C005290.2	-1.627123274	0.003336131	down	Endoglucanase
MELO3C005310.2	1.873920712	1.96E-08	up	Ribose-5-phosphate_isomerase_A
MELO3C005315.2	-7.20015857	0.000875078	down	heavy_metal-
MELO3C005319.2	1.279431569	0.00000349	up	Calcium-binding_protein
MELO3C005356.2	1.682460462	0.001566573	up	Protein_CHUP1,_chloroplastic
MELO3C005375.2	1.421614445	0.000467199	up	Cytochrome_P450
MELO3C005393.2	1.780028818	0.002030111	up	MADS-box_protein_AGL42-like
MELO3C005442.2	2.570649837	0.001386185	up	BnaC09g40830D_protein
MELO3C005444.2	1.371054951	0.003080138	up	Glutamate_dehydrogenase
MELO3C005476.2	1.495245141	0.0000208	up	Auxin-responsive_protein
MELO3C005487.2	2.591500556	0.00000671	up	protein_NRT1/_PTR_FAMILY_5.10-like
MELO3C005490.2	-2.224479468	1.51E-09	down	U-box_domain-containing_protein_35-
MELO3C005532.2	-1.291216542	0.002067271	down	Pectinesterase
MELO3C005540.2	-1.046997166	0.00136507	down	14_kDa_proline-rich_protein_dc2.15
MELO3C005571.2	-1.825492661	0.000496178	down	Cytochrome_P450_family_protein
MELO3C005574.2	-1.096991488	0.00169267	down	Cytochrome_P450,_putative
MELO3C005576.2	1.096635715	0.00000127	up	Cytochrome_P450
MELO3C005611.2	-1.605428696	0.00124167	down	transcription_factor_bHLH120-like
MELO3C005624.2	1.060242213	0.0000523	up	SPX_domain-containing_membrane_protein
MELO3C005628.2	1.327785494	0.00000112	up	UAA_transporter
MELO3C005656.2	3.722071656	0.001255436	up	Protein_LURP-one-related_11
MELO3C005657.2	1.95223265	0.000108912	up	Protein_LURP-one-related_11
MELO3C005736.2	-2.223794537	1.14E-09	down	Cytoplasmic_tRNA_2-thiolation_protein
MELO3C005737.2	-1.473034186	0.000187739	down	Kinase_family_protein
MELO3C005751.2	2.057759522	0.000261064	up	Trehalase
MELO3C005752.2	1.871599131	0.004155458	up	Short-chain_dehydrogenase_TIC_32,_chloroplastic
MELO3C005803.2	1.793077937	0.000837409	up	WAT1-related_protein
MELO3C005805.2	2.706327166	0.002865033	up	heavy_metal-associated_isoprenylated_plant_protein_21-like
MELO3C005832.2	-2.40930042	0.00000223	down	cucumisin-like_isoform_X1
MELO3C005835.2	-1.197592961	0.00140632	down	Keratin-associated_protein_(DUF819)
MELO3C005877.2	1.201537892	0.000140035	up	Spermidine_synthase
MELO3C005883.2	-1.594180828	0.000000976	down	Zinc_finger_protein_CONSTANS
MELO3C005923.2	-5.105558872	5.92E-12	down	Chloroplast_small_heat_shock_protein
MELO3C005947.2	2.063249407	1.6E-12	up	basic_blue_protein
MELO3C005994.2	-1.192159227	0.000272276	down	cytochrome_b5-like
MELO3C006047.2	-1.923299583	0.00000663	down	C2_domain-containing_protein
MELO3C006138.2	-1.862219374	0.000653387	down	BON1-associated_protein_2
MELO3C006172.2	-1.083941613	0.000229641	down	Cytochrome_P450,_putative

MELO3C006208.2	2.281456634	0.000622854	up	Pectinesterase
MELO3C006222.2	1.383257648	0.000406162	up	Calcium-binding_EF-hand
MELO3C006237.2	-2.126004122	0.000016	down	Cytochrome_P450_family_ent-kaurenoic_acid_oxidase
MELO3C006239.2	-1.060513364	0.0000268	down	Receptor_protein_kinase,_putative
MELO3C006268.2	-1.306587905	0.000000116	down	10_kDa_chaperonin_isoform_X1
MELO3C006288.2	1.462904262	2.25E-15	up	Heat_Stress_Transcription_Factor_family_protein
MELO3C006353.2	-2.80225956	0.002139775	down	Glutathione_S-transferase
MELO3C006355.2	-1.624702957	0.00000166	down	Glutathione_s-transferase,_putative
MELO3C006362.2	-1.618738989	0.000017	down	Beta-amylase
MELO3C006365.2	-1.103817748	0.003068064	down	FBT8
MELO3C006371.2	-1.245931018	0.003770351	down	Auxin-responsive_protein
MELO3C006420.2	1.408442868	0.0000294	up	Cotton_fiber_protein
MELO3C006431.2	3.746637388	0.00000458	up	ethylene-responsive_transcription_factor_ERF098-like
MELO3C006438.2	-1.87169226	0.00000165	down	1-aminocyclopropane-1-carboxylate_oxidase_homolog_1-like
MELO3C006456.2	4.160048314	8.47E-15	up	Protease_inhibitor_protein
MELO3C006467.2	2.933533066	0.001397867	up	Alba_DNA/RNA-binding_protein
MELO3C006501.2	-3.004147627	0.0000001	down	heat_stress_transcription_factor_A-6b
MELO3C006504.2	1.685041838	0.00112657	up	Protein_IQ-DOMAIN_31
MELO3C006511.2	1.183394009	0.0000274	up	serine/threonine-protein_kinase_STY8-like
MELO3C006536.2	-3.169084162	2.91E-16	down	Heat_shock_70_kDa_protein
MELO3C006537.2	-1.655527085	0.000130636	down	Serine/threonine-protein_kinase
MELO3C006539.2	-1.070204946	0.001831373	down	photosystem_I_reaction_center_subunit_II,_chloroplast
MELO3C006543.2	2.659629341	0.00000352	up	sugar_transport_protein_13-like
MELO3C006546.2	1.489645693	0.000000191	up	BRI1_kinase_inhibitor_1-like
MELO3C006552.2	-1.547356771	0.00000343	down	Glucose-1-phosphate_adenylyltransferase
MELO3C006558.2	1.421947885	0.000000254	up	Stem-specific_protein_TSJT1
MELO3C006567.2	1.159863698	0.000491349	up	EF-hand_calcium-binding_domain-
MELO3C006588.2	5.764911554	0.000161859	up	DUF679_domain_membrane_protein_2
MELO3C006603.2	-2.267415768	4.63E-08	down	vesicle-associated_protein_2-2-like_isoform_X1
MELO3C006689.2	1.029488048	0.001236998	up	Chaperone_protein_dnaJ_49
MELO3C006696.2	1.250250581	0.0000168	up	GATA_transcription_factor_16-like
MELO3C006712.2	1.593420363	0.003774812	up	UPF0481_protein_At3g47200
MELO3C006755.2	-1.251667125	0.000000173	down	Diacylglycerol_acyltransferase
MELO3C006773.2	1.597797628	0.000210468	up	Rapid_ALkalinization_Factor
MELO3C006783.2	-1.603538224	0.00000125	down	Peptidylprolyl_isomerase
MELO3C006802.2	-4.839305237	4.92E-34	down	Protein_DEHYDRATION-INDUCED_19
MELO3C006865.2	2.336579066	1.06E-14	up	sorbitol_dehydrogenase-like
MELO3C006888.2	3.052368296	0.00000014	up	TERMINAL_FLOWER_1-like_protein
MELO3C006898.2	-1.232305976	0.000198068	down	Methyl-CpG-binding_domain-containing_13-
MELO3C006933.2	-2.40989815	0.000758931	down	NADH-
MELO3C006944.2	-2.097873567	0.000055	down	ferric_reduction_oxidase_4-like
MELO3C006965.2	-3.287788958	3.86E-09	down	Acidic_endochitinase
MELO3C006973.2	-1.263589505	0.002509195	down	ethylene-responsive_transcription_factor_CRF4-like
MELO3C007022.2	1.009328906	0.003132643	up	UDP-N-acetylglucosamine_diphosphorylase_2-like
MELO3C007059.2	-1.268414972	0.000178917	down	Fatty_acid_2-hydroxylase
MELO3C007070.2	1.498921867	0.00000183	up	Kinase,_putative
MELO3C007125.2	-1.643488385	0.003029182	down	Protein_TIFY_5A
MELO3C007152.2	1.478867328	0.0000542	up	Leucine-rich_receptor-
MELO3C007173.2	-1.922412328	0.000938078	down	ABC_transporter_B_family_protein
MELO3C007232.2	1.120011138	0.000264322	up	spermidine_coumaroyl-CoA_acyltransferase
MELO3C007255.2	-6.289932007	0.000343157	down	NAC_domain-containing_protein
MELO3C007261.2	-1.062784863	0.003408155	down	dnaJ_protein_homolog_1-like
MELO3C007279.2	-1.983174317	0.001418608	down	Pollen_Ole_e_1_allergen_and_extensin_family_protein
MELO3C007306.2	-1.86306543	0.002470335	down	Glucose-fructose_oxidoreductase_domain-containing_protein_2,_putative
MELO3C007315.2	-2.647347397	0.000267897	down	Internal_alternative_NAD(P)H-ubiquinone_oxidoreductase_A1,_mitochondrial
MELO3C007317.2	-1.466290201	0.00085012	down	Transmembrane_protein,_putative
MELO3C007318.2	-2.925295214	4.88E-12	down	Transmembrane_protein,_putative
MELO3C007337.2	1.847169956	0.000300716	up	Zinc_finger,_B-box
MELO3C007367.2	1.24976772	0.001406041	up	Receptor-like_kinase
MELO3C007391.2	2.064079085	0.00043235	up	Glycosyl_transferase,_family_31
MELO3C007409.2	-1.022628473	0.001289092	down	WRKY_family_transcription_factor
MELO3C007410.2	1.147486512	0.001827805	up	wall-associated_receptor_kinase_2
MELO3C007425.2	1.583143239	0.0000251	up	1-aminocyclopropane-1-carboxylate_oxidase_1
MELO3C007430.2	1.076445068	0.003608484	up	DUF506_family_protein_(DUF506)
MELO3C007464.2	-2.086349376	8.99E-19	down	tRNA(His)_guanylyltransferase_2-like
MELO3C007470.2	3.120484827	4.28E-09	up	WRKY_family_transcription_factor
MELO3C007472.2	2.305224605	1.53E-09	up	LRR_receptor-like_serine/threonine-protein_kinase_GSO1_isoform_X1
MELO3C007480.2	1.777087599	0.001078063	up	Cytochrome_P450_family_protein

MELO3C007482.2	-2.626380567	2.8E-09	down	Cytochrome_P450_family_protein
MELO3C007509.2	1.073149466	0.0000597	up	BTB/POZ_domain-containing_protein
MELO3C007527.2	-1.325934406	0.002309352	down	dof_zinc_finger_protein_DOF3.4
MELO3C007595.2	4.590373802	0.001958085	up	zinc_finger_protein_7-like
MELO3C007630.2	1.032741446	0.002114158	up	protein_SHORT-ROOT
MELO3C007638.2	-1.018283368	0.0000605	down	Pentatricopeptide_repeat-containing_protein
MELO3C007658.2	-1.199053906	0.00000207	down	inactive_LRR_receptor-like_serine/threonine-protein_kinase_BIR2
MELO3C007663.2	-3.443777531	0.00000128	down	transcription_factor_RAX2
MELO3C007687.2	3.685445421	1.08E-14	up	Phosphoenolpyruvate_carboxykinase
MELO3C007702.2	-2.004711323	0.000406571	down	Alcohol_dehydrogenase,_putative
MELO3C007731.2	-1.74026458	6.06E-09	down	Protein_DETOXIFICATION
MELO3C007773.2	-1.362516484	0.0000238	down	TSA:_Wollemia_nobilis_Ref_Wollemi_Transcript_29023_1096_transcribed_RNA_sequence
MELO3C007784.2	1.900465762	0.000815836	up	Unknown_protein
MELO3C007799.2	-1.615438159	0.004394206	down	cytochrome_P450_CYP736A12-like
MELO3C007820.2	-1.118196557	0.002112493	down	Anti-Muellerian_hormone_type-2_receptor
MELO3C007832.2	1.45322511	0.000294948	up	zinc-finger_homeodomain_protein_2
MELO3C007854.2	-1.49830043	0.00000406	down	TIM-barrel_signal_transduction_protein_isoform_1
MELO3C007861.2	2.274878206	0.000960633	up	Aaa-atpase
MELO3C007874.2	1.78104587	1.65E-15	up	Purple_acid_phosphatase
MELO3C007877.2	4.853897207	7.59E-31	up	Protein_SHI_RELATED_SEQUENCE_1
MELO3C007905.2	1.008522011	0.00069394	up	transcription_factor_bHLH121
MELO3C007913.2	1.275659393	0.003365059	up	Thaumatin-like_protein_1
MELO3C007928.2	-2.867873545	0.0000428	down	GDSL_esterase/lipase_EXL3-like
MELO3C007937.2	-1.672764046	0.000379983	down	aspartyl_protease_family_protein_1-like
MELO3C007949.2	-1.984677239	0.0000222	down	thiosulfate_sulfurtransferase_16,_chloroplastic
MELO3C007961.2	-4.126484293	0.000000118	down	Chitinase
MELO3C007962.2	-3.89647574	0.000000535	down	Chitinase
MELO3C007986.2	-1.469882161	3.29E-08	down	Histidine_phosphatase_family_(Branch_1)_protein
MELO3C008025.2	1.892714037	0.00000254	up	WAT1-related_protein
MELO3C008064.2	-1.176947523	3.1E-09	down	Histone_deacetylase,_putative
MELO3C008073.2	1.047526773	0.0000262	up	Fanconi_anemia_group_M_protein_isoform_X1
MELO3C008075.2	1.187711148	0.002952797	up	12-oxophytodienoate_reductase-like_protein
MELO3C008088.2	-1.153460946	2.45E-12	down	phospholipase_SGR2
MELO3C008124.2	-1.585230504	2.72E-12	down	SNARE_associated_Golgi_protein_family
MELO3C008176.2	3.041070784	0.001245123	up	Unknown_protein
MELO3C008196.2	5.609410818	0.001548351	up	Chloride_channel_protein
MELO3C008203.2	4.293782226	4.89E-10	up	protein_LYK2
MELO3C008214.2	-1.843298717	9.75E-08	down	Emb|CAB62340.1
MELO3C008226.2	1.192222754	0.002423586	up	E3_ubiquitin_protein_ligase_DRIP2-like
MELO3C008229.2	1.89048328	0.002379537	up	Thaumatin-like_protein
MELO3C008293.2	-1.129491885	0.002822039	down	Receptor-kinase,_putative
MELO3C008350.2	1.3555197	0.00000952	up	Vacuolar_iron_transporter-like_protein
MELO3C008375.2	2.321752625	0.000309432	up	Unknown_protein
MELO3C008378.2	6.738124714	0.00000335	up	Unknown_protein
MELO3C008442.2	-10.00172231	4.96E-13	down	wall-associated_receptor_kinase_2-like
MELO3C008464.2	-1.900877546	2.75E-29	down	transcription_initiation_factor_TFIID_subunit_11-like
MELO3C008673.2	-1.626136208	6.85E-09	down	UDP-N-acetylmuramoyl-L-alanyl-D-glutamate--2,_6-diaminopimelate_ligase
MELO3C008836.2	1.301627345	0.004033138	up	plant_cysteine_oxidase_2-like
MELO3C008882.2	-4.054360203	5.23E-08	down	serine/threonine-protein_kinase_SRK2B-
MELO3C008885.2	-1.190973543	0.002961762	down	Serine/threonine_protein_kinase
MELO3C008899.2	1.435943126	0.000000189	up	Kinase_family_protein
MELO3C008938.2	1.142336828	0.000209661	up	lipase-like_PAD4
MELO3C008953.2	6.289776741	0.0000197	up	calmodulin-binding_protein_60_A-like
MELO3C009010.2	1.071975664	0.000000307	up	serine/threonine-protein_kinase_RHS3-like
MELO3C009113.2	-3.327934536	0.0000968	down	transcription_repressor_OFP2-like
MELO3C009122.2	-2.233011016	0.00011489	down	Short-chain_dehydrogenase/reductase
MELO3C009141.2	-1.643364274	7.33E-09	down	PEBP_(Phosphatidylethanolamine-binding_protein)_family_protein
MELO3C009169.2	-2.428038595	0.000650408	down	GDSL_esterase/lipase
MELO3C009190.2	-1.391094133	0.003692484	down	LOW_QUALITY_PROTEIN:_protein_NRT1/_PTR_FAMILY_8.1
MELO3C009212.2	1.43821185	0.000000754	up	G_patch_domain_protein
MELO3C009218.2	1.075991933	0.000310019	up	protein_NRT1/_PTR_FAMILY_5.6
MELO3C009232.2	5.064475255	0.0000444	up	tropinone_reductase_homolog
MELO3C009238.2	3.712730924	1.19E-09	up	Lectin_receptor_kinase
MELO3C009263.2	-1.182650047	0.000384133	down	E3_ubiquitin-protein_ligase_XB3-like
MELO3C009284.2	-1.212740003	1.03E-09	down	Protein_SLOW_GREEN_1,_chloroplastic
MELO3C009332.2	1.429731685	0.0000576	up	protein_indeterminate-domain_9
MELO3C009389.2	-1.994877099	0.000142012	down	Glycosyltransferase

MELO3C009390.2	-1.648039658	0.000000284	down	Glycosyltransferase
MELO3C009391.2	-2.201890043	3.22E-08	down	Glycosyltransferase
MELO3C009404.2	-2.845138753	0.0000994	down	1-aminocyclopropane-1-
MELO3C009416.2	2.303399354	0.00012073	up	protein_CHUP1,_chloroplastic-like
MELO3C009422.2	1.373122354	0.0000394	up	Myb/SANT-like_DNA-binding_domain_protein
MELO3C009427.2	-2.662583773	7.42E-08	down	AT3g10020/T22K18_16
MELO3C009437.2	1.797800081	0.004228465	up	DUF868_family_protein,_putative_(DUF868)
MELO3C009441.2	1.238794908	0.0000299	up	ethylene-responsive_transcription_factor_ERF024-like
MELO3C009452.2	-1.122876532	0.000863643	down	Drug_resistance_transporter-
MELO3C009460.2	-2.479497355	0.003280289	down	Unknown_protein
MELO3C009461.2	1.290410123	0.004078199	up	Protein_LITTLE_ZIPPER_4
MELO3C009476.2	3.124297052	6.37E-15	up	Alpha_carbonic_anhydrase
MELO3C009492.2	-1.143716248	0.00074742	down	60S_ribosomal_protein_L12
MELO3C009501.2	-1.154740833	0.0000116	down	lysosomal_Pro-X_carboxypeptidase-like
MELO3C009506.2	-9.944593699	1.98E-15	down	secoisolariciresinol_dehydrogenase-like
MELO3C009529.2	-1.318477311	0.000408088	down	YLS9
MELO3C009551.2	-1.829264784	0.000223043	down	nuclear_transcription_factor_Y_subunit_A-10
MELO3C009569.2	1.481654967	0.001781101	up	Gb|AAF02136.1
MELO3C009630.2	1.641450706	0.000000937	up	Fatty_acid_desaturase
MELO3C009637.2	-3.85484365	0.001035466	down	At3g57950
MELO3C009641.2	-2.493853777	0.003694163	down	protein_STRUBBELIG-RECEPTOR_FAMILY_2_isoform_X1
MELO3C009674.2	-2.732247659	2.49E-29	down	Beta-glucosidase,_putative
MELO3C009681.2	1.010552643	0.001037313	up	At3g57450
MELO3C009686.2	1.133285196	0.003492202	up	Pleiotropic_drug_resistance_ABC_transporter
MELO3C009696.2	1.261575142	3.3E-12	up	Pentatricopeptide_repeat-containing_family_protein
MELO3C009715.2	1.779050042	0.000266294	up	LOW_QUALITY_PROTEIN:_kinesin-3-like
MELO3C009728.2	-1.593969119	0.0000463	down	Calmodulin-binding_protein_25
MELO3C009737.2	2.524183505	0.004098475	up	Cytochrome_P450,_putative
MELO3C009755.2	-1.225249813	5.84E-12	down	Sigma_factor_binding_protein_1,_chloroplastic
MELO3C009757.2	1.426729553	0.003061816	up	Amino_acid_transporter_family_protein
MELO3C009763.2	-1.252692042	0.00000978	down	centromere-associated_protein_E
MELO3C009811.2	-3.872552917	0.001070326	down	Inactive_purple_acid_phosphatase
MELO3C009870.2	1.071133321	0.00000996	up	aquaporin_NIP2-1-like
MELO3C009871.2	1.016500929	0.000420831	up	aquaporin_NIP2-1-like
MELO3C009900.2	12.84883473	9.17E-27	up	AAA-ATPase_ASD,_mitochondrial-like
MELO3C009902.2	3.715781232	3E-14	up	AAA-ATPase_ASD,_mitochondrial-like
MELO3C010017.2	6.402906923	0.0000828	up	polygalacturonase
MELO3C010098.2	8.027745422	4.69E-10	up	Unknown_protein
MELO3C010155.2	8.241899454	3.64E-10	up	Unknown_protein
MELO3C010216.2	-2.110209517	4.02E-20	down	APO_protein_2,_chloroplastic
MELO3C010244.2	-1.444261965	0.000195309	down	Temperature-induced_lipocalin
MELO3C010249.2	1.475236203	1.6E-10	up	chitotriosidase-1-like
MELO3C010276.2	-1.741011213	0.000683974	down	At1g15760
MELO3C010312.2	-1.804238285	0.000395101	down	36.4_kDa_proline-rich_protein
MELO3C010317.2	1.859157292	0.0000275	up	Auxin-responsive_protein
MELO3C010318.2	-3.613430756	0.000400268	down	Clathrin_assembly_protein,_putative
MELO3C010367.2	1.311886751	0.000272203	up	Esterase_PIR7B,_putative
MELO3C010444.2	1.910884729	0.000103724	up	Nitrate_reductase
MELO3C010481.2	-1.311217892	0.0000294	down	Sister_chromatid_cohesion_PDS5-B-B-like_protein
MELO3C010491.2	-5.982173739	0.000608747	down	Unknown_protein
MELO3C010504.2	-3.206384992	0.000625408	down	At1g77400
MELO3C010561.2	-2.101796199	0.000013	down	Seed_maturation_protein_PM28
MELO3C010587.2	-1.706148107	3.85E-16	down	Transmembrane_protein,_putative
MELO3C010588.2	-1.126698843	0.000000701	down	Transmembrane_protein,_putative
MELO3C010697.2	1.446557815	0.003654132	up	SufE-like_protein,_chloroplastic
MELO3C010719.2	-6.011371087	0.000411831	down	vinorine_synthase
MELO3C010762.2	1.634445889	8.01E-20	up	Ferrochelatase
MELO3C010767.2	-4.154021697	0.001354009	down	Unknown_protein
MELO3C010817.2	-1.018554714	0.000616589	down	Subtilisin-like_protease
MELO3C010822.2	-1.807353549	0.000000323	down	Gibberellin-regulated_protein_1
MELO3C010825.2	-4.90739808	2.69E-21	down	Receptor-kinase,_putative
MELO3C010826.2	-3.415805462	0.000151656	down	Receptor-kinase,_putative
MELO3C010864.2	3.432697584	0.0000875	up	Calcium-binding_EF-hand_family_protein,_putative
MELO3C010978.2	-1.048170149	0.001070646	down	purple_acid_phosphatase-like
MELO3C010980.2	-3.318868627	0.00339458	down	glutamate_receptor_2.9-like
MELO3C010984.2	3.176595861	0.001261803	up	Protein_SHI_RELATED_SEQUENCE_1
MELO3C011008.2	-2.738624178	0.000415106	down	Gibberellin-regulated_protein_2
MELO3C011028.2	1.082040987	5.98E-12	up	14-3-3-like_protein
MELO3C011158.2	-3.721952361	0.000110304	down	Flavin-containing_monooxygenase
MELO3C011171.2	-1.768990995	1.01E-12	down	AIG2-like_protein_D
MELO3C011229.2	1.623468716	0.0000412	up	EG45-like_domain_containing_protein

MELO3C011232.2	-2.32541825	1.77E-18	down	Boron_transporter-like_protein
MELO3C011248.2	2.065273014	0.0000169	up	UPF0481_protein_At3g47200
MELO3C011252.2	5.532485672	0.00000436	up	EG45-like_domain_containing_protein
MELO3C011270.2	1.871505921	0.000264766	up	Adenine_phosphoribosyltransferase,_putative
MELO3C011298.2	-1.784266504	0.002818659	down	Eukaryotic_translation_initiation_factor_5A
MELO3C011299.2	2.124550797	0.001570602	up	BTB/POZ_domain-containing_protein_NPY1
MELO3C011302.2	-2.037288103	0.001392438	down	early_nodulin-like_protein_1
MELO3C011304.2	1.200301456	0.000876201	up	Histone-lysine_N-methyltransferase_SUV420H14-20,_putative
MELO3C011319.2	2.067971775	0.00000117	up	Glutamine_dumper,_putative
MELO3C011369.2	1.073343474	0.003274475	up	Hexosyltransferase
MELO3C011405.2	3.788340202	5.03E-10	up	Unknown_protein
MELO3C011440.2	-1.968359985	2.86E-10	down	2-alkenal_reductase_(NADP(+)-dependent)-like
MELO3C011443.2	-4.223742321	1.92E-23	down	Glycosyltransferase
MELO3C011472.2	1.639028032	0.001665862	up	Ankyrin_repeat-containing_protein
MELO3C011474.2	2.07182104	0.000160969	up	Ankyrin_repeat_family_protein
MELO3C011475.2	4.962576789	1.88E-38	up	Ankyrin_repeat_family_protein
MELO3C011476.2	6.934202253	0.00000014	up	Ankyrin_repeat_family_protein
MELO3C011535.2	2.006078045	0.000000376	up	Transmembrane_protein,_putative
MELO3C011553.2	-1.000404344	0.000101935	down	IST1-like_protein
MELO3C011576.2	-2.066231187	0.0000148	down	zinc_finger_protein_CONSTANS-LIKE_6
MELO3C011587.2	-1.390351292	8.7E-13	down	Histone-lysine_N-methyltransferase_SUV420H14-20,_putative
MELO3C011744.2	-4.549652928	0.00000113	down	ferric_reduction_oxidase_2-like
MELO3C011796.2	1.040736672	0.0000605	up	type_I_inositol_polyphosphate_5-phosphatase_12_isoform_X2
MELO3C011801.2	8.177940102	0.000000233	up	WAT1-related_protein_At5g07050
MELO3C011815.2	-3.507510649	0.001181245	down	ABC_transporter_B_family_member_4-like
MELO3C011854.2	1.674681009	4.27E-18	up	Cytochrome_oxidase_assembly_3,_mitochondrial
MELO3C011872.2	8.101907093	1.92E-81	up	Caffeoyl-CoA_O-methyltransferase
MELO3C011880.2	-4.745232041	0.00333239	down	Proteasome_subunit_beta_type
MELO3C011883.2	-1.901164916	0.000798608	down	OTU_domain-containing_protein
MELO3C011884.2	1.094259995	1.89E-08	up	urease
MELO3C011901.2	1.153100307	0.00204633	up	Short-chain_dehydrogenase/reductase_family_protein
MELO3C011908.2	-2.407620223	3.95E-10	down	Heat_shock_protein_HSP26
MELO3C011911.2	-1.1769921	0.002194668	down	photosystem_II_core_complex_proteins_psbY,_chloroplastic-like
MELO3C011917.2	3.395344412	0.001509199	up	protein_terminal_ear1_homolog
MELO3C011928.2	-2.492512953	0.00000213	down	cytochrome_P450_71B19-like
MELO3C011968.2	-2.705370191	0.000521652	down	Lipase
MELO3C011971.2	1.83193469	0.000180152	up	Heme-binding_protein_2
MELO3C011980.2	-6.423333678	2.09E-11	down	F-box/kelch_protein
MELO3C011991.2	-6.599905693	2.4E-18	down	Hexosyltransferase
MELO3C011994.2	1.502369356	0.004141569	up	BHLH_transcription_factor
MELO3C012009.2	-1.023728916	0.0000736	down	Potassium_transporter
MELO3C012015.2	-1.458289122	2.6E-09	down	WAT1-related_protein
MELO3C012016.2	1.365426921	0.000279861	up	MLP-like_protein_423
MELO3C012045.2	1.056507985	0.000000342	up	Protein_ENHANCED_DISEASE_RESISTANCE_2
MELO3C012055.2	-3.806801311	0.00385662	down	NRT1/PTR_family_protein_2.2
MELO3C012065.2	1.233975112	0.00010471	up	protein_NRT1/_PTR_FAMILY_4.6
MELO3C012085.2	-2.235167046	0.002271809	down	HVA22-like_protein
MELO3C012114.2	-1.924945274	4.19E-12	down	NAC_domain-containing_protein,_putative
MELO3C012122.2	-2.738284849	0.000907553	down	phosphate_transporter_PHO1_homolog_3-like
MELO3C012127.2	1.565583533	0.002108705	up	protein_argonaute_7
MELO3C012162.2	1.257434471	0.000495004	up	BnaC07g22510D_protein
MELO3C012181.2	2.729138102	0.001824636	up	Basic-
MELO3C012189.2	-1.250122679	0.0000629	down	DUF1262_family_protein
MELO3C012191.2	-1.216948783	0.00092525	down	DUF1262_family_protein_(DUF1262)
MELO3C012222.2	2.514164821	0.000202323	up	Lysine_histidine_transporter
MELO3C012263.2	-2.377376851	0.001261328	down	zinc_finger_protein_6
MELO3C012265.2	1.254880643	0.000249412	up	Zinc_finger_protein,_putative
MELO3C012274.2	2.597614314	0.0000342	up	Unknown_protein
MELO3C012291.2	-1.051434767	0.000351292	down	Protein_DETOXIFICATION
MELO3C012296.2	-4.149900407	0.000692609	down	Unknown_protein
MELO3C012324.2	5.009956815	1.64E-28	up	3-oxo-5-alpha-steroid_4-dehydrogenase_1-like
MELO3C012328.2	-1.31617163	0.004051948	down	Nuclear_pore_complex_protein_NUP1
MELO3C012352.2	-2.038771331	0.000147341	down	germin-like_protein_subfamily_3_member_2
MELO3C012357.2	1.617801331	3.95E-10	up	Diphosphomevalonate_decarboxylase
MELO3C012394.2	1.366287065	0.000477489	up	Pectate_lyase
MELO3C012395.2	-2.558881323	0.00000719	down	Protein_nuclear_fusion_defective_4
MELO3C012407.2	-1.119155259	0.00000201	down	SKP1-like_protein_12
MELO3C012409.2	-1.455474767	0.0000114	down	Amino_acid_transporter_family_protein

MELO3C012468.2	7.471380266	1.54E-08	up	Annexin
MELO3C012498.2	2.457888639	0.00042433	up	protein_RADIALIS-like_3
MELO3C012550.2	-3.52213516	0.0000208	down	polyol_transporter_5-like
MELO3C012551.2	4.866958273	0.000106333	up	polyol_transporter_5-like
MELO3C012573.2	10.5296176	1.06E-17	up	NAC_domain-containing_protein,_putative
MELO3C012574.2	10.89987778	2.12E-19	up	Retrovirus-related_Pol_polyprotein_from_transposon_TNT_1-94
MELO3C012626.2	-1.73856673	0.00017105	down	Unknown_protein
MELO3C012644.2	6.340407261	0.0000236	up	Unknown_protein
MELO3C012683.2	-1.309569218	3.65E-08	down	Pentatricopeptide_repeat-containing_protein,_mitochondrial
MELO3C012686.2	1.903140884	5.63E-10	up	protein_TPX2-like
MELO3C012712.2	-1.77574317	0.002104951	down	NAD(P)H-quinone_oxidoreductase_subunit_M,_chloroplastic
MELO3C012753.2	8.113239977	3.87E-10	up	Unknown_protein
MELO3C012911.2	1.268754803	0.00000826	up	S-adenosylmethionine_synthase
MELO3C012912.2	-2.408390243	0.00000014	down	Chlorophyll_a-b_binding_protein,_chloroplastic
MELO3C012919.2	2.501815843	0.00000465	up	GDSL_esterase/lipase_At3g62280
MELO3C012921.2	-1.598383248	4.39E-13	down	GDSL_esterase/lipase
MELO3C012964.2	-1.584572379	0.003436493	down	Unknown_protein
MELO3C012965.2	-2.036812583	8.91E-09	down	Mitochondrial_carrier_protein
MELO3C012973.2	1.116924813	0.003477484	up	Homeobox_leucine_zipper_protein
MELO3C012987.2	-2.039172628	0.00145112	down	Avr9/Cf-9_rapidly_elicited_protein
MELO3C013003.2	1.949136709	0.00000595	up	Auxin-responsive_protein
MELO3C013009.2	-2.594917077	3.01E-08	down	ATP-dependent_6-phosphofructokinase_6-like
MELO3C013014.2	-2.000314564	0.000320215	down	Inositol_oxygenase
MELO3C013021.2	-1.842618689	0.00128335	down	Receptor_protein_kinase,_putative
MELO3C013043.2	1.079269904	0.001742721	up	Dof_zinc_finger_protein
MELO3C013090.2	7.171005376	9.42E-08	up	Unknown_protein
MELO3C013159.2	1.420326612	0.000229842	up	casein_kinase_II_subunit_beta-2
MELO3C013175.2	1.152357131	0.000101678	up	Pentatricopeptide_repeat-containing_family_protein
MELO3C013246.2	2.093107523	0.00000255	up	Glutamate_receptor
MELO3C013292.2	6.252883518	0.002241311	up	expansin-like_A2
MELO3C013342.2	3.263455792	3.01E-18	up	FAD-dependent_urate_hydroxylase-like
MELO3C013343.2	1.237450345	1.06E-11	up	FAD-dependent_urate_hydroxylase-like
MELO3C013365.2	-11.81200608	5.53E-23	down	Unknown_protein
MELO3C013367.2	-1.294103687	0.00000736	down	Auxin_influx_transporter
MELO3C013392.2	1.642070452	0.00000994	up	Retrovirus-related_Pol_polyprotein_from_transposon_TNT_1-94
MELO3C013403.2	-2.057446757	0.0000342	down	auxin-responsive_protein_SAUR36-like
MELO3C013449.2	-2.122186118	0.00000666	down	Isoflavone_reductase_like
MELO3C013562.2	-3.017030151	2.58E-12	down	Flavin-containing_monooxygenase
MELO3C013570.2	1.695387282	0.000180352	up	Protein_LIGHT-DEPENDENT_SHORT_HYPOCOTYLS_10
MELO3C013600.2	-1.653054388	0.001444178	down	O-methyltransferase,_putative
MELO3C013710.2	-1.700690364	0.000757009	down	Auxin_efflux_carrier
MELO3C013735.2	1.436445564	0.000199474	up	CoA_ligase
MELO3C013770.2	-3.73331786	0.000428289	down	Endoglucanase
MELO3C013790.2	-1.276096428	0.000128655	down	urea-proton_symporter_DUR3
MELO3C013844.2	1.54775519	0.000000277	up	Tetraspanin_family_protein
MELO3C013868.2	-2.739566465	3.86E-16	down	Cytochrome_P450_family_ent-kaurenoic_acid_oxidase
MELO3C013886.2	1.062891861	0.001531986	up	methyltransferase-like_protein_13
MELO3C013916.2	-1.648779776	0.000197856	down	ethylene-responsive_transcription_factor_2
MELO3C013945.2	-2.02699374	0.000118602	down	Small_heat_shock_protein,_chloroplastic
MELO3C013946.2	-2.370799911	0.000000014	down	Small_heat_shock_protein,_chloroplastic
MELO3C013956.2	-5.989173098	0.000000231	down	14_kDa_proline-rich_protein_DC2.15
MELO3C013960.2	1.454521576	5.98E-08	up	protein_NRT1/_PTR_FAMILY_6.3-like
MELO3C013969.2	-1.666032713	0.002810824	down	Trehalose_6-phosphate_phosphatase
MELO3C014047.2	-2.40426155	8.12E-13	down	(-)-germacrene_D_synthase-like
MELO3C014062.2	-1.423613412	7.22E-09	down	Tir-nbs_resistance_protein
MELO3C014076.2	6.433668564	0.000000244	up	subtilisin-like_protease_SBT5.3
MELO3C014093.2	-7.107210023	0.00000331	down	Unknown_protein
MELO3C014117.2	-1.177342791	0.003449635	down	Kinase,_putative
MELO3C014158.2	-1.991537474	0.004322628	down	Expansin-A9
MELO3C014159.2	-2.828356721	0.001182195	down	Unknown_protein
MELO3C014214.2	1.661165139	0.000000143	up	ABC_transporter_G_family-like_protein
MELO3C014223.2	1.420696854	0.000697044	up	phenylalanine_ammonia-lyase-like
MELO3C014224.2	-1.308202402	0.000119216	down	phenylalanine_ammonia-lyase-like
MELO3C014235.2	2.006082106	0.003408228	up	Cytokinin_riboside_5'-monophosphate_phosphoribohydrolase
MELO3C014240.2	-1.420132361	0.000199293	down	Aquaporin_PIP2
MELO3C014247.2	-1.421661049	0.0000307	down	Methyltransferase_type_11

MELO3C014254.2	1.976188377	4.73E-23	up	Cell_number_regulator_8
MELO3C014257.2	1.584206299	0.001143273	up	Arabidopsis_thaliana_genomic_DNA,_chromosome_5,_P1_clone:MOK16
MELO3C014360.2	6.650101654	0.00000877	up	Glycosyltransferase
MELO3C014367.2	-4.925714225	2.16E-10	down	ABC1_family_protein
MELO3C014380.2	2.428847316	0.000123831	up	Vacuolar_iron_transporter-like_protein
MELO3C014465.2	1.273501039	1.11E-08	up	Xyloglucan_endotransglucosylase/hydrolase
MELO3C014505.2	1.613566752	0.0000151	up	NAC_domain-containing_protein_90
MELO3C014516.2	-3.642837574	0.000135011	down	NDR1/HIN1-like_protein_12
MELO3C014568.2	-1.139265608	0.003545524	down	Alanine:glyoxylate_aminotransferase
MELO3C014637.2	-2.351242005	0.000771574	down	linoleate_13S-lipoxygenase_2-1,_chloroplastic-like
MELO3C014701.2	-1.435499799	0.000187896	down	protein_kinase_2B,_chloroplastic-like
MELO3C014730.2	-1.180254827	0.000000351	down	N_utilization_substance_B
MELO3C014803.2	-2.123821462	0.001756305	down	Phosphate_carrier,_mitochondrial
MELO3C014818.2	-4.295398812	8.26E-08	down	Cystinosin-like_protein
MELO3C014821.2	-1.329809339	9.15E-08	down	Cystinosin-like_protein
MELO3C014827.2	-5.429630822	0.00000256	down	Class_I_heat_shock_protein
MELO3C014857.2	-1.046303417	0.002366807	down	Lysine_histidine_transporter
MELO3C014922.2	1.428087563	0.000274042	up	NAC_domain-containing_protein_83_isoform_X2
MELO3C014926.2	-1.280661442	0.000461676	down	chaperone_protein_ClpB3,_chloroplastic
MELO3C015002.2	6.318010621	8.65E-12	up	MLP-like_protein_328
MELO3C015004.2	4.819301172	0.000453279	up	MLP-like_protein_328
MELO3C015007.2	5.011440558	7.02E-08	up	MLP-like_protein_328
MELO3C015011.2	4.402535808	0.004057813	up	MLP-like_protein_329
MELO3C015021.2	9.806849428	7.46E-12	up	MLP-like_protein_329
MELO3C015076.2	-1.228689584	0.00049261	down	Chlorophyll_a-b_binding_protein,_chloroplastic
MELO3C015088.2	2.385435633	0.00000739	up	IQ_domain-containing_protein_IQM2
MELO3C015092.2	4.107205436	0.000000131	up	Transmembrane_protein,_putative
MELO3C015118.2	-2.69702733	4.18E-08	down	Unknown_protein
MELO3C015123.2	2.144510057	2.67E-09	up	Protein_LATERAL_ROOT_PRIMORDIUM_1
MELO3C015140.2	7.210643976	0.000000112	up	Cytochrome_P450
MELO3C015152.2	-1.946396456	1.13E-08	down	NAD(P)-binding_Rossmann-fold_superfamily_protein
MELO3C015183.2	1.887271931	0.002669602	up	phospholipase_A1-Igamma3,_chloroplastic
MELO3C015216.2	3.782919402	4.67E-08	up	beta-glucosidase_12
MELO3C015221.2	-3.596773713	0.000269146	down	cyanogenic_beta-glucosidase-like
MELO3C015222.2	5.018714797	5.29E-09	up	cyanogenic_beta-glucosidase-like
MELO3C015257.2	2.056149412	5.19E-17	up	nicotianamine_synthase-like
MELO3C015283.2	1.118103118	0.00000486	up	calmodulin-binding_protein_60_B-like_isoform_X2
MELO3C015286.2	-1.013384997	0.003564258	down	Glycine-rich_domain-containing_protein_1
MELO3C015288.2	1.807792812	0.0000524	up	Pectinesterase
MELO3C015291.2	1.382699209	0.00000694	up	Pectinesterase_inhibitor,_putative
MELO3C015315.2	-1.423120185	0.0000104	down	protein_indeterminate-domain_12-like
MELO3C015388.2	-2.156672544	0.002297479	down	cytochrome_P450_CYP72A219-like
MELO3C015398.2	-1.278917324	0.000673137	down	cysteine_synthase-like
MELO3C015402.2	1.221557865	7.39E-11	up	guanine_deaminase
MELO3C015406.2	2.092663207	2.58E-12	up	RNA-dependent_RNA_polymerase
MELO3C015408.2	-1.119285418	0.00000426	down	Protein_phosphatase_2C-like_protein
MELO3C015414.2	1.806412792	0.0000197	up	receptor-like_protein_kinase_HSL1
MELO3C015415.2	1.204705806	0.001054651	up	receptor-like_protein_kinase_5
MELO3C015421.2	-1.173612624	0.000113931	down	programmed_cell_death_protein_4-like
MELO3C015424.2	-1.007853248	0.000173134	down	Glucan_endo-1,3-beta-glucosidase,_putative
MELO3C015494.2	-1.175598709	0.000197667	down	Lactoylglutathione_lyase_/_glyoxalase_I_family_protei
MELO3C015598.2	-1.416805428	8.71E-09	down	Peptidyl-tRNA_hydrolase,_putative
MELO3C015682.2	1.118919457	0.0000308	up	Zinc_finger_BED_domain-containing_protein_DAYSLEEPER
MELO3C015696.2	7.559796895	1.09E-20	up	Receptor-like_protein_kinase
MELO3C015699.2	-2.201689439	0.00017222	down	exopolygalacturonase_isoform_X1
MELO3C015738.2	-4.685995212	0.000300029	down	Unknown_protein
MELO3C015849.2	1.780909143	0.000609528	up	Phenazine_biosynthesis_PhzC/PhzF_family_protein
MELO3C015852.2	1.322240956	0.000343838	up	adenylate_isopentenyltransferase_3,_chloroplastic
MELO3C015943.2	2.681750635	0.003913429	up	zinc_finger_protein_6-like
MELO3C015963.2	-1.885211172	0.0002379	down	pectinesterase-like
MELO3C016011.2	-1.485515553	0.000729661	down	ABC_transporter_C_family_member_4-like
MELO3C016031.2	-1.471872151	0.000000331	down	glutathione_S-transferase-like
MELO3C016033.2	-1.016416156	0.0000102	down	glutathione_S-transferase-like
MELO3C016039.2	-2.212379639	0.003647831	down	wee1-like_protein_kinase
MELO3C016053.2	2.191186187	0.001845328	up	Glutaredoxin
MELO3C016055.2	6.073899939	0.0000158	up	Glutaredoxin
MELO3C016167.2	-2.340726777	7.27E-31	down	Glutathione_S-transferase
MELO3C016224.2	-1.231092211	0.000575445	down	9-cis-epoxycarotenoid_dioxygenase,_putative
MELO3C016287.2	-1.157635437	0.002691462	down	Endoglucanase
MELO3C016295.2	-2.317265148	0.0000262	down	At4g23880

MELO3C016322.2	1.316726574	0.00000246	up	cation/H(+)_antiporter_18-like
MELO3C016323.2	1.259385192	0.000105026	up	cation/H(+)_antiporter_18-like
MELO3C016331.2	2.21255041	0.00000144	up	p-loop_nucleoside_triphosphate_hydrolase_superfamily_protein
MELO3C016334.2	-2.326744568	0.000569062	down	Protein_LURP-one-related_17
MELO3C016359.2	1.373948352	6.95E-11	up	Protein_SPIRAL1
MELO3C016384.2	-1.071620546	0.000210937	down	Polygalacturonase_inhibitor
MELO3C016444.2	-1.694999693	0.003925681	down	NAC_domain-containing_protein_55
MELO3C016445.2	6.501386928	0.00000744	up	Hexosyltransferase
MELO3C016449.2	-5.546480624	4.9E-11	down	22.0_kDa_class_IV_heat_shock_protein
MELO3C016540.2	-1.345181225	0.003959565	down	NAC_domain_protein,
MELO3C016557.2	-3.054278036	0.000153973	down	Cyclic_nucleotide-gated_channel
MELO3C016562.2	1.612812873	0.0000554	up	At4g33560
MELO3C016579.2	-1.066031551	0.0000114	down	Serine/threonine-protein_kinase
MELO3C016588.2	-2.774798999	3.08E-10	down	(+)-gamma-cadinene_synthase
MELO3C016593.2	-5.774873084	0.000560626	down	Sesquiterpene_synthase_Tps1
MELO3C016602.2	1.600071077	0.000171209	up	Alkylmercury_lyase
MELO3C016616.2	4.484048709	0.0000446	up	Indole-3-acetic_acid-amido_synthetase_GH3.3
MELO3C016623.2	1.42477431	9.66E-10	up	NAD(P)H_dehydrogenase_(Quinone)
MELO3C016679.2	1.032586495	0.0033577	up	Receptor-like_kinase
MELO3C016771.2	-2.348337422	0.00000715	down	Transferase_family_protein
MELO3C016772.2	2.569751113	0.000049	up	LOW_QUALITY_PROTEIN:_uncharacterized_acetyltransferase_At3g50280-like
MELO3C016806.2	-2.508070174	0.003492736	down	Glycosyl_hydrolase_family_35_protein,_putative
MELO3C016897.2	-3.071666769	4.42E-11	down	17.9_kDa_class_II_heat_shock_protein
MELO3C016932.2	-2.938032339	6.47E-16	down	Eukaryotic_initiation_factor_4F_subunit_p150_isofor
MELO3C016970.2	-1.082908791	0.002078506	down	Chaperone_protein
MELO3C016976.2	-1.797004029	0.002459518	down	zinc_finger_protein_1-like
MELO3C016980.2	-1.789523234	0.002738602	down	ethylene-responsive_transcription_factor_ERF003-like
MELO3C017013.2	1.321446873	0.000686107	up	DNA-directed_RNA_polymerase_subunit_beta
MELO3C017017.2	-1.146514226	0.003803906	down	CoA_ligase
MELO3C017185.2	-1.063146678	0.000000035	down	NAC_domain_protein,
MELO3C017286.2	2.125186124	0.0000924	up	transcription_factor_DICHOTOMA-like
MELO3C017294.2	-9.908720846	1.64E-15	down	Carbon_catabolite_repressor_protein_4_like_3
MELO3C017307.2	1.166778549	0.000001	up	Ribosomal_protein_L34Ae
MELO3C017328.2	-6.475977794	0.0000149	down	Stress_up-regulated_Nod_19_protein
MELO3C017357.2	1.363936913	0.001200609	up	Auxin_efflux_carrier_component
MELO3C017358.2	-3.469960515	2.08E-14	down	C2_domain-containing_protein
MELO3C017473.2	-2.105653204	0.000000281	down	4-coumarate--CoA_ligase_1-like
MELO3C017481.2	1.656766188	8.27E-09	up	Xyloglucan_endotransglucosylase/hydrolase
MELO3C017493.2	-6.836061342	8.26E-25	down	Unknown_protein
MELO3C017540.2	-1.802705081	0.000668388	down	Cytochrome_P450_protein
MELO3C017541.2	-1.336877568	0.001967271	down	receptor-like_protein_kinase_5
MELO3C017542.2	-1.054459972	0.0000631	down	Aldehyde_dehydrogenase
MELO3C017547.2	1.287370847	0.000660387	up	GRAS_family_transcription_factor
MELO3C017574.2	-1.324171251	0.0000207	down	Beta-glucosidase,_putative
MELO3C017582.2	-3.008904449	0.0000426	down	Protein_MARD1
MELO3C017630.2	1.049166228	0.00041103	up	At5g61660
MELO3C017631.2	2.608034189	8.97E-08	up	cyclin-U4-1-like
MELO3C017723.2	2.701467342	0.00000984	up	Histone_demethylase_UTY
MELO3C017729.2	-1.798687416	0.0000466	down	Glutathione_peroxidase
MELO3C017753.2	-1.178769337	0.00062974	down	Glycerophosphodiester_phosphodiesterase,_putative
MELO3C017795.2	1.794412592	0.000357894	up	Inositol_polyphosphate_multikinase
MELO3C017822.2	-6.448914145	0.0000304	down	EG45-like_domain_containing_protein
MELO3C017823.2	-1.08627723	0.00000253	down	protein_NRT1/_PTR_FAMILY_6.1
MELO3C017831.2	3.225771978	0.00000975	up	aquaporin_NIP6-1
MELO3C017883.2	-3.606170122	0.000205177	down	26.5_kDa_heat_shock_protein,_mitochondrial
MELO3C017917.2	-1.958601491	0.002472742	down	Calvin_cycle_protein_CP12,_chloroplastic
MELO3C017928.2	-1.919279569	0.000439845	down	DUF3511_domain_protein,_putative_(DUF3511)
MELO3C017942.2	-1.079888264	0.0000108	down	Sucrose_synthase
MELO3C017950.2	-1.166507524	0.001151713	down	Late_embryogenesis_abundant_3_family_protein
MELO3C018023.2	-1.823160159	0.0000539	down	Sterol_regulatory_element-binding_protein_site_2_protease
MELO3C018055.2	1.180313476	0.000000169	up	MADS_box_protein
MELO3C018057.2	8.077632268	4.57E-10	up	proline-rich_receptor-like_protein_kinase_PERK2_isoform_X2
MELO3C018058.2	6.840959652	2.38E-17	up	Malic_enzyme
MELO3C018065.2	-4.488039095	0.000944173	down	BnaA07g05330D_protein
MELO3C018158.2	6.162971281	0.0000442	up	Ribonuclease_P/MRP_protein_subunit_POP5
MELO3C018166.2	2.399042407	0.001465434	up	Indole-3-acetic_acid-amido_synthetase_GH3.3
MELO3C018174.2	1.117506934	0.000441165	up	zinc_finger_protein_CONSTANS-LIKE_4

MELO3C018248.2	1.035456005	0.000496331	up	Molybdenum_cofactor_sulfurase
MELO3C018316.2	2.266519752	0.000206403	up	subtilisin-like_protease_SBT1.1
MELO3C018335.2	10.70556626	1.4E-18	up	flavonoid_3',5'-hydroxylase_1-like
MELO3C018346.2	-1.129240884	0.0000145	down	Calcium-dependent_protein_kinase
MELO3C018373.2	6.860938303	0.0000573	up	sister_chromatid_cohesion_1_protein_1
MELO3C018385.2	2.27454451	0.00013324	up	cytosolic_endo-beta-N-acetylglucosaminidase_1-like
MELO3C018394.2	1.702475323	0.000441665	up	Abscisic_acid_receptor
MELO3C018422.2	1.244216971	0.001353772	up	1-aminocyclopropane-1-carboxylate_oxidase_2
MELO3C018424.2	4.488125181	4.79E-11	up	1-aminocyclopropane-1-carboxylate_oxidase
MELO3C018425.2	-3.685090722	0.001540857	down	Salicylic_acid-binding_protein_2
MELO3C018454.2	1.247214677	0.000144979	up	Cold_regulated_gene_27,_putative_isoform_3
MELO3C018463.2	-1.377408537	0.0000516	down	CBS_domain-
MELO3C018485.2	-2.81287466	2.24E-10	down	22.0_kDa_class_IV_heat_shock_protein
MELO3C018489.2	-1.369915478	0.000925175	down	Glycosyltransferase
MELO3C018492.2	1.093515558	0.0000242	up	Cinnamyl_alcohol_dehydrogenase
MELO3C018505.2	-1.093841081	0.0000136	down	Upstream_activation_factor_subunit_spp27
MELO3C018522.2	7.542321633	0.000000028	up	endo-1,4-beta-xylanase-like
MELO3C018528.2	-2.295855478	6.85E-09	down	Photosystem_I_reaction_center_subunit_N
MELO3C018547.2	-4.452493099	0.000810393	down	Pathogenesis-related_protein_1
MELO3C018578.2	2.04002148	2.6E-13	up	L-type_lectin-domain_containing_receptor_kinase_IX.1-like
MELO3C018580.2	-11.52558425	2.62E-15	down	Cysteine_proteinase_inhibitor
MELO3C018600.2	-5.524236384	0.000128487	down	IgA_FC_receptor
MELO3C018601.2	1.178275139	0.000795599	up	MADS_box_protein
MELO3C018626.2	10.30183251	1.49E-16	up	12-oxophytodienoate_reductase-like_protein
MELO3C018627.2	2.246641401	0.002818084	up	7-deoxyloganetin_glucosyltransferase-like
MELO3C018630.2	1.950563225	4.24E-11	up	7-deoxyloganetin_glucosyltransferase-like
MELO3C018634.2	5.235557169	7.65E-16	up	7-deoxyloganetin_glucosyltransferase-like
MELO3C018635.2	4.166280484	4.32E-24	up	7-deoxyloganetin_glucosyltransferase-like
MELO3C018643.2	-1.247405751	3.57E-08	down	copper-transporting_ATPase_PAA1,_chloroplastic-like
MELO3C018691.2	-3.418750318	7.04E-08	down	Glycosyltransferase
MELO3C018703.2	2.844092033	0.002665598	up	O-acyltransferase_WSD1-like
MELO3C018733.2	1.7356753	0.0000515	up	MLP_protein
MELO3C018766.2	1.337939584	0.0000997	up	Wound-responsive_family_protein
MELO3C018778.2	-1.540783035	0.000544513	down	Protein_TIC_20-IV,_chloroplastic
MELO3C018793.2	1.350158223	8.74E-08	up	mediator-associated_protein_1-like
MELO3C018797.2	1.994864919	0.0000303	up	Cysteine-rich_receptor-kinase-like_protein
MELO3C018798.2	1.043281887	0.000294347	up	Cysteine-rich_receptor-kinase-like_protein
MELO3C018799.2	5.437531448	5.42E-08	up	Cysteine-rich_receptor-like_kinase
MELO3C018814.2	-1.055784377	0.002336193	down	Protein_DETOXIFICATION
MELO3C018833.2	-1.101885676	0.000199194	down	Allene_oxide_synthase
MELO3C018844.2	1.367046894	2.7E-09	up	homeobox_protein_knotted-1-like_LET6
MELO3C018845.2	1.525461065	0.001446604	up	Basic-leucine_zipper_(BZIP)_transcription_factor_family_protein,_putative
MELO3C018880.2	1.73465767	1.98E-08	up	Glycine-rich_protein
MELO3C018886.2	2.240097523	0.002255881	up	BnaA09g54870D_protein
MELO3C018892.2	-1.289130646	0.002596064	down	Pollen_Ole_e_1_allergen/extensin
MELO3C019099.2	-1.581685875	0.0000871	down	CDPK-related_kinase_1
MELO3C019150.2	-7.10770219	0.000000495	down	Unknown_protein
MELO3C019177.2	3.82856996	0.00000011	up	Glycosyltransferase
MELO3C019199.2	1.929797066	0.0000216	up	Receptor-like_protein_kinase,_putative
MELO3C019239.2	2.274381776	0.001550423	up	Peroxidase
MELO3C019266.2	1.050054372	0.00017501	up	DUF4228_domain-containing_protein
MELO3C019312.2	-1.798891694	0.003770441	down	Basic-
MELO3C019334.2	-2.638659281	0.0000307	down	dnaJ_homolog_subfamily_B_member_13-like
MELO3C019337.2	-1.575031588	0.002231351	down	Two-component_response_regulator-like_APRR2
MELO3C019432.2	-1.093330616	6.12E-11	down	magnesium_transporter_MRS2-4
MELO3C019445.2	-7.864013438	1.1E-09	down	ATP-dependent_DNA_helicase_Q-
MELO3C019451.2	2.944762575	7.81E-09	up	Unknown_protein
MELO3C019529.2	1.10119869	0.000457886	up	Leucine-rich_repeat_receptor-
MELO3C019552.2	-1.262800236	0.003167388	down	Neuronal_PAS_domain_protein
MELO3C019561.2	1.134751677	0.00000692	up	At1g78995
MELO3C019567.2	4.29607642	0.002576405	up	zinc_finger_protein_4-like
MELO3C019700.2	-4.927023101	0.0000603	down	DNA-directed_RNA_polymerases_II_and_IV_subunit_5A
MELO3C019715.2	-1.810304595	0.0000408	down	heat_stress_transcription_factor_A-4c-like
MELO3C019762.2	-1.216047436	0.0000609	down	Protein_trichome_birefringence
MELO3C019814.2	-1.636809193	0.000386034	down	ABC_transporter_G_family_member
MELO3C019835.2	-2.450150705	0.002855333	down	Protein_nuclear_fusion_defective_4
MELO3C019843.2	-3.273589353	0.00000018	down	zinc_finger_protein_ZAT5-like
MELO3C019999.2	1.693677242	1.85E-08	up	WEB_family_protein_At2g40480

MELO3C020000.2	-1.418056643	0.001661368	down	adenine/guanine_permease_AZG1
MELO3C020005.2	-1.498763519	0.0000186	down	Expansin
MELO3C020011.2	8.010389908	4.08E-10	up	PHD_finger_alfin-like_protein
MELO3C020096.2	1.073262682	0.004192331	up	Neuronal_PAS_domain_protein
MELO3C020206.2	1.245773997	0.001190831	up	DELLA_protein_GAI-like
MELO3C020225.2	-7.565165779	0.000000021	down	Myb_transcription_factor
MELO3C020261.2	-1.164826825	8.44E-10	down	C2_domain-containing_protein
MELO3C020264.2	-4.787915349	0.000554809	down	kirola-like
MELO3C020268.2	-6.116774649	0.00119354	down	MLP-like_protein_28
MELO3C020273.2	2.852266015	0.001194125	up	MLP-like_protein_28
MELO3C020281.2	6.135034587	0.000156823	up	Aquaporin
MELO3C020291.2	5.014343798	2.23E-11	up	Little_protein_1
MELO3C020300.2	-1.467368434	0.000555787	down	AAR2_family_protein
MELO3C020311.2	-1.396930238	0.000215082	down	Carboxypeptidase
MELO3C020357.2	-2.06932521	0.001247637	down	Sucrose-phosphate_synthase
MELO3C020363.2	-1.605846221	0.00000398	down	cytochrome_P450_714C2-like
MELO3C020421.2	6.487973415	2.07E-33	up	Unknown_protein
MELO3C020547.2	2.766353999	0.0000236	up	O-glucosyltransferase_rumi_homolog
MELO3C020559.2	-2.7827862	0.002390744	down	GDP-mannose_transporter,_putative
MELO3C020588.2	1.134130642	0.001056581	up	17.5_kDa_class_I_heat_shock_protein
MELO3C020597.2	1.441302942	0.000751099	up	Emb|CAB82975.1
MELO3C020600.2	1.401218619	3.68E-13	up	metal_tolerance_protein_B
MELO3C020618.2	1.054494416	0.00000327	up	Haloacid_dehalogenase-like_hydrolase
MELO3C020636.2	7.133994898	0.00000002	up	Unknown_protein
MELO3C020655.2	1.771087154	0.001315379	up	p-hydroxybenzoic_acid_efflux_pump_subunit_aaeB
MELO3C020676.2	-1.766991444	0.002011996	down	phenolic_glucoside_malonyltransferase_1-like
MELO3C020695.2	-1.413715838	0.000575097	down	TOX_high_mobility_group_box_family_member_4-A,_putative_isoform_2
MELO3C020754.2	2.648616719	2.04E-10	up	auxin-responsive_protein_SAUR23-like
MELO3C020757.2	5.504398071	0.0000459	up	auxin-responsive_protein_SAUR21-like
MELO3C020785.2	2.806656977	0.000797636	up	Proline-rich_protein_2
MELO3C020791.2	2.339946761	0.0000432	up	tRNA_(Guanine(10)-N2)-methyltransferase
MELO3C020958.2	1.193938269	0.000241285	up	Shikimate_kinase
MELO3C020975.2	3.930590313	0.0000047	up	cytochrome_P450_78A7
MELO3C020981.2	1.262832914	0.000593153	up	Receptor-like_kinase
MELO3C020995.2	4.589608247	4.34E-08	up	Unknown_protein
MELO3C021000.2	-4.363793179	0.000105853	down	Heat_Stress_Transcription_Factor_family_protein
MELO3C021015.2	-7.752168181	5.32E-09	down	Histone-lysine_N-methyltransferase_MLL
MELO3C021057.2	-1.439146612	0.000000968	down	RPM1-interacting_protein_4
MELO3C021060.2	-2.628694084	0.00000012	down	Ureide_permease-like_protein
MELO3C021085.2	-2.959639158	0.004200519	down	Membrane_protein_of_er_body-like_protein
MELO3C021100.2	11.9078344	3.87E-23	up	Heat_shock_70_kDa_protein
MELO3C021151.2	1.571892713	0.000651209	up	PLATZ_transcription_factor_family_protein
MELO3C021162.2	1.41141256	0.0000351	up	Selenium-binding_protein
MELO3C021171.2	-2.576855432	0.00000414	down	BAG_family_molecular_chaperone_regulator_6
MELO3C021176.2	-1.257338378	0.000347173	down	delta(8)-fatty-acid_desaturase_2
MELO3C021208.2	-1.808615678	3.87E-08	down	U-box_domain-containing_protein_33-like
MELO3C021249.2	-5.496882227	0.000104833	down	Hexosyltransferase
MELO3C021401.2	1.902531539	0.0000754	up	adenylate_isopentenyltransferase_5,_chloroplastic-like
MELO3C021426.2	6.578704672	0.0000189	up	Protein_TERMINAL_FLOWER_1
MELO3C021474.2	-1.798343726	0.003055464	down	lysosomal_Pro-X_carboxypeptidase-like
MELO3C021479.2	-1.338824332	0.004306417	down	AAA-ATPase_ASD,_mitochondrial-like
MELO3C021482.2	6.503968553	0.0000166	up	Glycosyltransferase
MELO3C021499.2	5.903350187	2.95E-31	up	DUF594_family_protein
MELO3C021548.2	2.241642504	0.00000925	up	BnaC05g50840D_protein
MELO3C021555.2	1.592976568	0.000553621	up	Myb_transcription_factor
MELO3C021578.2	2.008462371	0.00000602	up	LOB_domain-containing_protein_25
MELO3C021607.2	-2.890327755	0.002154951	down	pectate_lyase-like
MELO3C021642.2	4.392217229	0.0000494	up	B-cell_receptor-associated_protein_31-like
MELO3C021671.2	1.448824495	0.00030807	up	BTB/POZ_domain-containing_protein_At5g47800
MELO3C021720.2	2.079469846	0.003601954	up	pyrophosphate--fructose_6-phosphate_1-phosphotransferase_subunit_beta-like
MELO3C021764.2	6.757620053	0.000111324	up	Unknown_protein
MELO3C021772.2	-1.217344398	2.92E-09	down	zinc_finger_protein_ZPR1-like
MELO3C021811.2	-3.328307573	9.02E-19	down	Ribose-5-phosphate_isomerase_A
MELO3C021842.2	3.254414403	1.14E-19	up	Cytochrome_P450
MELO3C021886.2	-2.700052006	8.02E-12	down	S-norcoclaurine_synthase_1-like
MELO3C021901.2	-3.256384914	2.11E-15	down	Alpha/beta_hydrolase-3
MELO3C021917.2	1.001871848	0.003471897	up	Two-component_response_regulator
MELO3C021944.2	-2.005881231	0.000000829	down	Flavoprotein_wrbA
MELO3C021952.2	1.740029279	0.0000342	up	Non-symbiotic_hemoglobin,_putative
MELO3C021965.2	-1.645004975	1.05E-10	down	FAD/NAD(P)-binding_oxidoreductase_family_protein

MELO3C021979.2	-1.409677659	0.001847902	down	Glutaredoxin_family_protein,_putative
MELO3C021982.2	1.697389864	0.00000125	up	ABC_transporter_B_family_protein
MELO3C021999.2	2.337540078	0.00000531	up	Expansin_protein
MELO3C022007.2	-1.717264659	0.004201514	down	transmembrane_emp24_domain-containing_protein_p24delta9
MELO3C022028.2	-2.396016019	0.001263596	down	Transcription_factor_bHLH151,_putative
MELO3C022039.2	2.529426478	0.000000558	up	Unknown_protein
MELO3C022059.2	-2.048743604	5.03E-11	down	Pentatricopeptide_repeat-containing_protein
MELO3C022116.2	-7.037022392	2.3E-22	down	22.0_kDa_class_IV_heat_shock_protein
MELO3C022146.2	2.347119124	8.21E-08	up	TMV_resistance_protein_N-like
MELO3C022147.2	1.237461137	2.54E-08	up	Pentatricopeptide_repeat-
MELO3C022148.2	2.339190297	3.39E-09	up	TMV_resistance_protein_N-like
MELO3C022150.2	3.296786719	0.0000945	up	TMV_resistance_protein_N-like
MELO3C022152.2	3.671721671	4.52E-15	up	TMV_resistance_protein_N-like
MELO3C022160.2	-2.580544468	0.00000778	down	Endoglucanase
MELO3C022207.2	-2.389196887	0.0000468	down	Retrovirus-related_Pol_polyprotein_from_transposon_TNT_1-94
MELO3C022234.2	3.25581406	0.00000729	up	cyclin-U2-1
MELO3C022240.2	-8.930969958	6.74E-12	down	ATP-dependent_RNA_helicase,_putative
MELO3C022248.2	4.743783843	0.000216947	up	NAD-dependent_protein_deacetylase_HST1-
MELO3C022252.2	-1.582054056	0.004018866	down	At2g46730/F19D11.1
MELO3C022257.2	3.262321971	0.000450801	up	protein_AIG1-like
MELO3C022264.2	1.939383145	0.000986158	up	LINE-1_reverse_transcriptase_like
MELO3C022324.2	-1.404689938	0.000867455	down	proton_pump-interactor_2-like_isoform_X2
MELO3C022327.2	-1.861932852	0.0000388	down	Transmembrane_protein,_putative
MELO3C022330.2	5.045409576	0.000960081	up	Protoheme_IX_farnesyltransferase
MELO3C022332.2	1.064000505	0.0000915	up	ferric_reduction_oxidase_8,_mitochondrial
MELO3C022341.2	-6.262002199	0.00000302	down	Bidirectional_sugar_transporter_SWEET
MELO3C022345.2	1.572553481	0.00000252	up	somatic_embryogenesis_receptor_kinase_2-like
MELO3C022372.2	-4.918393461	0.001036207	down	Cytochrome_P450
MELO3C022376.2	-7.22787726	0.000000287	down	Cytochrome_P450
MELO3C022377.2	-6.958193444	0.0000125	down	Cytochrome_P450
MELO3C022378.2	1.212203628	2.98E-09	up	nodulation_receptor_kinase-like
MELO3C022410.2	-1.397252771	0.001821982	down	Tetratricopeptide_repeat_(TPR)-
MELO3C022429.2	-3.581533514	1.36E-08	down	Amaranthin-like_lectin
MELO3C022430.2	-3.527858033	0.00000835	down	Amaranthin-like_lectin
MELO3C022436.2	4.196549112	0.000656308	up	Amaranthin-like_lectin
MELO3C022461.2	1.018198969	0.000314433	up	At2g44600/F16B22.9
MELO3C022499.2	-3.77762284	3.55E-09	down	Myeloid_leukemia_factor
MELO3C022501.2	-1.60535814	0.0000527	down	SNARE-interacting_protein_KEULE
MELO3C022506.2	-3.193675255	0.00000014	down	calmodulin-like_protein_7
MELO3C022542.2	-2.902065584	0.0000342	down	MYB_transcription_factor-like
MELO3C022620.2	3.18099197	0.00000501	up	Alpha/beta-Hydrolases_superfamily_protein
MELO3C022669.2	-1.143088654	0.000589082	down	DUF1677_family_protein_(DUF1677)
MELO3C022704.2	-2.28149791	0.000168733	down	pectinesterase-like
MELO3C022736.2	-3.978002109	4.27E-13	down	Glycosyltransferase
MELO3C022791.2	-2.858576037	0.00000528	down	Protein_MARD1
MELO3C022802.2	2.189582007	2.38E-08	up	Sulfate_transporter,_putative
MELO3C022804.2	-4.730858963	0.0000042	down	heavy_metal-
MELO3C022828.2	2.214087279	0.002136428	up	Glutamine_synthetase
MELO3C022910.2	-1.528458238	0.003427071	down	Structural_maintenance_of_chromosomes_protein
MELO3C022936.2	-1.241965313	0.000431631	down	Triacylglycerol_lipase,_putative
MELO3C022956.2	1.518266844	2.3E-11	up	tRNA-specific_adenosine_deaminase-like_protein_3
MELO3C022965.2	-1.091946263	0.000576981	down	ATP-binding_protein_(P-loop)
MELO3C022980.2	1.914607159	0.003995261	up	WAT1-related_protein
MELO3C022994.2	-1.635567823	0.002787512	down	Phosphate_transporter
MELO3C022997.2	-1.908233503	0.00000261	down	MYB-related_transcription_factor
MELO3C023065.2	-1.953098091	0.00113215	down	Late_embryogenesis_abundant_protein_(LEA)_family_protein
MELO3C023093.2	5.79745078	0.001235429	up	Self-incompatibility_family_protein
MELO3C023156.2	1.830474563	0.000315768	up	Unknown_protein
MELO3C023161.2	-4.314429727	0.000224853	down	nuclear_transcription_factor_Y_subunit_A-7-like
MELO3C023194.2	1.16826467	0.001824659	up	Aspartic_proteinase_Asp1
MELO3C023204.2	1.51902095	0.0000164	up	MYB-related_transcription_factor
MELO3C023220.2	-1.49869206	0.002390448	down	Glutathione_S-transferase
MELO3C023272.2	1.365546761	0.0000516	up	Alcohol_dehydrogenase,_putative
MELO3C023313.2	1.06668464	0.002893032	up	Transmembrane_protein,_putative
MELO3C023338.2	-1.24035377	0.000829805	down	cysteine_proteinase_RD19a-like
MELO3C023360.2	-4.315326396	0.003499898	down	Thionin-like_protein_2
MELO3C023391.2	1.030784911	0.00000818	up	Plant_Tudor-like_protein
MELO3C023403.2	-1.448967889	0.000150428	down	zinc_finger_CCCH_domain-containing_protein_39-
MELO3C023413.2	-1.238152858	0.000265061	down	Signal_peptidase_I

MELO3C023485.2	-1.815743334	0.001903486	down	aspartyl_protease_family_protein_At5g10770
MELO3C023489.2	-3.131261645	0.0000277	down	mitogen-activated_protein_kinase_kinase_kinase_2-
MELO3C023498.2	4.708273688	0.0000053	up	Protein_UPSTREAM_OF_FLC
MELO3C023519.2	-1.215083327	0.0000862	down	Beta-glucosidase
MELO3C023566.2	-2.897291164	6.04E-12	down	LOW_QUALITY_PROTEIN:_putative_disease_resistance_protein_RGA3
MELO3C023577.2	3.88914026	0.000294794	up	disease_resistance_protein_RGA2-like
MELO3C023587.2	-1.198229121	0.000774538	down	Protein_BPS1_chloroplastic
MELO3C023590.2	-1.357053927	1.97E-09	down	Pheophytinase,_chloroplastic
MELO3C023599.2	-1.345958615	0.0000346	down	Glutamine_synthetase
MELO3C023663.2	2.125571974	0.000318777	up	Unknown_protein
MELO3C023738.2	-5.81104222	0.000385704	down	Unknown_protein
MELO3C023772.2	3.894679863	0.002414189	up	Cycloidea-like_protein_group_1A
MELO3C023842.2	2.162980318	0.0000957	up	WAT1-related_protein
MELO3C023843.2	1.144200567	2.31E-09	up	WAT1-related_protein
MELO3C023879.2	-1.223157201	0.001067233	down	Ribulose_bisphosphate_carboxylase/oxygenase_activase_family_protein
MELO3C024014.2	1.277131698	0.001554015	up	Carboxypeptidase
MELO3C024086.2	-4.16294201	1.64E-15	down	70_kDa_heat_shock_protein
MELO3C024163.2	2.636958488	0.002148576	up	acanthoscurrin-1-like
MELO3C024223.2	4.046753546	0.000260933	up	To_encode_a_PR_protein,_Belongs_to_the_plant_thionin_family_with_the_following_members:,_putative
MELO3C024232.2	1.705909654	2.18E-09	up	Transcription_repressor_OFP14
MELO3C024263.2	5.625880516	3.22E-09	up	aquaporin_TIP2-1
MELO3C024269.2	1.600204255	0.000000371	up	Ankyrin_repeat-containing_protein_family
MELO3C024324.2	-1.408175695	0.00000635	down	Chaperone_protein_DnaJ
MELO3C024339.2	-3.766570308	0.00000513	down	NGR2
MELO3C024353.2	-1.008297441	0.000000396	down	phosphoribosylglycinamide_formyltransferase,_chloroplastic
MELO3C024354.2	-1.147246071	0.00000371	down	mitochondrial_ubiquitin_ligase_activator_of_nfkb_1
MELO3C024383.2	-1.732158756	0.00000134	down	beta-fructofuranosidase,_insoluble_isoenzyme_CWINV1-
MELO3C024398.2	-1.40864336	0.0000518	down	ABC_transporter_C_family_member_8
MELO3C024399.2	1.758252455	0.002891481	up	inorganic_pyrophosphatase_2-like
MELO3C024425.2	1.851483558	1.17E-14	up	Receptor-like_protein_kinase
MELO3C024472.2	-1.123833243	0.000000334	down	GATA_zinc_finger_domain-containing_protein_10
MELO3C024483.2	-1.056138659	0.0000176	down	Aquaporin
MELO3C024492.2	1.246832611	0.000000653	up	Protein_SHOOT_GRAVITROPISM_6
MELO3C024504.2	-2.35531015	8.98E-11	down	Sulfite_exporter_TauE/SafE_family_protein
MELO3C024540.2	5.562192209	0.000000028	up	Terminal_flower_1-like_protein
MELO3C024545.2	-1.584558384	0.000526628	down	Transmembrane_9_superfamily_member
MELO3C024560.2	-3.286666974	0.000087	down	NAC_domain-containing_protein_90-like
MELO3C024616.2	5.705884535	0.0000131	up	Unknown_protein
MELO3C024679.2	-1.003671422	0.000853905	down	Myelin-associated_oligodendrocyte_basic_protein_isoform_1
MELO3C024823.2	10.92901251	1.23E-19	up	Receptor-like_protein_kinase
MELO3C024841.2	-5.606138027	0.001581577	down	proline-rich_receptor-like_protein_kinase_PERK2
MELO3C024908.2	-1.143287499	0.00000574	down	RING-type_E3_ubiquitin_transferase
MELO3C024920.2	-2.336642694	6.14E-08	down	zinc_transporter_11
MELO3C024933.2	-1.574483215	0.004090692	down	zinc_finger_protein_36,_C3H1_type-like_2
MELO3C024975.2	2.086460148	4.26E-12	up	DUF3511_domain_protein
MELO3C025019.2	5.674424313	0.00042699	up	Unknown_protein
MELO3C025027.2	-4.031982228	0.0000617	down	Abscisic_stress_ripening-like_protein
MELO3C025034.2	-1.157194106	0.0000189	down	peroxiredoxin_Q,_chloroplastic
MELO3C025079.2	-3.517205709	0.000011	down	universal_stress_protein_A-like_protein
MELO3C025085.2	-2.518448211	4.95E-11	down	Class_I_heat_shock_protein
MELO3C025102.2	-2.320262746	2.3E-10	down	Phytoene_synthase
MELO3C025126.2	-1.181309427	0.000368894	down	cysteine_desulfurase_1,_chloroplastic-like
MELO3C025205.2	-1.268299522	0.000275753	down	RING-type_E3_ubiquitin_transferase
MELO3C025270.2	-1.137782107	0.000915657	down	Late_embryogenesis_abundant_protein,_LEA-14
MELO3C025326.2	2.719708379	0.000687846	up	sulfate_transporter_3.1
MELO3C025345.2	-1.021045127	0.00000413	down	Polyol_transporter_5-like_protein
MELO3C025368.2	4.061327274	2.01E-08	up	BnaC08g10690D_protein
MELO3C025456.2	1.25859169	0.000015	up	WAT1-related_protein
MELO3C025484.2	-1.213099758	4.03E-12	down	chalcone--flavonone_isomerase
MELO3C025485.2	-1.66637801	0.0000195	down	Dirigent_protein
MELO3C025493.2	-1.346001789	0.00000279	down	leukotriene_A-4_hydrolase_homolog
MELO3C025495.2	3.551895445	0.0000756	up	Hydrolase_family_protein/HAD-superfamily_protein
MELO3C025532.2	-1.935439057	0.002745898	down	Structural_maintenance_of_chromosomes_protein
MELO3C025583.2	3.820774887	0.0000179	up	Short-chain_dehydrogenase/reductase
MELO3C025638.2	-1.124451507	7.21E-13	down	Peptide-N4-(N-acetyl-beta-glucosaminyl)asparagine_amidase_A,_putative

MELO3C025640.2	-1.340648818	2.95E-09	down	BnaA05g24630D_protein
MELO3C025641.2	-2.59788168	3.05E-28	down	polynucleotide_3'-phosphatase_ZDP
MELO3C025677.2	-1.679478343	0.002311544	down	protein_SRG1-like
MELO3C025753.2	-1.5875388	0.000815686	down	elongation_factor_1-alpha
MELO3C025761.2	1.338826729	0.0000143	up	MLO-like_protein
MELO3C025764.2	-3.298379586	2.24E-09	down	syntaxin-71_isoform_X1
MELO3C025770.2	1.358833709	0.001109368	up	RING/U-box_protein
MELO3C025772.2	-1.191176821	0.000279831	down	Aquaporin_PIP2
MELO3C025797.2	1.564933293	0.0000116	up	Cytochrome_P450
MELO3C025814.2	1.92372389	0.00000639	up	Ankyrin_repeat-containing_protein
MELO3C025824.2	6.422341467	0.0000224	up	Sucrose-phosphate_synthase_family_protein
MELO3C025837.2	9.039494116	3.4E-12	up	Ankyrin_repeat-containing_protein_family
MELO3C025855.2	-1.992774596	0.00000879	down	Alkyl_transferase
MELO3C025870.2	1.620316724	0.0000128	up	Transcription_factor
MELO3C025914.2	-1.239325129	8.94E-09	down	protein_kinase_APK1A,_chloroplastic-like
MELO3C026001.2	-2.289324288	0.001106012	down	Late_embryogenesis_abundant_protein
MELO3C026043.2	-1.001982509	0.00000722	down	Unknown_protein
MELO3C026134.2	-7.11343433	4.11E-40	down	transcription_factor_IBH1
MELO3C026142.2	-6.425203362	0.00000105	down	Transmembrane_protein,_putative
MELO3C026188.2	-4.5466193	0.0000361	down	omega-hydroxypalmitate_O-feruloyl_transferase
MELO3C026199.2	-2.307268914	2.96E-10	down	Transmembrane_protein,_putative
MELO3C026229.2	1.713409045	1.06E-18	up	argininosuccinate_synthase,_chloroplastic-like
MELO3C026260.2	2.889392313	0.00000694	up	cytochrome_P450_71A22-like
MELO3C026269.2	8.946809346	1.04E-12	up	LOB_domain-containing_protein_25
MELO3C026284.2	1.347949816	0.00000216	up	Embryo_defective_2759_protein,_putative
MELO3C026299.2	2.90335016	0.000101935	up	MADS-box_transcription_factor
MELO3C026372.2	9.524444417	3.03E-15	up	Glycosyltransferase
MELO3C026374.2	-4.314092861	2.45E-14	down	17.5_kDa_class_I_heat_shock_protein
MELO3C026375.2	8.270451869	1.74E-10	up	UDP-glycosyltransferase_71B6-like
MELO3C026380.2	-2.954518448	0.001402048	down	VQ_motif-containing_protein_22
MELO3C026423.2	1.68159956	0.000272215	up	Chlororespiratory_reduction_3
MELO3C026436.2	-1.293555832	4.58E-11	down	1-aminocyclopropane-1-carboxylate_oxidase-1-like_protein
MELO3C026474.2	-1.038375843	0.003220412	down	protein_Ycf2-like
MELO3C026488.2	-1.029756575	0.000963634	down	cytochrome_P450_82A3-like
MELO3C026491.2	-4.289584112	0.003291157	down	cytochrome_P450_CYP82D47-like
MELO3C026492.2	-1.412733084	0.001439923	down	Cytochrome_P450
MELO3C026495.2	-5.918866081	3.19E-12	down	cytochrome_P450_CYP82D47-like
MELO3C026531.2	1.039215778	4.88E-09	up	Receptor_Serine/Threonine_kinase
MELO3C026558.2	-1.845324189	0.000533215	down	Glutamate_receptor
MELO3C026600.2	-3.168380371	0.002221509	down	metal_tolerance_protein_9-like
MELO3C026611.2	-1.371434624	0.00437878	down	Unknown_protein
MELO3C026615.2	-1.031581902	0.0000196	down	Exostosin-like
MELO3C026677.2	4.008306678	0.00000137	up	Receptor_protein_kinase
MELO3C026755.2	-1.519929361	0.003229213	down	Xyloglucan_endotransglucosylase/hydrolase
MELO3C026766.2	4.885822089	4.42E-08	up	Protein_SIEVE_ELEMENT_OCCLUSION_B
MELO3C026781.2	-2.141248561	3.96E-10	down	p-loop_nucleoside_triphosphate_hydrolase_superfamily_protein
MELO3C026784.2	-1.939263698	0.002558445	down	serine/threonine-protein_kinase_BLUS1-like
MELO3C026805.2	5.119410483	7.73E-23	up	Coiled-coil_protein_(DUF572)
MELO3C026808.2	6.288430785	0.00038224	up	SABATH_methyltransferase_9
MELO3C026860.2	-1.074955278	0.000454806	down	Protein_MIZU-KUSSEI_1
MELO3C026901.2	-1.144221238	0.0000112	down	HNH_endonuclease
MELO3C026992.2	-1.060077406	0.000000486	down	sugar_transport_protein_14-like
MELO3C027004.2	-4.384161048	2.93E-12	down	poly(A)-specific_ribonuclease_PARN-like
MELO3C027009.2	-3.89681754	4.57E-32	down	Unknown_protein
MELO3C027013.2	1.759286247	0.000783813	up	polyol_transporter_5-like
MELO3C027015.2	-5.140323614	0.003927904	down	ACT_domain-containing_protein
MELO3C027083.2	-1.093945537	0.001677789	down	Metal-nicotianamine_transporter
MELO3C027093.2	-3.968337352	0.003334796	down	DExH-box_ATP-dependent_RNA_helicase_DExH12-
MELO3C027124.2	-6.278330371	1.71E-15	down	17.5_kDa_class_I_heat_shock_protein
MELO3C027138.2	-1.907062166	0.00000057	down	leukotriene_A-4_hydrolase_homolog
MELO3C027172.2	-2.91817693	0.001517312	down	MLP-like_protein_28
MELO3C027180.2	6.313146077	0.0000346	up	Transmembrane_protein,_putative
MELO3C027216.2	1.197778647	0.0000714	up	Transcription_factor,_putative
MELO3C027218.2	8.696023359	6.47E-12	up	O-fucosyltransferase_family_protein
MELO3C027285.2	-7.535188562	0.00000736	down	Unknown_protein
MELO3C027297.2	-1.628131127	3.39E-08	down	Myelin-associated_oligodendrocyte_basic_protein_isoform_1
MELO3C027385.2	-1.087018186	0.0000576	down	Vat_protein
MELO3C027441.2	-1.413681266	0.000000246	down	Unknown_protein

MELO3C027442.2	10.93023554	9.37E-20	up	Beta-glucosidase_18
MELO3C027615.2	2.048547925	0.0000192	up	Vat_protein
MELO3C027629.2	-5.834881217	0.000327977	down	Unknown_protein
MELO3C027646.2	10.59804952	3.47E-18	up	Unknown_protein
MELO3C027663.2	-9.417203586	3.95E-13	down	Unknown_protein
MELO3C027727.2	-2.47596751	9.01E-14	down	(-)-germacrene_D_synthase-like
MELO3C027753.2	6.401987588	0.000000224	up	Unknown_protein
MELO3C027756.2	7.070689857	2.53E-08	up	centromere/kinetochore_protein_zw10_homolog
MELO3C027872.2	6.100521622	5.48E-19	up	Unknown_protein
MELO3C027880.2	5.084976966	0.000778792	up	LOW_QUALITY_PROTEIN:_gamma-aminobutyrate_transaminase_POP2,_mitochondrial-
MELO3C027893.2	-5.62398844	0.001002863	down	Unknown_protein
MELO3C027955.2	-6.021091792	0.00000871	down	Transposase
MELO3C027975.2	11.59812886	0.000000887	up	Cysteine_proteinase_inhibitor
MELO3C028021.2	-7.30200452	0.000000613	down	Unknown_protein
MELO3C028047.2	-6.13093669	0.000066	down	Transposon_protein,_putative,_CACTA,_En/Spm_sub-class
MELO3C028107.2	6.230350432	0.0000248	up	Unknown_protein
MELO3C028132.2	-5.603015519	0.003057965	down	70_kDa_heat_shock_protein
MELO3C028143.2	1.682038129	0.000375756	up	Subtilisin-like_protease
MELO3C028156.2	-2.387257353	0.001824774	down	LOW_QUALITY_PROTEIN:_gamma-aminobutyrate_transaminase_POP2,_mitochondrial-
MELO3C028179.2	2.404528867	3.57E-14	up	To_encode_a_PR_protein,_Belongs_to_the_plant_thionin_family_with_the_following_members:,_putative
MELO3C028186.2	6.132988006	0.0000402	up	Unknown_protein
MELO3C028281.2	-5.597740367	0.000633461	down	Sulfite_exporter_TauE/SafE_family_protein
MELO3C028301.2	7.918137099	1.28E-09	up	Unknown_protein
MELO3C028427.2	-5.716034834	0.000396266	down	LOW_QUALITY_PROTEIN:_gamma-aminobutyrate_transaminase_POP2,_mitochondrial-
MELO3C028509.2	3.755462096	2.52E-09	up	Unknown_protein
MELO3C028514.2	-6.429675382	0.0000123	down	Unknown_protein
MELO3C028526.2	-4.046650599	7.35E-31	down	Unknown_protein
MELO3C028531.2	7.714718723	1.38E-12	up	DEAD-box_ATP-dependent_RNA_helicase_28
MELO3C028543.2	4.535979102	1.61E-08	up	Ty3-gypsy_retrotransposon_protein
MELO3C028565.2	5.096984539	1.7E-27	up	Thioredoxin-like_protein
MELO3C028577.2	1.8601173	0.000268347	up	CCR4-NOT_transcription_complex_subunit_1
MELO3C028586.2	-1.229224405	0.0000896	down	2-alkenal_reductase_(NADP(+)-dependent)-like
MELO3C028593.2	-3.903986118	0.000101215	down	Protein_DETOXIFICATION
MELO3C028764.2	-1.933870901	0.00000322	down	K(+)_efflux_antiporter_5-like
MELO3C028786.2	6.577529017	0.00000449	up	Unknown_protein
MELO3C028813.2	-7.372099005	1.54E-45	down	CACTA_en-spm_transposon_protein
MELO3C028851.2	9.998718802	4.59E-16	up	Unknown_protein
MELO3C028889.2	3.220873072	5.7E-11	up	Unknown_protein
MELO3C028900.2	12.13293843	2.17E-29	up	LOW_QUALITY_PROTEIN:_gamma-aminobutyrate_transaminase_POP2,_mitochondrial-
MELO3C028901.2	11.5178533	1.73E-21	up	NBS-LRR_type_resistance_protein
MELO3C028906.2	6.450253316	0.0000086	up	LOW_QUALITY_PROTEIN:_gamma-aminobutyrate_transaminase_POP2,_mitochondrial-
MELO3C028908.2	5.319823743	5.65E-10	up	Unknown_protein
MELO3C028923.2	-1.935786839	0.000792813	down	Unknown_protein
MELO3C028938.2	-5.709074748	0.000378816	down	Unknown_protein
MELO3C028959.2	2.094502312	0.003763737	up	disease_resistance_protein_RGA2-like
MELO3C028971.2	1.018953864	0.004441602	up	protein_FAR1-RELATED_SEQUENCE_6
MELO3C029039.2	-1.609476359	0.0000449	down	COBRA-like_protein_2
MELO3C029054.2	3.955282863	0.000621535	up	Isoflavone_reductase_like
MELO3C029064.2	5.195475507	0.003129236	up	Unknown_protein
MELO3C029078.2	6.389880663	0.0000813	up	Unknown_protein
MELO3C029079.2	6.238229965	0.0000664	up	Unknown_protein
MELO3C029096.2	8.906203571	6.61E-13	up	Unknown_protein
MELO3C029108.2	1.006827353	3.12E-08	up	Glyceraldehyde-3-phosphate_dehydrogenase
MELO3C029112.2	10.39612361	8.84E-18	up	Transmembrane_protein,_putative
MELO3C029124.2	-8.272659056	1.75E-21	down	Sodium_Bile_acid_symporter_family
MELO3C029147.2	-7.693351448	2.19E-15	down	Unknown_protein
MELO3C029148.2	-10.4937684	3.72E-17	down	Diphthine_synthase-like_protein
MELO3C029163.2	-1.103940933	0.0000849	down	Unknown_protein
MELO3C029171.2	-12.12345969	5.05E-24	down	heparan-alpha-glucosaminide_N-acetyltransferase
MELO3C029178.2	6.193919714	0.00019917	up	Protein_PHOTOPERIOD-INDEPENDENT_EARLY_FLOWERING_1
MELO3C029187.2	1.179888375	0.001641069	up	D-amino_acid_dehydrogenase
MELO3C029196.2	8.898596831	1.06E-12	up	rac-like_GTP-binding_protein_ARAC7
MELO3C029197.2	-5.12498749	0.0000309	down	Unknown_protein
MELO3C029199.2	11.49117328	9.07E-21	up	26S_proteasome_non-ATPase_regulatory_subunit_12

MELO3C029352.2	-2.450022238	1.3E-09	down	non-specific_phospholipase_C2
MELO3C029430.2	2.244456953	2.16E-21	up	Unknown_protein
MELO3C029502.2	-10.30481231	5.78E-16	down	Retrovirus-related_Pol_polyprotein_from_transposon_TNT_1-94
MELO3C029512.2	-1.036138263	0.001134346	down	BNR/Asp-box_repeat_family_protein
MELO3C029517.2	-1.590204689	7.87E-09	down	Unknown_protein
MELO3C029559.2	-7.415830671	9.32E-12	down	Unknown_protein
MELO3C029575.2	5.299177538	0.000000436	up	Unknown_protein
MELO3C029614.2	4.418862378	3.14E-19	up	Unknown_protein
MELO3C029618.2	2.240097661	0.001588613	up	LOW_QUALITY_PROTEIN:_gamma-aminobutyrate_transaminase_POP2,_mitochondrial-
MELO3C029630.2	10.43230297	1.25E-17	up	Serine/threonine-protein_kinase
MELO3C029680.2	-8.006082044	9.75E-10	down	Vacuolar_protein_sorting-associated_protein_32-like_protein_2
MELO3C029682.2	-1.715405873	0.001096268	down	Transcriptional_corepressor_LEUNIG
MELO3C029693.2	-7.770361028	2.83E-09	down	Retrovirus-related_Pol_polyprotein_from_transposon_TNT_1-94
MELO3C029694.2	-8.486326306	1.87E-11	down	Myb/SANT-like_DNA-binding_domain_protein
MELO3C029695.2	2.421074266	9.82E-12	up	Unknown_protein
MELO3C029702.2	3.645140412	0.000739471	up	type_I_inositol_polyphosphate_5-phosphatase_4
MELO3C029703.2	5.166791011	0.0000338	up	Unknown_protein
MELO3C029721.2	4.221754361	1.96E-80	up	Core-2/I-branching_beta-1,6-N-acetylglucosaminyltransferase_family_protein
MELO3C029738.2	-2.010587684	0.000116802	down	Terpene_cyclase/mutase_family_member
MELO3C029795.2	-10.75991586	2.86E-11	down	Unknown_protein
MELO3C029817.2	-3.254993919	0.00000105	down	Receptor-kinase,_putative
MELO3C029895.2	-1.670889851	0.00000699	down	lon_protease_homolog_2,_peroxisomal_isoform_X2
MELO3C029915.2	-6.478652777	0.0000229	down	Retrovirus-related_Pol_polyprotein_from_transposon_TNT_1-94
MELO3C029950.2	5.005344342	1.1E-29	up	Unknown_protein
MELO3C029961.2	6.087725462	0.0000709	up	Dead_box_ATP-dependent_RNA_helicase,_putative
MELO3C029971.2	-8.708118625	9.11E-12	down	IST1-like_protein
MELO3C030037.2	6.406545411	0.0000208	up	Unknown_protein
MELO3C030083.2	2.877485175	0.001500475	up	zinc_finger_BED_domain-containing_protein_RICESLEEPER_2-like
MELO3C030156.2	-6.575743743	0.00000412	down	Calcium_binding_protein
MELO3C030166.2	-6.960115337	0.00000168	down	60S_ribosomal_protein_L7a
MELO3C030213.2	4.353230625	0.00000184	up	Transmembrane_protein,_putative
MELO3C030259.2	-1.016967457	0.000160078	down	exocyst_complex_component_SEC6
MELO3C030287.2	1.038540974	0.001346119	up	WRKY_transcription_factor,_putative
MELO3C030310.2	-2.937692412	0.000000205	down	Unknown_protein
MELO3C030315.2	-6.583773273	1.62E-16	down	UBN2_3_domain-containing_protein
MELO3C030366.2	4.862108132	0.001719934	up	Microspore-specific_promoter_2,_putative
MELO3C030434.2	10.32281062	3.45E-13	up	LOW_QUALITY_PROTEIN:_putative_pentatricopeptide_repeat-containing_protein_At3g23330
MELO3C030506.2	-4.325269652	0.00000417	down	Cytochrome_P450
MELO3C030508.2	6.366147062	0.0000353	up	Unknown_protein
MELO3C030522.2	9.391080121	1.17E-14	up	Unknown_protein
MELO3C030576.2	10.99110943	4.62E-19	up	zinc_finger_BED_domain-containing_protein_RICESLEEPER_2-
MELO3C030586.2	5.295374264	0.003751228	up	Zf-CCHC_domain-containing_protein/MP_domain-containing_protein
MELO3C030635.2	10.06786971	1.3E-16	up	Unknown_protein
MELO3C030673.2	3.190417782	0.00000429	up	Unknown_protein
MELO3C030700.2	1.171065355	0.0000667	up	AAA-ATPase_ASD,_mitochondrial-like
MELO3C030711.2	3.238018506	0.000283465	up	Beta-glucosidase
MELO3C030721.2	-5.011460557	0.000124804	down	Beta-glucosidase,_putative
MELO3C030722.2	-7.340326626	0.00000284	down	Beta-glucosidase,_putative
MELO3C030748.2	3.032174305	0.000134063	up	Two-component_response_regulator
MELO3C030754.2	5.766731384	0.000333905	up	Unknown_protein
MELO3C030768.2	10.5984152	3.86E-18	up	Aspartic_proteinase
MELO3C030769.2	8.394343615	2.63E-11	up	beta-glucosidase_18-like
MELO3C030787.2	-2.86552447	0.000764042	down	gibberellin_2-beta-dioxygenase_2
MELO3C030795.2	2.78047009	1.92E-14	up	Serine/threonine-protein_kinase_EDR1
MELO3C030825.2	8.275327361	1.09E-10	up	Unknown_protein
MELO3C030837.2	-8.138959727	4.85E-08	down	zinc_finger_BED_domain-containing_protein_RICESLEEPER_2-like
MELO3C030859.2	-2.999434043	1.01E-22	down	Unknown_protein
MELO3C030860.2	5.063413397	0.0000475	up	Splicing_factor_U2af_large_subunit_B
MELO3C030868.2	-4.610894776	0.000259188	down	GDSL_esterase/lipase_1-like
MELO3C030882.2	5.610608158	0.0000567	up	zinc_finger_BED_domain-containing_protein_RICESLEEPER_2-like

MELO3C030897.2	3.744892341	0.0000258	up	L10-interacting_MYB_domain-containing_protein
MELO3C030900.2	9.655970294	5.66E-15	up	GDT1-like_protein_1,_chloroplastic_isoform_X2
MELO3C030913.2	6.941439132	0.000000384	up	60S_ribosomal_protein_L7
MELO3C030988.2	-6.406702442	0.000000106	down	Unknown_protein
MELO3C030990.2	3.514755404	0.000746042	up	cytochrome_P450_94A1-like
MELO3C030992.2	-5.496727488	0.001078035	down	histone-lysine_N-
MELO3C031036.2	1.932522689	0.00392072	up	Unknown_protein
MELO3C031037.2	2.405105904	0.0000762	up	Unknown_protein
MELO3C031042.2	-1.415314426	8.06E-11	down	Unknown_protein
MELO3C031049.2	-1.337080231	5.8E-09	down	Peroxidase
MELO3C031114.2	4.81063455	0.000148609	up	lysine--tRNA_ligase-like
MELO3C031144.2	-4.590592453	1.87E-16	down	Unknown_protein
MELO3C031171.2	-1.535262053	0.00000371	down	Glycine_cleavage_system_H_protein,_mitochondrial
MELO3C031239.2	-1.41355274	0.000210704	down	Unknown_protein
MELO3C031272.2	5.390336041	0.001303283	up	Unknown_protein
MELO3C031314.2	1.947383918	0.001202904	up	Ubiquitin-conjugating_enzyme,_E2
MELO3C031323.2	6.779986331	0.0000186	up	LOW_QUALITY_PROTEIN:_disease_resistance_prote in_RPP4-like
MELO3C031330.2	-3.503237561	0.0000751	down	Vat_protein
MELO3C031342.2	2.566866068	0.00000674	up	UDP-sugar_transporter-like_protein
MELO3C031355.2	-2.880117393	0.001673497	down	zinc_finger_BED_domain-containing_protein_RICESLEEPER_2-like
MELO3C031416.2	6.22853548	0.000278476	up	Unknown_protein
MELO3C031423.2	-5.250010056	0.003592398	down	Unknown_protein
MELO3C031450.2	-4.712454822	1.76E-19	down	GDSL_esterase/lipase_At5g22810
MELO3C031452.2	1.948608364	0.000000683	up	Amino_acid_permease
MELO3C031506.2	4.988755852	0.000787219	up	Unknown_protein
MELO3C031571.2	1.56995413	0.004162184	up	UDP-sugar_transporter_sqv-7-like_isoform_X2
MELO3C031591.2	1.228667038	0.000152259	up	U3_small_nucleolar_RNA-associated_protein_14_homolog_A
MELO3C031601.2	-2.38183959	0.0000729	down	Glutathione_S-transferase
MELO3C031621.2	-2.361310623	0.001012167	down	Wall-associated_receptor_kinase-like_20
MELO3C031755.2	8.228230152	7.68E-15	up	Unknown_protein
MELO3C031780.2	2.191960359	0.000230577	up	Unknown_protein
MELO3C031809.2	6.598175705	5.19E-34	up	transcription_factor_LAF1-like_isoform_X2
MELO3C031841.2	-10.73377695	3.74E-17	down	Unknown_protein
MELO3C031863.2	-1.339512452	0.000000134	down	Calcium_uniporter_protein,_mitochondrial
MELO3C031865.2	8.789512081	1.51E-11	up	Transmembrane_protein,_putative
MELO3C031893.2	3.130747493	0.00267662	up	protein_disulfide_isomerase-like_1-6
MELO3C031926.2	3.15673768	2.83E-16	up	Unknown_protein
MELO3C032004.2	5.348302654	4.91E-27	up	Unknown_protein
MELO3C032071.2	9.2164701	1.85E-13	up	26S_protease_regulatory_subunit_S10B
MELO3C032074.2	1.757021633	0.000144395	up	cytochrome_P450_CYP82D47-like
MELO3C032096.2	-4.148770137	3.13E-17	down	Retrovirus-related_Pol_polyprotein_from_transposon_TNT_1-94
MELO3C032098.2	7.110357081	0.00000508	up	At4g32050
MELO3C032121.2	4.684435377	0.0000285	up	PIF-like_transposase
MELO3C032128.2	2.534273221	0.000000139	up	Calcium-binding_protein
MELO3C032143.2	-4.367235891	0.000842448	down	Unknown_protein
MELO3C032192.2	4.347807148	1.15E-09	up	clustered_mitochondria_protein
MELO3C032227.2	5.750388465	0.00083598	up	Hexosyltransferase
MELO3C032322.2	3.846601879	0.00000459	up	Unknown_protein
MELO3C032345.2	10.363103	3.68E-17	up	Plant_cadmium_resistance_2,_putative
MELO3C032418.2	5.779139753	0.000632879	up	kinesin-like_protein_KIN12B
MELO3C032454.2	-10.90738821	8.85E-19	down	PHD_finger_protein_ALFIN-LIKE_3-like_isoform_X1
MELO3C032461.2	-2.256647395	2.46E-10	down	Protein_SRG1
MELO3C032499.2	-2.099263605	0.000111775	down	Unknown_protein
MELO3C032537.2	-6.346303721	0.0000195	down	alanine--tRNA_ligase-like
MELO3C032550.2	6.66675648	0.0000107	up	Aldo/keto_reductase_family_protein
MELO3C032563.2	-1.243369322	0.000000389	down	Glutathione_S-transferase
MELO3C032606.2	-7.394276083	8.43E-08	down	Myb/SANT-like_DNA-binding_domain_protein
MELO3C032627.2	-1.187076802	0.001067908	down	Transcription_factor_IIIB_90_kDa_subunit
MELO3C032832.2	-2.667979844	3.22E-16	down	gibberellin_2-beta-dioxygenase_8
MELO3C032833.2	-4.446537417	0.0000382	down	Autophagy-related_protein_3
MELO3C032862.2	-3.417159963	0.00000146	down	Unknown_protein
MELO3C032879.2	-1.426619903	0.00361841	down	Unknown_protein
MELO3C032888.2	-1.371440399	0.000654314	down	glucose-induced_degradation_protein_8_homolog_isoform_X2
MELO3C032937.2	-1.580261268	0.00010706	down	glucomannan_4-beta-mannosyltransferase_9-like
MELO3C032944.2	11.25761931	1.96E-20	up	Transcription_factor
MELO3C033011.2	1.609785982	0.00364437	up	Unknown_protein
MELO3C033024.2	7.803856795	4.59E-09	up	Exostosin_family_protein

MELO3C033048.2	11.26406732	7.77E-21	up	ras-related_protein_Rab11C-like
MELO3C033070.2	8.508973914	4.89E-11	up	MYB-domain_protein
MELO3C033082.2	6.830085351	0.000000245	up	Unknown_protein
MELO3C033119.2	3.931080468	0.000128087	up	Cysteine_proteinase_inhibitor
MELO3C033157.2	6.644206995	0.000337921	up	Threonine_dehydratase
MELO3C033195.2	-3.867824076	0.000180123	down	glutathione_S-transferase_U8-like
MELO3C033198.2	-2.334037543	0.004045649	down	Glutathione_S-transferase
MELO3C033230.2	-2.242905629	3.68E-13	down	BZIP_transcription_factor
MELO3C033241.2	-7.613152078	6.45E-09	down	Unknown_protein
MELO3C033295.2	9.35578945	5.94E-14	up	Unknown_protein
MELO3C033329.2	-6.656234336	0.00000558	down	Unknown_protein
MELO3C033346.2	8.48790905	1.41E-15	up	Alpha/beta-Hydrolases_superfamily_protein
MELO3C033353.2	-11.42451102	1.72E-21	down	60S_ribosomal_protein_l11
MELO3C033373.2	-5.943534415	1.04E-14	down	pre-mRNA-splicing_factor_CWC25_homolog
MELO3C033414.2	7.476552344	2.77E-08	up	Integrase,_catalytic_core
MELO3C033425.2	1.361687917	0.00000841	up	Unknown_protein
MELO3C033427.2	7.66638011	5.2E-09	up	Unknown_protein
MELO3C033464.2	7.931045982	3.15E-10	up	L10-interacting_MYB_domain-containing_protein
MELO3C033652.2	-6.253625272	3.82E-30	down	Unknown_protein
MELO3C033665.2	-7.759183794	2.09E-09	down	Unknown_protein
MELO3C033679.2	8.128543524	8.6E-10	up	NBS-LRR_type_resistance_protein
MELO3C033703.2	-6.283591031	4.67E-11	down	Gag-pol_polyprotein
MELO3C033743.2	2.916024156	0.002135404	up	Cytochrome_P450
MELO3C033753.2	-3.502786801	0.0000449	down	Purple_acid_phosphatase
MELO3C033800.2	-3.119386369	0.000984192	down	Unknown_protein
MELO3C033879.2	4.396534593	0.000424687	up	WD-40_repeat-containing_MSI4-like_protein
MELO3C033896.2	7.023896242	0.00000254	up	pentatricopeptide_repeat-
MELO3C033898.2	3.941680897	3.02E-11	up	zinc_finger_BED_domain-containing_protein_RICESLEEPER_2-like
MELO3C033914.2	1.011721308	0.003735537	up	serine/threonine-protein_kinase_STY8-like
MELO3C033917.2	-3.163908588	0.000357941	down	LOW_QUALITY_PROTEIN:_gamma-aminobutyrate_transaminase_POP2,_mitochondrial-
MELO3C033943.2	2.220799141	0.000206412	up	LOW_QUALITY_PROTEIN:_gamma-aminobutyrate_transaminase_POP2,_mitochondrial-
MELO3C033952.2	4.74697837	0.00000986	up	Unknown_protein
MELO3C034006.2	2.593985321	0.00012935	up	Arabinogalactan_peptide-like_protein
MELO3C034028.2	-2.737235591	1.44E-11	down	Protein_DETOXIFICATION
MELO3C034063.2	5.822114087	0.0000236	up	Unknown_protein
MELO3C034084.2	5.348638941	0.0000162	up	Unknown_protein
MELO3C034100.2	-4.804000101	4.72E-08	down	Class_I_heat_shock_protein
MELO3C034116.2	-3.254818631	7.76E-11	down	17.5_kDa_class_I_heat_shock_protein
MELO3C034119.2	-6.9162047	1.61E-19	down	17.5_kDa_class_I_heat_shock_protein
MELO3C034144.2	-2.636435648	0.002365492	down	Unknown_protein
MELO3C034188.2	1.067025204	0.000773956	up	Rhodanese-related_sulfurtransferase
MELO3C034248.2	9.567086705	4.14E-15	up	Cytochrome_P450
MELO3C034276.2	5.319380084	4.75E-08	up	katanin_p60_ATPase-
MELO3C034288.2	6.40182189	0.0000192	up	cyclin-dependent_kinases_regulatory_subunit_1-like
MELO3C034293.2	7.500799133	1.98E-09	up	Unknown_protein
MELO3C034361.2	7.536408915	9.57E-09	up	BTB_domain-containing_protein
MELO3C034371.2	-6.616572196	0.000000895	down	Transposase
MELO3C034376.2	9.691485141	2.39E-15	up	Monodehydroascorbate_reductase
MELO3C034379.2	9.622207592	1.1E-14	up	BEACH_domain-containing_protein_C2
MELO3C034389.2	-7.86027084	5.45E-09	down	Thioredoxin
MELO3C034410.2	2.612976911	0.002215426	up	zinc_finger_BED_domain-containing_protein_RICESLEEPER_2-like
MELO3C034414.2	7.276693238	0.000000634	up	Aspartic_proteinase
MELO3C034439.2	-7.14862924	0.000000266	down	Ribosomal_protein
MELO3C034534.2	7.319850048	4.05E-08	up	nudix_hydrolase_14,_chloroplastic
MELO3C034547.2	5.545793053	0.00019383	up	zinc_finger_BED_domain-containing_protein_RICESLEEPER_2-like
MELO3C034878.2	-8.55283151	1.04E-11	down	E3_ubiquitin-protein_ligase_UPL6-like_protein
MELO3C034879.2	-11.40088275	2.38E-21	down	Aspartic_proteinase
MELO3C035031.2	5.596300571	1.08E-22	up	Phosphatidate_phosphatase_PAH2
MELO3C035307.2	10.0787965	1.69E-16	up	Unknown_protein
MELO3C035318.2	-5.062624759	0.0000126	down	tetraspanin-3-like
MELO3C035349.2	-1.687302996	0.002421442	down	alpha_carbonic_anhydrase_7-like
MELO3C035414.2	11.50965444	1.79E-21	up	RING/FYVE/PHD_zinc_finger_superfamily_protein
MELO3C035417.2	6.624960538	1.57E-17	up	NSP-interacting_kinase
MELO3C035435.2	2.606449982	0.000000048	up	transportin-3
MELO3C035544.2	2.134502856	3.07E-11	up	Transposon_Ty3-G_Gag-Pol_polyprotein
MELO3C035546.2	1.484723389	0.00000746	up	pheophorbide_a_oxygenase,_chloroplastic
MELO3C035583.2	-2.242311658	0.002467442	down	Unknown_protein

MELO3C035594.2	1.8730026	0.0000007	up	lactation_elevated_protein_1
MELO3C035652.2	4.222987992	8.93E-09	up	Methyltransferase-like
MELO3C035710.2	4.942108376	3.79E-12	up	Binding_protein
MELO3C035718.2	-1.80149667	0.001978534	down	Transmembrane_protein,_putative
MELO3C035729.2	-3.511708764	0.0000035	down	cysteine-rich_receptor-like_protein_kinase_28

Significantly enriched Go terms

Go Term	P value	FDR
Biological Process:oxidation reduction(GO:0055114)	0.0000027	0.00073
Biological Process:defense response(GO:0006952)	0.00016	0.022
Biological Process:defense response(GO:0006953)	0.00042	0.028
Biological Process:defense response(GO:0006954)	0.00038	0.028
Biological Process:defense response(GO:0006955)	0.00058	0.032
Biological Process:defense response(GO:0006956)	0.00072	0.032
Biological Process:defense response(GO:0006957)	0.001	0.035
Biological Process:defense response(GO:0006958)	0.001	0.035
Biological Process:defense response(GO:0006959)	0.0000039	0.00015
Biological Process:defense response(GO:0006960)	0.0000011	0.00015
Biological Process:defense response(GO:0006961)	0.0000044	0.00015
Biological Process:defense response(GO:0006962)	0.0000046	0.00015
Biological Process:defense response(GO:0006963)	0.000058	0.0016
Biological Process:defense response(GO:0006964)	0.00017	0.0038
Biological Process:defense response(GO:0006965)	0.00021	0.0041
Biological Process:defense response(GO:0006966)	0.00025	0.0042
Biological Process:defense response(GO:0006967)	0.00044	0.0066
Biological Process:defense response(GO:0006968)	0.00067	0.0091
Biological Process:defense response(GO:0006969)	0.0013	0.015
Biological Process:defense response(GO:0006970)	0.0013	0.015
Biological Process:defense response(GO:0006971)	0.0015	0.015
Biological Process:defense response(GO:0006972)	0.0017	0.016
Biological Process:defense response(GO:0006973)	0.0025	0.023
Biological Process:defense response(GO:0006974)	0.0043	0.034
Biological Process:defense response(GO:0006975)	0.0043	0.034
Biological Process:defense response(GO:0006976)	0.00052	0.015
Biological Process:defense response(GO:0006977)	0.00052	0.015

Functional categorization

Gene Function	Gene number	P value	Padj
stress	88	1.2145E-15	3.751E-14
misc	98	6.50435E-07	1.008E-05
secondary metabolism	45	1.82222E-06	1.881E-05
transport	73	3.47549E-06	2.697E-05
metal handling	11	0.000432377	0.0026807
minor CHO metabolism	11	0.01773301	0.0803452
N-metabolism	4	0.01814247	0.0803452
hormone metabolism	39	0.02136869	0.0828037
development	42	0.03016866	0.1039143
redox	14	0.04785103	0.1483382
cell wall	25	0.147903	0.4168175
signalling	57	0.1688771	0.4362658
polyamine metabolism	2	0.2002769	0.4775834
major CHO metabolism	5	0.3291317	0.6849026
gluconeogenesis	1	0.3314045	0.6849026
photosynthesis	12	0.4076314	0.7897858
glycolysis	4	0.4419202	0.8058545
tetrapyrrole synthesis	2	0.5100256	0.8162201
nucleotide metabolism	6	0.5242558	0.8162201
TCA	3	0.5346476	0.8162201
fermentation	1	0.5529233	0.8162201
OPP	1	0.6488121	0.9142352
lipid metabolism	13	0.7904428	0.999924
Biodegradation of Xenobiotics	1	0.8079032	0.999924
Co-factor and vitamine metabolism	2	0.8619106	0.999924

amino acid metabolism	8	0.8640327	0.999924
DNA	14	0.8992658	0.999924
RNA	87	0.9463366	0.999924
mitochondrial electron transport	1	0.9934159	0.999924
cell	17	0.9944796	0.999924
protein	78	0.999924	0.999924
not assigned	511	-	-

Table S5 The DEGs information, enriched Gene Ontology terms and functional categorization of these DEGs between two Bulks

DEGs identified between two bulks.

Gene_name	log2FoldChange	P-value	Status	Function annotation
MELO3C000019.2	1.387501697	3.25E-17	up	zinc_finger_protein_ZAT10-like
MELO3C000108.2	-1.126427104	0.001373621	down	50S_ribosomal_protein_L16,_chloroplastic
MELO3C000157.2	1.087267595	4.33E-15	up	Universal_stress_protein_A-like_protein
MELO3C000200.2	-1.329320525	4.67E-16	down	Bidirectional_sugar_transporter_SWEET
MELO3C000210.2	-1.021325112	1.43E-20	down	Unknown_protein
MELO3C000220.2	-1.061094434	0.000402781	down	Glycosyltransferase
MELO3C000247.2	2.378825222	0.009367026	up	NAC_domain-containing_protein,_putati
MELO3C000268.2	-3.917728902	4.39E-55	down	Lipoxygenase
MELO3C000589.2	1.32616083	0.002742734	up	Kinase_family_protein
MELO3C000668.2	-2.100697213	3.50E-11	down	Protein_DETOXIFICATION
MELO3C000792.2	4.91973901	0.005685796	up	Unknown_protein
MELO3C000922.2	-1.422485657	0.000147007	down	NAC_domain-containing_protein,_putati
MELO3C000924.2	-3.65071993	8.99E-05	down	NAD(P)H-quinone_oxidoreductase_s
MELO3C000937.2	3.516375736	1.09E-19	up	Unknown_protein
MELO3C001014.2	1.659443257	9.25E-11	up	protein_NRT1/_PTR_FAMILY_4.6-like
MELO3C001058.2	-3.206188669	1.29E-15	down	Lipoxygenase
MELO3C001274.2	2.42420799	2.82E-61	up	mitochondrial_uncoupling_protein_5-like
MELO3C001368.2	1.011316913	5.31E-06	up	Unknown_protein
MELO3C001387.2	-2.284889257	0.007831201	down	ATP_synthase_subunit_c,_chloroplastic
MELO3C001561.2	-1.036840924	0.000120839	down	Unknown_protein
MELO3C001656.2	1.383409459	5.17E-06	up	Unknown_protein
MELO3C001951.2	4.095519398	0.003734674	up	Xyloglucan_endotransglucosylase/hydrolase
MELO3C001977.2	-2.261965245	0.005693877	down	Glutathione_s-transferase
MELO3C001988.2	-1.060680051	0.003763329	down	Calcium-binding_EF-hand_family_protein,_puta
MELO3C002014.2	1.039607418	8.37E-23	up	SNAP25_homologous_protein_SNAP33
MELO3C002028.2	-1.81134672	9.42E-12	down	Formate_hydrogenlyase_subunit_5
MELO3C002051.2	-1.890936597	0.000338887	down	Adenine_nucleotide_alpha_hydrolases-
MELO3C002076.2	2.320341921	1.17E-06	up	Transcription_factor_TEOSINTE_BRANCHED_1
MELO3C002084.2	1.011769732	4.82E-17	up	Protein_NEGATIVE_REGULATOR_OF_RESISTAN
MELO3C002098.2	2.733735116	7.01E-11	up	Core-2/I-branching_beta-1,6-N-
MELO3C002128.2	-1.249608768	1.70E-21	down	Thaumatin
MELO3C002140.2	-1.38885425	9.64E-06	down	Kinase_family_protein
MELO3C002150.2	1.232326716	1.48E-23	up	mitogen-activated_protein_kinase_k
MELO3C002191.2	-3.40838368	5.91E-14	down	Short-chain_dehydrogenase/redu

MELO3C002192.2	-3.81497736	2.50E-44	down	Cytochrome_P450
MELO3C002193.2	-4.554658545	2.27E-06	down	BAHD_acyltransferase_At 5g47980-like
MELO3C002210.2	-1.121201778	4.87E-19	down	HPP
MELO3C002220.2	1.039304013	2.00E-23	up	AAA-ATPase_At3g28580-like
MELO3C002221.2	1.423184686	1.37E-27	up	AAA-ATPase_At3g28580-like
MELO3C002222.2	1.049899256	2.31E-27	up	AAA-ATPase_At3g28580-like
MELO3C002277.2	-1.263320479	9.43E-14	down	Glutathione_transport_system_permease_protein_gsi
MELO3C002286.2	1.09188911	4.63E-09	up	Receptor_protein_kinase,_putative
MELO3C002287.2	1.592383591	2.93E-11	up	Raffinose_synthase_family_protein
MELO3C002310.2	-3.737247586	6.40E-87	down	cytochrome_P450_71A1-like
MELO3C002311.2	-1.836176447	9.71E-13	down	Cytochrome_P450
MELO3C002341.2	1.838358115	1.77E-08	up	At5g50335
MELO3C002374.2	-1.151614791	0.000185901	down	11-beta-hydroxysteroid_dehydroge
MELO3C002379.2	-1.118312377	0.00057984	down	Protein_tapetum_determinant_1
MELO3C002383.2	1.118755909	1.90E-07	up	transcription_factor_bHLH 137
MELO3C002385.2	-1.722319573	0.00041725	down	Transcription_factor_Inducer_of_CBF_expression_1
MELO3C002392.2	2.625615013	0.003770404	up	LRR_receptor-like_serine/threonine-
MELO3C002420.2	1.307571215	0.00032625	up	Retrovirus-related_Pol_polyprotein_fr
MELO3C002460.2	1.254778575	4.65E-09	up	At3g20340
MELO3C002487.2	-1.083134144	0.000657073	down	Harpin_inducing_protein_1-like_9
MELO3C002491.2	-1.362844049	0.001053587	down	cysteine-rich_receptor-like_protein_kinase_25
MELO3C002495.2	1.941415368	7.69E-40	up	cysteine-rich_receptor-like_protein_kinase_29
MELO3C002514.2	1.139379373	4.20E-11	up	BTB/POZ_domain-containing_protein_At3g22
MELO3C002520.2	-1.507550665	0.000103799	down	(3S,6E)-nerolidol_synthase_1-like
MELO3C002548.2	2.388372206	0.002227925	up	B-box_zinc_finger_protein_3
MELO3C002555.2	-1.369493062	6.19E-21	down	external_alternative_NAD(P)H-
MELO3C002572.2	1.589166811	1.32E-33	up	Carboxypeptidase
MELO3C002573.2	-2.675842172	1.07E-65	down	NAC_transcription_factor_25-like
MELO3C002608.2	-1.149854434	5.26E-28	down	transcription_factor_bHLH 118-like
MELO3C002615.2	1.375754106	0.008454901	up	Late_embryogenesis_abundant_protein_(LEA)_famil
MELO3C002624.2	2.244447745	7.66E-63	up	ethylene-responsive_transcription_fa
MELO3C002637.2	-1.601936992	6.12E-06	down	Nudix_hydrolase_15,_mitochondrial
MELO3C002678.2	1.101812321	4.18E-23	up	RING-type_E3_ubiquitin_transfer
MELO3C002692.2	1.221180671	2.53E-09	up	GDSL_esterase/lipase
MELO3C002720.2	-1.099653074	0.002052333	down	Cytochrome_b561_domain-containing_protein
MELO3C002739.2	-2.801970244	5.94E-05	down	Cotton_fiber_protein
MELO3C002741.2	1.148549946	3.30E-05	up	Protein_TIFY_5A

MELO3C002749.2	-1.61821085	8.65E-30	down	RACK1C
MELO3C002756.2	-1.163952743	1.40E-07	down	Inositol_transporter
MELO3C002759.2	1.013464858	2.92E-11	up	Chitinase_family_protein
MELO3C002763.2	-1.577041819	5.39E-10	down	Galactosyltransferase_family_protein
MELO3C002777.2	-2.331476109	8.83E-13	down	Salicylic_acid-binding_protein_2
MELO3C002875.2	1.788694892	3.47E-15	up	WRKY_family_transcription_factor
MELO3C002880.2	1.662756995	3.37E-23	up	Unknown_protein
MELO3C002925.2	1.239704637	1.10E-18	up	Cysteine_proteinase_inhibitor
MELO3C002936.2	1.060124824	1.32E-11	up	Metalloendoproteinase_1,_putative
MELO3C002952.2	1.269758001	3.28E-32	up	phosphate_transporter_PHO1_homolog_3-like
MELO3C002971.2	1.189138777	0.002788385	up	Ankyrin_repeat-containing_protein
MELO3C003095.2	-1.959689358	2.47E-10	down	heavy_metal-associated_isoprenylated_p
MELO3C003118.2	-1.543631235	6.97E-11	down	p-hydroxybenzoic_acid_efflu
MELO3C003131.2	1.219259504	4.19E-19	up	Phosphatase_2C_family_protein
MELO3C003148.2	1.319345258	5.99E-22	up	Phosphate_transporter_PHO1-like_protein_5
MELO3C003164.2	-1.445640683	3.73E-13	down	Cysteine/Histidine-rich_C1_domain_family_p
MELO3C003177.2	1.028587446	8.23E-26	up	protein_NRT1/_PTR_FAMILY_5.6
MELO3C003186.2	-1.481594969	4.10E-29	down	Proteasome_inhibitor-related
MELO3C003188.2	-1.067412379	1.41E-08	down	glutathione_S-transferase_U8-like
MELO3C003195.2	-1.450985802	1.99E-33	down	17.5_kDa_class_I_heat_shock_protein
MELO3C003201.2	-1.304728407	1.40E-06	down	Glutathione_S-transferase
MELO3C003230.2	1.351622495	1.43E-31	up	BAHD_acyltransferase_DCR-like
MELO3C003275.2	3.324998418	3.94E-19	up	Peroxidase
MELO3C003294.2	-1.163645755	1.83E-36	down	vacuolar_cation/proton_exchanger_3
MELO3C003318.2	1.713721901	7.95E-28	up	phospholipase_A1-Ibeta2,_chloroplastic
MELO3C003321.2	1.346632836	1.91E-08	up	Low-temperature-induced_65_kDa_protein
MELO3C003366.2	-2.949477311	5.30E-11	down	Cytochrome_c_oxidase_subunit
MELO3C003371.2	1.753741131	3.06E-08	up	Expansin-like_protein
MELO3C003375.2	1.372284371	2.64E-43	up	Two-component_response_regul
MELO3C003390.2	1.224432419	1.80E-07	up	NAC_domain_protein,
MELO3C003392.2	-2.645191453	0.010815301	down	Aluminum-activated_malate_transport
MELO3C003492.2	-1.288390139	1.24E-19	down	transcription_factor_bHLH61-like
MELO3C003544.2	1.213990628	0.010638187	up	E3_ubiquitin-protein_ligase_ATL15-like
MELO3C003566.2	1.661044457	4.45E-26	up	Protein_EXORDIUM-like_1
MELO3C003567.2	-2.360191326	6.43E-13	down	Glycosyltransferase
MELO3C003745.2	1.496199228	2.22E-35	up	Thaumatin-like_protein_1
MELO3C003791.2	1.339815844	1.79E-31	up	Thioredoxin

MELO3C003803.2	1.717243735	1.37E-11	up	jasmonate_O-methyltransferase
MELO3C003817.2	1.949942487	2.46E-36	up	DNA_polymerase_epsilon_catalytic_subunit_A
MELO3C003822.2	-3.083872659	0.002746259	down	Cysteine/Histidine-rich_C1_domain_family_p
MELO3C003823.2	-1.14383432	3.62E-07	down	jacalin-related_lectin_3-like
MELO3C003868.2	2.813019891	1.20E-06	up	cytochrome_P450_734A6-like
MELO3C003889.2	-1.194598232	0.000132877	down	Calmodulin
MELO3C003911.2	1.082437168	1.32E-21	up	protein_BONZAI_3
MELO3C003990.2	-1.04467429	2.17E-20	down	BURP_domain_protein_RD22
MELO3C003996.2	1.296318983	1.59E-18	up	Heavy_metal_ATPase_3
MELO3C004003.2	-1.453755651	1.35E-29	down	La-related_protein_6_isoform
MELO3C004059.2	-1.175224321	1.14E-26	down	(R)-mandelonitrile_lyase_1-
MELO3C004060.2	-4.190065195	8.77E-05	down	(R)-mandelonitrile_lyase_1-
MELO3C004089.2	2.05433384	8.10E-05	up	Xyloglucan_endotransglucosylase/hydrolase
MELO3C004090.2	-1.088823934	8.65E-06	down	CASP-like_protein
MELO3C004160.2	1.03499535	3.20E-07	up	protein_NIM1-INTERACTING_1-like
MELO3C004181.2	1.678482293	2.16E-24	up	homeobox_protein_knotted-1-like_1_isoform_X1
MELO3C004184.2	-1.172416798	0.000665829	down	nicotianamine_synthase-like
MELO3C004185.2	-1.731359642	5.03E-22	down	nicotianamine_synthase_1-like
MELO3C004213.2	1.318899579	3.05E-08	up	F-box_family_protein,_putati
MELO3C004242.2	1.043014125	2.12E-07	up	Lipoxygenase
MELO3C004244.2	1.148120194	1.59E-10	up	Lipoxygenase
MELO3C004245.2	-3.01746982	6.75E-27	down	Lipoxygenase
MELO3C004246.2	-5.580495021	1.39E-08	down	Lipoxygenase
MELO3C004250.2	1.704133145	9.56E-11	up	Lipoxygenase
MELO3C004291.2	1.620143256	1.45E-08	up	TMV_resistance_protein_N-like
MELO3C004292.2	2.646712988	0.00013533	up	TMV_resistance_protein_N-like_isoform_X1
MELO3C004374.2	-1.739536974	0.00024931	down	Calcium-transporting_ATPase
MELO3C004385.2	1.267304077	8.44E-16	up	pathogenesis-related_protein_PR-4-like
MELO3C004408.2	-1.72102168	0.000140733	down	Sigma_factor_binding_protein_1,_chloroplastic
MELO3C004414.2	-1.262170598	0.000173547	down	Prolyl_4-hydroxylase_alpha-
MELO3C004430.2	-1.336299485	5.15E-42	down	NADP-dependent_glyceraldehyde-
MELO3C004455.2	-1.156620357	0.01094521	down	blue_copper_protein-like
MELO3C004527.2	2.939041334	7.58E-22	up	Cytochrome_P450
MELO3C004556.2	-1.418705261	9.42E-38	down	heat_stress_transcription_factor_C-1-like
MELO3C004570.2	1.866371033	1.41E-05	up	Protein_SPEAR1
MELO3C004592.2	2.186022275	9.51E-13	up	protein_DETOXIFICATION_51
MELO3C004601.2	1.253514891	9.94E-06	up	acid_phosphatase_1-like

MELO3C004626.2	2.750853394	2.32E-07	up	cytokinin_dehydrogenase_ 3-like
MELO3C004642.2	-1.372252772	5.22E-38	down	Photosystem_II_PsbX
MELO3C004685.2	-2.01129701	2.47E-19	down	FANTASTIC_four-like_protein_(DUF3049)
MELO3C004694.2	1.557435768	2.12E-20	up	NAC_domain-containing_protein_90-like
MELO3C004742.2	1.744232328	3.39E-19	up	cytochrome_P450_94C1
MELO3C004753.2	9.042977423	6.83E-10	up	subtilisin-like_protease_SBT3.18
MELO3C004801.2	-1.069446133	2.26E-19	down	cytochrome_P450_71A1-like
MELO3C004853.2	-2.007182669	5.67E-05	down	30S_ribosomal_protein_S3 ,_chloroplastic
MELO3C004864.2	-1.054260268	6.44E-12	down	Cyclin-dependent_kinases_regulat
MELO3C004869.2	1.413124937	0.000502976	up	protein_SENSITIVE_TO_ PROTON_RHIZOTOXICI
MELO3C004914.2	1.22832353	0.000871987	up	Cysteine_proteinase_inhibi tor
MELO3C004974.2	-2.408708594	0.00081544	down	Protein_nuclear_fusion_def ective_4
MELO3C004984.2	-4.080885991	9.85E-06	down	tetrahydrocannabinolic_aci d_synthase-like
MELO3C005190.2	1.804753169	1.66E-45	up	chitinase_10
MELO3C005215.2	1.595512711	2.59E-14	up	defensin_Ec-AMP-D2-like
MELO3C005227.2	1.749510999	5.05E-09	up	Unknown_protein
MELO3C005275.2	1.296633899	9.07E-21	up	nudix_hydrolase_18,_mito chondrial
MELO3C005297.2	-1.38748324	3.96E-14	down	NADH_dehydrogenase-like_complex_L
MELO3C005308.2	1.861793118	1.14E-43	up	Serinethreonine-protein_kinase_ctr1
MELO3C005319.2	2.325765743	1.36E-22	up	Calcium-binding_protein
MELO3C005356.2	-1.33112195	4.79E-21	down	Protein_CHUP1,_chloropla stic
MELO3C005367.2	1.529945789	1.60E-16	up	dehydration-responsive_element-
MELO3C005374.2	1.886048429	1.44E-10	up	receptor-like_protein_kinase
MELO3C005405.2	-1.333154725	1.66E-21	down	CDGSH_iron-sulfur_domain-
MELO3C005427.2	1.577953266	3.86E-22	up	LRR_receptor-like_kinase
MELO3C005435.2	-1.650763875	0.000210894	down	Receptor_protein_kinase,_ putative
MELO3C005436.2	-1.487948099	0.00163071	down	Receptor_protein_kinase,_ putative
MELO3C005465.2	1.685768052	8.28E-50	up	ethylene-responsive_transcription_fa
MELO3C005476.2	1.368992989	5.23E-05	up	Auxin-responsive_protein
MELO3C005541.2	-2.103995594	9.49E-05	down	14_kDa_proline-rich_protein_dc2.15
MELO3C005553.2	5.472340021	0.007629537	up	LOW_QUALITY_PROTEI N:_protein_NRT1/_PTR_F
MELO3C005565.2	-1.120084455	2.04E-31	down	Elongation_factor_G,_chlo roplastic
MELO3C005607.2	-1.871002689	2.27E-26	down	Cytochrome_P450,_putativ e
MELO3C005610.2	-5.667800489	0.006084265	down	transcription_factor_bHLH 36-like
MELO3C005611.2	-1.446455358	2.35E-11	down	transcription_factor_bHLH 120-like
MELO3C005630.2	1.82263258	3.56E-12	up	ethylene-responsive_transcription_fa
MELO3C005637.2	1.288819551	7.58E-35	up	Rhomboid-like_protein

MELO3C005643.2	1.580427822	3.05E-15	up	Thaumatin-like_protein
MELO3C005699.2	-1.336853921	1.05E-15	down	LOW_QUALITY_PROTEIN:_homeobox_protein_BE
MELO3C005709.2	-2.790863186	1.46E-10	down	Heavy_metal-associated_domain_protein
MELO3C005710.2	-1.141331137	6.24E-13	down	heavy_metal-associated_isoprenylated_p
MELO3C005712.2	1.43143016	7.28E-08	up	heavy_metal-associated_isoprenylated_p
MELO3C005720.2	1.568871615	6.20E-17	up	Late_embryogenesis_abundant_protein
MELO3C005736.2	-1.111728799	1.05E-28	down	Cytoplasmic_tRNA_2-thiolation_protein
MELO3C005763.2	-1.062054477	4.44E-28	down	Glutaredoxin
MELO3C005784.2	-2.39984911	8.55E-05	down	DUF538_family_protein
MELO3C005786.2	-3.46087394	0.008787504	down	Cystathionine_gamma-synthase
MELO3C005796.2	1.415034086	1.42E-17	up	Avr9/Cf-9_rapidly_elicited_protein
MELO3C005811.2	-1.02753025	1.48E-08	down	RING-H2_finger_protein_ATL79
MELO3C005839.2	1.16038279	3.22E-14	up	Glycoside_hydrolase_family_28_protein/polygalacturo
MELO3C005843.2	1.901888107	0.000119997	up	WRKY_transcription_factor
MELO3C005859.2	1.946696907	6.72E-89	up	acidic_endochitinase-like
MELO3C005917.2	-1.085617127	9.06E-28	down	Membrane-bound_transcription_factor
MELO3C005923.2	-1.381726558	1.72E-34	down	Chloroplast_small_heat_shock_protein
MELO3C005947.2	1.255479345	2.08E-10	up	basic_blue_protein
MELO3C005960.2	1.070251851	0.000277121	up	zinc_finger_protein_ZAT9
MELO3C005962.2	1.501444794	0.000764808	up	Beta-expansin
MELO3C005972.2	-1.040453644	3.87E-09	down	ethylene-responsive_transcription_fa
MELO3C005992.2	-1.041150795	8.93E-14	down	homeobox-leucine_zipper_protein_AT
MELO3C006032.2	-1.057959207	1.38E-11	down	Oxygen-evolving_enhancer_3-
MELO3C006037.2	1.844122322	2.99E-46	up	WRKY_transcription_factor
MELO3C006070.2	1.982870284	8.87E-20	up	acidic_endochitinase-like
MELO3C006081.2	1.540657186	1.25E-17	up	WRKY_transcription_factor_22
MELO3C006139.2	2.790958082	1.08E-32	up	BON1-associated_protein_2
MELO3C006152.2	1.667155329	8.46E-18	up	At4g00770
MELO3C006228.2	-1.325244643	1.06E-11	down	cyclin-U4-1
MELO3C006230.2	-5.914582418	0.002860453	down	AT-hook_motif_nuclear-localized_protein_23
MELO3C006246.2	-1.031072702	2.86E-22	down	ABC_transporter_family_protein
MELO3C006353.2	-1.741874713	9.68E-05	down	Glutathione_S-transferase
MELO3C006365.2	-1.015844778	5.52E-11	down	FBT8
MELO3C006417.2	1.740872636	1.02E-10	up	Protein_LIGHT-DEPENDENT_SHORT_H
MELO3C006431.2	1.719268966	8.43E-11	up	ethylene-responsive_transcription_fa
MELO3C006439.2	1.245900804	1.25E-22	up	1-aminocyclopropane-1-carboxylate_oxidase_homo
MELO3C006457.2	2.493159271	0.007398926	up	Valine--tRNA_ligase

MELO3C006464.2	-2.04637399	0.000108156	down	Proteinase_inhibitor
MELO3C006483.2	1.177809346	1.57E-06	up	Non-specific_serine/threonine_p
MELO3C006493.2	-1.141980818	7.94E-34	down	ATP_sulfurylase
MELO3C006499.2	-1.883558441	2.41E-77	down	Early_light-induced_protein
MELO3C006501.2	-1.0517261	7.32E-05	down	heat_stress_transcription_factor_A-6b
MELO3C006546.2	1.038922708	4.11E-07	up	BRI1_kinase_inhibitor_1-like
MELO3C006552.2	-1.5486311	7.20E-10	down	Glucose-1-phosphate_adenylyltransfer
MELO3C006571.2	1.942118086	1.38E-08	up	adenylate_isopentenyltransferase_5,_chloroplastic
MELO3C006577.2	-1.269170932	2.88E-40	down	Glutamine_synthetase
MELO3C006580.2	1.668144696	0.000137618	up	S-adenosylmethionine_decar
MELO3C006608.2	-1.172884749	0.009184516	down	CASP-like_protein
MELO3C006616.2	-1.433383114	2.38E-08	down	DNA_ligase_1-like
MELO3C006682.2	1.151271834	1.46E-19	up	RING-H2_finger_protein_ATL2
MELO3C006688.2	1.347356868	0.001263073	up	Ethylene-responsive_transcription_fa
MELO3C006728.2	1.53301811	1.43E-10	up	transcription_factor_MYB108
MELO3C006773.2	1.130706	1.44E-16	up	Rapid_ALkalinization_Factor
MELO3C006774.2	-3.773762648	1.67E-13	down	MLP-like_protein_43
MELO3C006775.2	-2.305196743	0.000658226	down	MLP-like_protein_43
MELO3C006810.2	-1.568688333	3.32E-14	down	peroxisomal_membrane_protein_11B
MELO3C006814.2	1.577001136	0.00014944	up	NAC_domain-containing_protein_7-like
MELO3C006869.2	3.594278438	7.38E-92	up	CRT/DRE-binding_factor_1
MELO3C006870.2	1.945747141	5.52E-09	up	ethylene-responsive_transcription_fa
MELO3C006874.2	2.817364042	1.26E-51	up	Protein_DETOXIFICATION
MELO3C006904.2	-1.023979511	1.75E-15	down	Remorin
MELO3C006944.2	-1.038951841	0.000263056	down	ferric_reduction_oxidase_4-like
MELO3C006946.2	1.066932122	8.96E-13	up	Membrane-associated_kinase_regulato
MELO3C006947.2	1.193698607	1.89E-05	up	ABC_transporter_G_family_member_8
MELO3C006958.2	-1.347152909	1.25E-09	down	Protein_PAM68_chloroplastic
MELO3C007037.2	1.028950878	1.38E-15	up	Patatin
MELO3C007055.2	1.12394083	1.08E-30	up	reticuline_oxidase-like_protein
MELO3C007127.2	1.121586549	1.22E-08	up	9-cis-epoxycarotenoid_dioxygen
MELO3C007132.2	1.932826206	1.06E-29	up	Gibberellin_2-oxidase
MELO3C007134.2	1.098020007	0.000215311	up	Inner_membrane_protein_oxaA
MELO3C007146.2	-1.867166935	1.09E-14	down	Thiamine_monophosphate_synthase
MELO3C007202.2	2.35698697	0.000677205	up	At2g42760
MELO3C007215.2	1.358585785	3.50E-06	up	RING-type_E3_ubiquitin_transfer
MELO3C007227.2	1.025293489	1.50E-19	up	Pyruvate_decarboxylase

MELO3C007241.2	2.158081595	1.27E-08	up	ethylene-responsive_transcription_fa
MELO3C007253.2	1.762817333	1.28E-35	up	Glutaredoxin
MELO3C007255.2	-1.390057237	6.76E-07	down	NAC_domain-containing_protein
MELO3C007256.2	-1.256477905	0.000268036	down	PTI1-like_tyrosine-protein_kinase_At3g15890
MELO3C007267.2	1.208236992	9.96E-34	up	ethylene-responsive_transcription_fa
MELO3C007279.2	-1.641952315	0.002732904	down	Pollen_Ole_e_1_allergen_and_extensin_family_protei
MELO3C007315.2	-1.029618275	9.83E-15	down	Internal_alternative_NAD(P)H-
MELO3C007321.2	2.045942192	9.01E-56	up	F-box_protein_SKIP27
MELO3C007337.2	2.083030521	1.33E-19	up	Zinc_finger,_B-box
MELO3C007341.2	1.432822605	1.10E-25	up	transcription_factor_bHLH112-like_isoform_X1
MELO3C007378.2	-1.157737242	5.63E-27	down	glucose-6-phosphate/phosphate_trans
MELO3C007391.2	1.963858303	1.64E-46	up	Glycosyl_transferase,_family_31
MELO3C007398.2	-1.324374907	2.34E-41	down	5'-adenylylsulfate_reductase
MELO3C007399.2	1.131023844	1.83E-12	up	Rhamnogalacturonate_lyase
MELO3C007444.2	1.203652625	8.04E-25	up	Scarecrow-like_protein_13-
MELO3C007470.2	1.908032567	4.24E-20	up	WRKY_family_transcription_factor
MELO3C007482.2	-1.453307781	1.96E-18	down	Cytochrome_P450_family_protein
MELO3C007524.2	-4.098717823	0.005257711	down	B3_domain-containing_transcription_fa
MELO3C007531.2	1.059477292	4.11E-10	up	Salicylic_acid-binding_protein_2
MELO3C007537.2	-2.77457035	0.008512719	down	GLABRA2_expression_modulator
MELO3C007545.2	1.173531656	6.20E-13	up	Peroxidase
MELO3C007547.2	-1.05427506	8.66E-09	down	AT3g50560/T20E23_160
MELO3C007553.2	3.571112054	0.000288516	up	DNA-directed_RNA_polymerase
MELO3C007564.2	-1.130447972	2.32E-30	down	Lipase
MELO3C007565.2	1.868083112	1.59E-26	up	mitogen-activated_protein_kinase_k
MELO3C007588.2	-1.435983875	0.001654684	down	Transmembrane_protein,_putative
MELO3C007597.2	2.082499249	1.10E-07	up	Indole-3-acetic_acid-amido_synthetase_GH3.3
MELO3C007630.2	1.281681337	4.33E-26	up	protein_SHORT-ROOT
MELO3C007654.2	1.452482424	2.18E-06	up	Phytosulfokines_3_family_protein
MELO3C007663.2	-1.410423693	0.001353236	down	transcription_factor_RAX2
MELO3C007687.2	1.120226879	8.62E-11	up	Phosphoenolpyruvate_carboxykinase
MELO3C007698.2	-1.470257035	5.65E-51	down	Serine_hydroxymethyltransferase
MELO3C007720.2	-1.509429415	6.66E-23	down	transcription_factor_bHLH131-like
MELO3C007727.2	2.193414984	0.000284896	up	zinc-finger_homeodomain_prote
MELO3C007786.2	1.226660221	9.75E-13	up	BnaA10g22140D_protein
MELO3C007793.2	-1.899121812	9.91E-18	down	cytochrome_P450_CYP736A12-like
MELO3C007799.2	-1.180408053	2.43E-07	down	cytochrome_P450_CYP736A12-like

MELO3C007856.2	-1.422051198	4.70E-05	down	Cytochrome_P450_family_protein
MELO3C007860.2	2.067554911	6.84E-10	up	AAA-ATPase_At2g18193-like
MELO3C007868.2	-1.53696438	4.27E-05	down	Peroxidase
MELO3C007877.2	4.470294607	1.68E-126	up	Protein_SHI_RELATED_SEQUENCE_1
MELO3C007893.2	-1.044839121	0.000685532	down	auxin-induced_protein_15A
MELO3C007915.2	-1.799604517	1.56E-48	down	oligopeptide_transporter_7-like
MELO3C007926.2	1.569162437	4.15E-06	up	heavy_metal-associated_isoprenylated_p
MELO3C007949.2	-1.531432769	1.42E-35	down	thiosulfate_sulfurtransferase_16,_chloroplastic
MELO3C007961.2	1.305991703	6.30E-22	up	Chitinase
MELO3C007966.2	1.973547772	1.33E-73	up	endochitinase-like
MELO3C008014.2	-1.852438692	3.23E-05	down	ethylene-responsive_transcription_fa
MELO3C008026.2	2.03455155	7.97E-10	up	WAT1-related_protein
MELO3C008082.2	1.50741948	7.58E-06	up	ethylene-responsive_transcription_fa
MELO3C008203.2	3.319454322	3.07E-94	up	protein_LYK2
MELO3C008204.2	2.271868207	2.42E-18	up	Calcium-binding_protein
MELO3C008286.2	1.743262361	2.84E-70	up	nematode_resistance_protein-like_HSPRO2
MELO3C008309.2	-1.173419575	6.50E-08	down	LOB_domain-containing_protein_21
MELO3C008342.2	1.207756473	1.73E-08	up	WAT1-related_protein
MELO3C008375.2	-2.282139481	5.82E-20	down	Unknown_protein
MELO3C008442.2	1.293200027	7.38E-13	up	wall-associated_receptor_kinase
MELO3C008455.2	-1.131853694	0.00216501	down	LOW_QUALITY_PROTEIN:_probable_serine/threoni
MELO3C008478.2	-1.154714647	1.50E-23	down	Protein_phosphatase_2c,_putative
MELO3C008515.2	1.476610472	5.18E-22	up	ARGOS-like_protein
MELO3C008657.2	-1.840330786	2.04E-31	down	Ammonium_transporter
MELO3C008659.2	-1.270836016	0.007520495	down	Ammonium_transporter
MELO3C008666.2	1.778477495	0.004175151	up	GDSL_esterase/lipase
MELO3C008802.2	1.292443968	3.25E-29	up	Glutamate_dehydrogenase
MELO3C008804.2	1.756221805	2.30E-25	up	Methyltransferase-like_protein
MELO3C008920.2	-1.275573132	5.06E-28	down	chloroplast_stem-loop_binding_protein_of_4
MELO3C009027.2	-1.198909141	1.89E-26	down	carboxyl-terminal-processing_peptidase_3,_c
MELO3C009034.2	-1.80405337	0.009223218	down	DNA-dependent_metalloprotease
MELO3C009092.2	-1.173672745	0.00230442	down	Protein_LURP-one-related_17
MELO3C009097.2	1.050994793	1.96E-26	up	WRKY_transcription_factor
MELO3C009125.2	1.309909223	6.23E-32	up	Methyltransferase
MELO3C009141.2	-1.521877674	1.72E-21	down	PEBP_(Phosphatidylethanolamine-
MELO3C009143.2	1.172707521	4.67E-08	up	Receptor-like_protein
MELO3C009241.2	-1.014640086	0.000928597	down	High-light-induced_protein_chloropla

MELO3C009288.2	-1.092762843	2.95E-15	down	exocyst_complex_component_EXO70B1-like
MELO3C009300.2	-2.0144964	3.01E-06	down	Unknown_protein
MELO3C009330.2	-2.121936498	9.19E-24	down	Rna_exonuclease_3
MELO3C009372.2	1.942592119	1.05E-43	up	Protein_nuclear_fusion_defective_4
MELO3C009377.2	-1.061069614	1.37E-08	down	aquaporin_TIP1-1-like
MELO3C009383.2	-1.839828465	1.72E-35	down	Thaumatin-like_protein_1a
MELO3C009389.2	-1.295621692	1.53E-35	down	Glycosyltransferase
MELO3C009390.2	-1.199644238	3.77E-10	down	Glycosyltransferase
MELO3C009391.2	-1.620862786	9.78E-31	down	Glycosyltransferase
MELO3C009441.2	2.35867578	2.10E-11	up	ethylene-responsive_transcription_fa
MELO3C009442.2	-6.056988487	0.001980325	down	C-repeat_binding_factor_2
MELO3C009444.2	1.076219328	0.003565285	up	growth-regulating_factor_3
MELO3C009530.2	1.331672738	6.50E-26	up	YLS9
MELO3C009531.2	2.370179332	2.26E-79	up	YLS9
MELO3C009543.2	1.344571395	5.46E-12	up	RING-type_E3_ubiquitin_transfer
MELO3C009572.2	-1.091910725	6.37E-19	down	ABC_transporter_family_protein
MELO3C009596.2	2.039248901	1.31E-29	up	Kelch_repeat-containing_F-
MELO3C009631.2	-2.076628955	0.000162054	down	Seed_maturation_protein
MELO3C009665.2	1.04490816	1.09E-10	up	LOW_QUALITY_PROTEIN:_LRR_receptor-
MELO3C009674.2	-1.305170271	1.61E-21	down	Beta-glucosidase,_putative
MELO3C009678.2	1.337091699	1.48E-17	up	transcription_factor_MYB44
MELO3C009681.2	1.908027726	1.22E-23	up	At3g57450
MELO3C009686.2	1.217561917	1.71E-25	up	Pleiotropic_drug_resistance_ABC_transporter
MELO3C009736.2	-2.414384947	3.12E-62	down	protein_NRT1/_PTR_FAMILY_3.1-like
MELO3C009869.2	-1.428404156	1.02E-19	down	Amino_acid_transporter,_putative
MELO3C009886.2	1.178923291	1.66E-25	up	plant_cysteine_oxidase_2
MELO3C009958.2	-6.199619053	0	down	Carbonic_anhydrase
MELO3C009988.2	2.016771269	1.64E-08	up	Oleosin
MELO3C009989.2	4.108917199	1.33E-33	up	LOB_domain-containing_protein_25
MELO3C010045.2	-1.156984344	4.14E-22	down	Phosphoglycolate_phosphatase-like_protein
MELO3C010114.2	1.930949663	5.43E-15	up	germin-like_protein_subfamily_1_
MELO3C010177.2	1.264391172	7.37E-08	up	WAT1-related_protein
MELO3C010178.2	1.336969367	3.43E-05	up	WAT1-related_protein
MELO3C010185.2	1.087500517	2.00E-21	up	Glycine-rich_protein
MELO3C010186.2	2.106858924	1.27E-15	up	UDP-glucuronate_4-epimerase_1-like
MELO3C010208.2	1.138424716	3.52E-08	up	Unknown_protein
MELO3C010210.2	2.064070407	2.63E-64	up	Unknown_protein

MELO3C010212.2	3.582676069	3.96E-51	up	hepatoma-derived_growth_factor-
MELO3C010249.2	1.302419991	6.00E-27	up	chitotriosidase-1-like
MELO3C010250.2	1.250632556	0.010324676	up	acidic_mammalian_chitinase-like
MELO3C010262.2	2.160464597	8.00E-53	up	NHL_domain-containing_protein,_putati
MELO3C010272.2	-3.124335725	1.38E-12	down	Flavin-containing_monooxygenas
MELO3C010284.2	-1.226870887	0.002490315	down	Unknown_protein
MELO3C010312.2	-1.698119994	0.000308133	down	36.4_kDa_proline-rich_protein
MELO3C010318.2	-1.491369167	2.95E-16	down	Clathrin_assembly_protein,_putative
MELO3C010323.2	1.612608796	2.74E-08	up	umecyanin-like
MELO3C010335.2	3.14890892	1.81E-09	up	EIN3-binding_F-box_1-like_protein
MELO3C010425.2	1.13439396	4.87E-24	up	ethylene-responsive_transcription_fa
MELO3C010431.2	-1.135538866	3.42E-32	down	Serine_hydroxymethyltransferase
MELO3C010466.2	1.403500629	4.46E-13	up	ethylene-responsive_transcription_fa
MELO3C010504.2	-1.032675486	5.41E-05	down	At1g77400
MELO3C010532.2	-1.051282658	2.40E-23	down	Structural_constituent_of_ribosome,_putative
MELO3C010591.2	2.486008143	2.05E-12	up	rRNA_N-glycosidase
MELO3C010608.2	-1.175673843	1.49E-30	down	Photosystem_II_22_kDa_family_protein
MELO3C010623.2	1.036318287	9.92E-20	up	IAA-amino_acid_hydrolase_ILR
MELO3C010668.2	1.780208221	5.43E-06	up	polygalacturonase_At1g48100-like
MELO3C010673.2	-2.688399441	4.05E-12	down	Transmembrane_protein
MELO3C010690.2	1.355735645	0.006582546	up	UDP-galactose:fucoside_alpha-
MELO3C010706.2	-1.508502471	1.53E-19	down	Pectate_lyase
MELO3C010719.2	-1.803080376	0.00067929	down	vinorine_synthase
MELO3C010754.2	-1.380112264	7.11E-33	down	glycine_dehydrogenase_(Decarboxylating),_mitochon
MELO3C010813.2	1.656169129	4.18E-45	up	zinc_finger_protein_ZAT10-like
MELO3C010833.2	1.20413289	1.81E-24	up	MYB_transcription_factor
MELO3C010851.2	-3.892651427	0.001883141	down	Glutamate_receptor
MELO3C010858.2	-1.530777205	0.010404886	down	Abscisic_acid_8'-hydroxylase
MELO3C010859.2	-1.053996954	3.95E-06	down	Chlorophyll_a-b_binding_protein,_chloro
MELO3C010876.2	5.391720583	7.50E-06	up	Calcium-binding_EF-hand
MELO3C010893.2	1.16124056	2.85E-10	up	transcription_factor_MYB44-like
MELO3C010895.2	1.646656332	0.001328085	up	Transmembrane_protein,_putative
MELO3C010911.2	-2.483206146	4.66E-09	down	Structural_constituent_of_ribosome
MELO3C010949.2	1.661788299	0.001054944	up	zinc-finger_homeodomain_prote
MELO3C010969.2	-1.001235436	0.007121276	down	transmembrane_protein_136
MELO3C010984.2	2.781194045	1.99E-08	up	Protein_SHI_RELATED_SEQUENCE_1
MELO3C010994.2	2.535364845	0.003777399	up	Galactose_oxidase,_putative

MELO3C011000.2	1.117743809	0.003178133	up	Cytokinin_riboside_5'-monophosphate_phosphori
MELO3C011027.2	1.033525543	1.59E-06	up	Alpha/beta-hydrolase_superfamily_pro
MELO3C011035.2	1.35501905	2.30E-35	up	Protein_EXORDIUM-like_1
MELO3C011094.2	-1.489039513	0.000302328	down	transcription_factor_bHLH30-like
MELO3C011101.2	2.504695811	7.10E-22	up	mitochondrial_uncoupling_protein_5-like
MELO3C011173.2	-1.034612306	9.43E-22	down	Gamma-glutamylcyclotransferase
MELO3C011187.2	1.4343948	2.65E-05	up	Methionyl-tRNA_synthetase
MELO3C011242.2	-1.478347233	1.99E-28	down	UPF0481_protein_At3g47200
MELO3C011243.2	-5.991911161	0.002314576	down	UPF0481_protein_At3g47200
MELO3C011252.2	1.94980843	2.46E-18	up	EG45-like_domain_containing_pr
MELO3C011254.2	-1.838217645	0.001399528	down	UPF0481_protein_At3g47200
MELO3C011264.2	-1.05344851	0.010721038	down	Histone_acetyltransferase
MELO3C011270.2	1.10777902	1.58E-12	up	Adenine_phosphoribosyltransferase,_putative
MELO3C011329.2	-1.485532302	1.20E-14	down	L-ascorbate_oxidase_homolo
MELO3C011335.2	-2.271363203	0.010623768	down	ornithine_decarboxylase-like
MELO3C011410.2	1.458998112	4.65E-12	up	MADS-box_transcription_factor
MELO3C011443.2	-1.314720353	1.10E-15	down	Glycosyltransferase
MELO3C011474.2	1.801568159	2.35E-13	up	Ankyrin_repeat_family_protein
MELO3C011475.2	1.051265847	3.60E-06	up	Ankyrin_repeat_family_protein
MELO3C011476.2	1.222581961	0.003950647	up	Ankyrin_repeat_family_protein
MELO3C011478.2	-2.298189701	2.81E-44	down	Ankyrin_repeat_family_protein
MELO3C011604.2	-1.100760861	0.003026558	down	transcription_factor_MYB108-like
MELO3C011816.2	1.475760914	1.12E-35	up	3'-N-debenzoyl-2'-deoxytaxol_N-
MELO3C011921.2	1.500648946	6.11E-27	up	Myosin_heavy_chain_kinase_B
MELO3C011927.2	-2.37846884	0.000342949	down	Cytochrome_P450
MELO3C011928.2	-2.402345925	2.78E-49	down	cytochrome_P450_71B19-like
MELO3C011944.2	1.072921721	2.85E-21	up	Beta-1,4-mannosyl-glycoprotein_4-beta-N-
MELO3C011958.2	1.191965017	0.00038564	up	Cyclin-dependent_protein_kinase_
MELO3C011971.2	1.122512438	0.00657797	up	Heme-binding_protein_2
MELO3C011979.2	1.13409715	1.05E-17	up	homeobox_protein_knotted-1-like_6
MELO3C011987.2	1.807248116	1.16E-07	up	Polygalacturonase
MELO3C011991.2	-4.831079784	9.25E-92	down	Hexosyltransferase
MELO3C011995.2	1.061516956	6.96E-05	up	Protein_kinase_family_protein
MELO3C011996.2	1.3935875	1.77E-08	up	At1g70420/F17O7_4
MELO3C012015.2	-2.355183524	6.53E-64	down	WAT1-related_protein
MELO3C012016.2	1.216876921	1.87E-30	up	MLP-like_protein_423
MELO3C012034.2	1.335201829	4.75E-17	up	Hexosyltransferase

MELO3C012051.2	1.065893814	1.31E-20	up	Actin_cross-linking_protein
MELO3C012055.2	-3.818002459	1.52E-47	down	NRT1/PTR_family_protein_2.2
MELO3C012065.2	1.278402835	2.29E-12	up	protein_NRT1/_PTR_FAMILY_4.6
MELO3C012107.2	-1.030036329	1.33E-06	down	Mads_box_protein,_putative
MELO3C012108.2	-1.298659216	9.73E-17	down	Expansin
MELO3C012115.2	-1.029323765	0.001914518	down	Histone-lysine_N-methyltransferase_SMYD3
MELO3C012127.2	1.293683931	1.97E-09	up	protein_argonaute_7
MELO3C012135.2	-1.435001871	1.09E-24	down	Glycosyltransferase
MELO3C012162.2	1.011974949	0.001526004	up	BnaC07g22510D_protein
MELO3C012209.2	-1.402802606	9.08E-45	down	Protein_CHUP1,_chloroplastic
MELO3C012252.2	-1.290179565	8.98E-45	down	Ribulose_bisphosphate_carboxylase_small_chain
MELO3C012263.2	-1.234020296	3.19E-10	down	zinc_finger_protein_6
MELO3C012301.2	1.1451752	7.85E-21	up	Ribose-5-phosphate_isomerase_A
MELO3C012323.2	-1.201588309	4.00E-15	down	3-oxo-5-alpha-steroid_4-dehydrogenase_2-like
MELO3C012325.2	-3.241290248	2.95E-07	down	3-oxo-5-alpha-steroid_4-dehydrogenase_1-like
MELO3C012339.2	-1.033645994	8.97E-10	down	Nuclear_transport_factor_2_(NTF2)_family_protein
MELO3C012348.2	-1.04102052	2.83E-12	down	Serine/threonine-protein_kinase_STE20
MELO3C012352.2	-1.441449249	2.18E-11	down	germin-like_protein_subfamily_3_
MELO3C012365.2	2.084224444	1.54E-37	up	Trihelix_transcription_factor_GT-2
MELO3C012382.2	-1.649671423	4.50E-22	down	non-symbiotic_hemoglobin_2
MELO3C012397.2	1.017009671	0.009199021	up	Protein_nuclear_fusion_defective_4
MELO3C012494.2	-1.02718233	2.66E-15	down	Proline-rich_family_protein
MELO3C012542.2	-2.175531093	1.95E-11	down	BURP_domain_protein_RD22
MELO3C012550.2	-1.76112799	2.61E-07	down	polyol_transporter_5-like
MELO3C012553.2	-1.556837471	7.59E-11	down	polyol_transporter_5-like
MELO3C012556.2	2.473136842	1.48E-06	up	LOW_QUALITY_PROTEIN:_putative_polyol_transp
MELO3C012619.2	-1.042294836	4.31E-05	down	RNA_polymerase_II_elongation_factor_ELL3_isofor
MELO3C012626.2	-1.25377565	6.20E-05	down	Unknown_protein
MELO3C012630.2	1.782500301	1.60E-53	up	cysteine-rich_and_transmembrane_
MELO3C012712.2	-1.862960606	4.79E-18	down	NAD(P)H-quinone_oxidoreductase_s
MELO3C012724.2	1.030672791	0.000249894	up	Lipid_transfer_protein
MELO3C012873.2	-2.295685483	4.92E-09	down	NAC_domain_protein,
MELO3C012885.2	-1.044362977	1.05E-11	down	Patellin-like_protein
MELO3C012890.2	1.324651514	2.50E-08	up	Myb-related_transcription_factor
MELO3C012920.2	1.096920587	2.05E-08	up	GDSL_esterase/lipase_At1g09390-like
MELO3C012926.2	2.152290157	3.28E-05	up	transcription_factor_MYB108
MELO3C012936.2	-3.166279759	6.82E-15	down	boron_transporter_4-like

MELO3C012966.2	1.123341921	4.56E-10	up	BnaC05g50840D_protein
MELO3C012987.2	-1.54997455	1.82E-18	down	Avr9/Cf-9_rapidly_elicited_protein
MELO3C012995.2	-1.050430825	5.18E-06	down	1-aminocyclopropane-1-carboxylate_oxidase_3-like
MELO3C013003.2	1.254089646	2.43E-21	up	Auxin-responsive_protein
MELO3C013014.2	-1.321870012	7.15E-15	down	Inositol_oxygenase
MELO3C013066.2	1.296720624	1.27E-15	up	Ankyrin_repeat-containing_protein
MELO3C013103.2	1.025818285	3.95E-15	up	Lipid_transfer_protein
MELO3C013150.2	2.074699475	1.30E-14	up	mitogen-activated_protein_kinase_k
MELO3C013159.2	1.570778134	0.000983256	up	casein_kinase_II_subunit_beta-2
MELO3C013203.2	-1.339504993	1.01E-28	down	Fructose-bisphosphate_aldolase
MELO3C013207.2	1.437014258	0.009911699	up	Heavy_metal-associated_isoprenylated_p
MELO3C013246.2	1.074769033	2.35E-21	up	Glutamate_receptor
MELO3C013315.2	-2.444821985	2.96E-09	down	O-methyltransferase,_putative
MELO3C013350.2	-1.021581451	3.67E-28	down	D-3-phosphoglycerate_dehydro
MELO3C013379.2	-2.487254644	7.04E-30	down	Neutral/alkaline_invertase
MELO3C013384.2	2.214278308	9.07E-14	up	auxin-responsive_protein_SAUR
MELO3C013402.2	-1.198109298	5.83E-05	down	Unknown_protein
MELO3C013449.2	1.871449857	6.24E-27	up	Isoflavone_reductase_like
MELO3C013476.2	-1.018998074	1.74E-23	down	phototropin-2
MELO3C013562.2	-1.277994135	2.67E-27	down	Flavin-containing_monooxygenas
MELO3C013566.2	1.447268981	1.91E-05	up	Indole-3-acetic_acid-amido_synthetase_GH3.3
MELO3C013592.2	-1.155248651	1.35E-05	down	protochlorophyllide_reductase
MELO3C013623.2	1.011428375	1.92E-09	up	F21B7.33
MELO3C013626.2	-1.299389652	2.79E-19	down	Sulfate/thiosulfate_import_ATP-
MELO3C013644.2	1.213181203	5.33E-07	up	Cytokinin_riboside_5'-monophosphate_phosphori
MELO3C013670.2	1.988029484	3.62E-13	up	exocyst_complex_component_EXO70B1-like
MELO3C013679.2	2.308478006	1.87E-32	up	zinc_finger_protein_ZAT11
MELO3C013719.2	1.10753686	2.53E-11	up	Dirigent_protein
MELO3C013763.2	-2.515347599	0.001951637	down	Thaumatin-like_protein_1
MELO3C013772.2	1.329371249	6.80E-21	up	transcription_factor_MYC3-like
MELO3C013791.2	-1.405276983	0.001389084	down	DUF761_domain_protein
MELO3C013845.2	7.255804301	3.23E-74	up	glycine-rich_cell_wall_structural_p
MELO3C013868.2	-2.968192553	1.39E-91	down	Cytochrome_P450_family_ent-
MELO3C013894.2	-2.319710425	0.004502689	down	Unknown_protein
MELO3C013895.2	1.068177476	2.46E-29	up	Bifunctional_inhibitor/plant_lipid_transfer_protein/see
MELO3C013916.2	1.273517446	1.72E-29	up	ethylene-responsive_transcription_fa
MELO3C013917.2	1.68507962	1.70E-17	up	ethylene-responsive_transcription_fa

MELO3C013952.2	-5.004436712	6.13E-50	down	14_kDa_proline-rich_protein_dc2.15
MELO3C013954.2	1.613118667	4.16E-24	up	14_kDa_proline-rich_protein_dc2.15
MELO3C013956.2	-2.426639244	0.000133871	down	14_kDa_proline-rich_protein_DC2.15
MELO3C013960.2	1.061900902	8.11E-15	up	protein_NRT1/_PTR_FAMILY_6.3-like
MELO3C013962.2	1.04567199	0.003116321	up	Adenine_phosphoribosyltransferase,_putative
MELO3C014026.2	-1.431936646	0.000263034	down	bis(5'-adenosyl)-triphosphatase-like
MELO3C014047.2	-3.836377217	6.19E-119	down	(-)-germacrene_D_synthase-
MELO3C014132.2	-1.128445524	1.04E-09	down	Transmembrane_protein
MELO3C014166.2	1.127930886	0.004160902	up	cytochrome_P450_CYP736A12-like
MELO3C014190.2	-1.324017509	5.29E-06	down	DUF4408_domain_protein
MELO3C014218.2	2.742812401	6.21E-12	up	Unknown_protein
MELO3C014222.2	-1.861095699	0.001952873	down	phenylalanine_ammonia-lyase-like
MELO3C014247.2	1.147860619	0.00257713	up	Methyltransferase_type_11
MELO3C014257.2	1.219448764	0.000212045	up	Arabidopsis_thaliana_genomic_DNA,_chromosome_5
MELO3C014261.2	-1.42870177	1.75E-15	down	AP2-like_ethylene-responsive_transcription_fa
MELO3C014266.2	1.228258669	1.54E-18	up	Auxin-responsive_protein
MELO3C014279.2	-1.048813383	0.001951694	down	Calmodulin,_putative
MELO3C014282.2	-1.030508521	4.41E-18	down	1,2-dihydroxy-3-keto-5-methylthiopentene_dioxyge
MELO3C014284.2	2.355469339	0.000284284	up	Epidermal_patterning_factor-like_protein
MELO3C014290.2	1.688628198	1.56E-20	up	Protein_IQ-DOMAIN_31
MELO3C014360.2	2.682399814	0.002074825	up	Glycosyltransferase
MELO3C014367.2	-1.33137033	0.000649042	down	ABC1_family_protein
MELO3C014376.2	-1.063081699	1.58E-05	down	elongation_of_fatty_acids_protein_3-like
MELO3C014419.2	1.265109988	6.91E-06	up	Unknown_protein
MELO3C014427.2	-1.331223099	0.003141076	down	RING-H2_finger_protein_ATL40
MELO3C014437.2	1.066701944	1.31E-17	up	1-aminocyclopropane-1-carboxylate_oxidase_1
MELO3C014465.2	2.023361291	4.97E-52	up	Xyloglucan_endotransglucosylase/hydrolase
MELO3C014466.2	1.789953752	1.04E-38	up	Xyloglucan_endotransglucosylase/hydrolase
MELO3C014468.2	1.086226848	4.84E-08	up	Xyloglucan_endotransglucosylase/hydrolase
MELO3C014469.2	1.280624816	4.38E-14	up	Xyloglucan_endotransglucosylase/hydrolase
MELO3C014482.2	-1.035425206	1.76E-26	down	Lipoxygenase
MELO3C014485.2	-1.140714137	2.05E-05	down	sugar_transport_protein_13
MELO3C014504.2	1.213469546	1.72E-06	up	Pollen_Ole_e_1_allergen_and_extensin_family_protei
MELO3C014505.2	1.1378365	6.26E-06	up	NAC_domain-containing_protein_90
MELO3C014507.2	1.311981474	2.27E-13	up	F-box_family_protein
MELO3C014512.2	2.417793758	7.69E-71	up	Ribonuclease_CAF1
MELO3C014516.2	-1.024762632	0.000761339	down	NDR1/HIN1-like_protein_12

MELO3C014527.2	1.92204755	0.003329602	up	YABBY_protein
MELO3C014564.2	1.908924393	5.91E-31	up	Protein_LATERAL_ROOT_PRIMORDIUM_1
MELO3C014568.2	-2.007706776	1.99E-87	down	Alanine:glyoxylate_aminotransferase
MELO3C014593.2	-1.374973712	3.54E-11	down	Chaperonin-like_RbcX_protein_2,_chl
MELO3C014616.2	-1.484801472	6.51E-05	down	At5g12930
MELO3C014627.2	-2.821132498	3.21E-66	down	Lipoxygenase
MELO3C014630.2	-1.260958765	5.62E-32	down	Lipoxygenase
MELO3C014652.2	1.789126352	2.52E-15	up	Peroxidase
MELO3C014656.2	1.349826351	9.33E-47	up	Peroxidase
MELO3C014667.2	-1.013820043	8.65E-13	down	Dirigent_protein
MELO3C014686.2	-1.002532379	0.000225574	down	IQ_domain-containing_protein_IQM2
MELO3C014728.2	-1.131195376	2.02E-25	down	phosphoglucan_phosphatase_LSF1,_chloroplastic-like
MELO3C014767.2	-1.744045801	1.07E-55	down	Chalcone_synthase
MELO3C014803.2	-1.595992397	3.17E-15	down	Phosphate_carrier,_mitochondrial
MELO3C014818.2	-1.428895838	2.00E-05	down	Cystinosin-like_protein
MELO3C014827.2	-1.334058199	5.05E-05	down	Class_I_heat_shock_protein
MELO3C014833.2	1.212089086	6.37E-11	up	Glycosyltransferase
MELO3C014872.2	1.127787555	6.68E-21	up	Lipase
MELO3C014907.2	1.467490595	1.12E-38	up	Pollen_Ole_e_1_allergen_and_extensin_family_protei
MELO3C015016.2	-3.863145699	9.85E-09	down	MLP-like_protein_328
MELO3C015021.2	-2.121937599	0.003287862	down	MLP-like_protein_329
MELO3C015051.2	-1.68277001	0.005907802	down	RING-type_E3_ubiquitin_transfer
MELO3C015057.2	1.130574095	0.000233325	up	RING-type_E3_ubiquitin_transfer
MELO3C015058.2	-1.694650391	2.40E-10	down	Cytochrome_P450
MELO3C015118.2	-1.084472952	3.73E-11	down	Unknown_protein
MELO3C015143.2	2.527027599	0.006234889	up	Cytochrome_P450
MELO3C015183.2	2.802782407	3.45E-22	up	phospholipase_A1-Igamma3,_chloroplastic
MELO3C015214.2	-1.438693233	7.44E-18	down	beta-glucosidase_24
MELO3C015221.2	-4.161101706	1.15E-46	down	cyanogenic_beta-glucosidase-like
MELO3C015266.2	2.097327073	4.21E-17	up	calmodulin-binding_protein_60_C-
MELO3C015302.2	1.106340995	5.50E-14	up	Hypoxia-responsive_family_protein
MELO3C015341.2	1.164850089	2.60E-16	up	transcription_factor_bHLH94-like
MELO3C015359.2	-1.057595931	1.98E-15	down	histidine-containing_phosphotransfe
MELO3C015388.2	-1.354345447	1.69E-11	down	cytochrome_P450_CYP72A219-like
MELO3C015409.2	1.282200259	1.32E-19	up	ABC_transporter_B_family_protein
MELO3C015414.2	1.048487619	0.000151906	up	receptor-like_protein_kinase_HSL1
MELO3C015418.2	-1.236538475	2.56E-08	down	protein_IQ-DOMAIN_14-like

MELO3C015468.2	-2.778198779	7.15E-28	down	GATA_transcription_factor_21
MELO3C015469.2	1.219973213	5.34E-20	up	beta-galactosidase-like
MELO3C015479.2	3.15375427	3.22E-06	up	cytokinin_dehydrogenase_4
MELO3C015511.2	-1.469093772	1.16E-24	down	Cytochrome_P450,_putative
MELO3C015513.2	1.119533771	0.00505308	up	growth-regulating_factor_4
MELO3C015524.2	1.095873631	7.41E-05	up	Receptor-like_kinase
MELO3C015536.2	-1.173377339	2.04E-10	down	Chlorophyll_a-b_binding_protein,_chloro
MELO3C015543.2	3.347151426	9.25E-58	up	ethylene-responsive_transcription_fa
MELO3C015786.2	4.143374982	0.000279073	up	Nuclear/nucleolar_GTPase_2
MELO3C015852.2	1.215620853	8.40E-18	up	adenylate_isopentenyltransferase_3,_chloroplastic
MELO3C015888.2	1.032616257	3.69E-05	up	Protein_TIC_214
MELO3C015896.2	-1.45828902	0.006632788	down	Transmembrane_protein
MELO3C015930.2	1.234781573	5.53E-20	up	Translation_initiation_factor_IF-2
MELO3C015941.2	1.703150888	7.45E-15	up	Long_cell-linked_locus_protein,_puta
MELO3C015943.2	1.185762305	1.01E-07	up	zinc_finger_protein_6-like
MELO3C016034.2	1.090035904	0.010090755	up	glutathione_S-transferase_F13
MELO3C016060.2	1.983226597	2.29E-52	up	E3_ubiquitin-protein_ligase_MIEL1-like
MELO3C016109.2	-1.031953866	4.72E-05	down	Protein_trichome_birefringence
MELO3C016167.2	-2.091257166	4.00E-54	down	Glutathione_S-transferase
MELO3C016172.2	1.437301476	2.79E-05	up	serine/threonine-protein_kinase_WAG1
MELO3C016219.2	1.570310781	8.08E-20	up	abscisic_acid_8'-hydroxylase_1
MELO3C016224.2	-1.119108296	1.04E-27	down	9-cis-epoxycarotenoid_dioxygen
MELO3C016231.2	1.221236028	9.36E-05	up	VQ_motif-containing_protein_22
MELO3C016268.2	-2.105318502	4.10E-14	down	cellulose_synthase-like_protein_G3
MELO3C016340.2	1.074186968	5.64E-06	up	1-aminocyclopropane-1-carboxylate_synthase
MELO3C016359.2	2.406594635	1.05E-73	up	Protein_SPIRAL1
MELO3C016402.2	2.407945506	2.75E-44	up	dehydrin_DHN1
MELO3C016426.2	-1.063286885	1.13E-21	down	ferric_reduction_oxidase_7,_chloroplastic
MELO3C016444.2	-1.410002298	2.03E-06	down	NAC_domain-containing_protein_55
MELO3C016569.2	1.967007921	1.14E-42	up	calcium-binding_protein_PBP1
MELO3C016587.2	-5.579078225	0.009340997	down	Sesquiterpene_synthase_Tps1
MELO3C016588.2	-2.166429908	9.40E-62	down	(+)-gamma-cadinene_synthase
MELO3C016616.2	2.499278635	6.38E-10	up	Indole-3-acetic_acid-amido_synthetase_GH3.3
MELO3C016619.2	2.013220298	3.23E-67	up	Transcription_factor_bHLH123_family
MELO3C016674.2	-1.191455608	3.30E-05	down	cyclin-SDS-like_isoform_X1
MELO3C016683.2	-1.258154943	1.40E-08	down	Glucan_endo-1,3-beta-glucosidase
MELO3C016685.2	-1.119450959	9.66E-17	down	cytochrome_P450_CYP72A219-like

MELO3C016686.2	-1.234291725	2.77E-28	down	cytochrome_P450_CYP72 A219-like
MELO3C016687.2	-1.097410159	6.34E-06	down	cytochrome_P450_CYP72 A219-like
MELO3C016712.2	-1.057846403	5.92E-06	down	Stem-specific_protein_TSJT1
MELO3C016752.2	1.242805957	0.000446746	up	abscisic_acid_8'-hydroxylase_4-like
MELO3C016768.2	-1.829642298	2.03E-11	down	Transferase_family_protein
MELO3C016769.2	-4.231745451	1.99E-14	down	Transferase_family_protein
MELO3C016770.2	-1.916662277	0.00209776	down	Transferase
MELO3C016773.2	-1.229107495	3.52E-23	down	Transferase
MELO3C016808.2	1.424944505	4.50E-19	up	LOB_domain-containing_protein_39
MELO3C016809.2	2.192161309	3.36E-80	up	zinc_finger_protein_ZAT1 0-like
MELO3C016830.2	-2.471447873	1.99E-17	down	serine_carboxypeptidase-like_13
MELO3C016840.2	-1.428607452	1.42E-07	down	AP2-like_ethylene-responsive_transcription_fa
MELO3C016841.2	1.118893292	3.86E-16	up	transcription_factor_bHLH 74-like
MELO3C016842.2	1.098772547	0.00090883	up	xyloglucan_glycosyltransfe rase_4
MELO3C016848.2	1.011240481	4.74E-10	up	Homeobox-leucine_zipper_HAT22-
MELO3C016849.2	1.038382819	1.84E-09	up	Homeobox-leucine_zipper_protein
MELO3C016920.2	2.290523143	1.28E-41	up	Receptor-like_protein_kinase
MELO3C016932.2	-3.231524818	1.48E-130	down	Eukaryotic_initiation_facto r_4F_subunit_p150_isofor
MELO3C016976.2	-1.559549782	3.67E-10	down	zinc_finger_protein_1-like
MELO3C016980.2	-1.399918969	3.75E-08	down	ethylene-responsive_transcription_fa
MELO3C016989.2	1.55550576	0.002338569	up	DNA_ligase
MELO3C016996.2	-1.530811045	4.19E-20	down	Transmembrane_protein,_p utative
MELO3C017013.2	1.143538533	4.16E-08	up	DNA-directed_RNA_polymerase
MELO3C017023.2	-1.069540014	7.54E-28	down	Catalase
MELO3C017070.2	1.690201396	4.12E-21	up	wall-associated_receptor_kinase
MELO3C017071.2	1.093022696	3.56E-10	up	Cysteine/Histidine-rich_C1_domain_family_p
MELO3C017072.2	1.310677611	2.40E-07	up	Poly_polymerase
MELO3C017084.2	-2.148955391	0.002757397	down	Unknown_protein
MELO3C017124.2	2.060773497	6.98E-25	up	CDP-diacylglycerol--glycerol-3-phosphate_3-
MELO3C017146.2	-1.030722943	1.86E-08	down	protein_NUCLEAR_FUSI ON_DEFECTIVE_4
MELO3C017171.2	-1.020747819	3.98E-08	down	Zinc_finger_family_protein
MELO3C017182.2	-1.005438007	1.11E-09	down	Photosynthetic_NDH_subc omplex_B_4
MELO3C017187.2	1.161188772	5.30E-06	up	Pectinesterase_inhibitor
MELO3C017246.2	1.86676963	4.33E-09	up	Adenine_nucleotide_alpha _hydrolases-
MELO3C017283.2	2.502036402	9.35E-11	up	Transmembrane_protein,_p utative
MELO3C017297.2	-1.023711024	1.11E-11	down	protein_ASPARTIC_PROT EASE_IN_GUARD_CELL
MELO3C017305.2	1.509351265	2.62E-30	up	LOB_domain-containing_protein_41

MELO3C017315.2	1.742858109	3.74E-19	up	transcription_factor_MYB 24-like
MELO3C017415.2	1.035067101	5.83E-15	up	WRKY_transcription_facto r,_putative
MELO3C017424.2	1.172410922	5.22E-17	up	transcription_factor_bHLH 35
MELO3C017469.2	2.674716783	2.96E-28	up	DUF761_domain_protein
MELO3C017478.2	1.953224952	7.39E-57	up	Xyloglucan_endotransgluc osylase/hydrolase
MELO3C017479.2	1.295978373	0.000213914	up	Xyloglucan_endotransgluc osylase/hydrolase
MELO3C017507.2	-1.888860977	4.72E-09	down	glutamate_receptor_2.7- like
MELO3C017557.2	-2.546809458	0.007349108	down	proline-rich_receptor- like_protein_kinase_PERK
MELO3C017575.2	-1.164658542	0.004471153	down	BnaC01g00560D_protein
MELO3C017582.2	-1.125036155	1.30E-08	down	Protein_MARD1
MELO3C017596.2	1.073452943	5.01E-07	up	Avr9/Cf- 9_rapidly_elicited_protein,
MELO3C017623.2	1.286750124	4.46E-14	up	Metacaspase-1
MELO3C017645.2	1.149186203	1.04E-06	up	ethylene- responsive_transcription_fa
MELO3C017673.2	-1.856355929	1.98E-17	down	Glycosyltransferase
MELO3C017674.2	1.679978786	9.98E-26	up	Lipid_transfer_protein
MELO3C017689.2	-4.558162763	5.81E-12	down	vinorine_synthase-like
MELO3C017739.2	-1.671080228	4.89E-17	down	Transmembrane_protein,_p utative
MELO3C017810.2	1.088466531	9.70E-05	up	phenylalanine_ammonia- lyase-like
MELO3C017828.2	-4.298409723	0.002731233	down	Potassium_transporter
MELO3C017831.2	3.353561583	4.13E-41	up	aquaporin_NIP6-1
MELO3C017863.2	-2.193686096	1.60E-07	down	SNF1- related_protein_kinase_reg
MELO3C017874.2	-6.669621926	0.00019073	down	palmitoyl- monogalactosyldiacylglyce
MELO3C017906.2	1.29345733	1.63E-20	up	acyl-protein_thioesterase_2
MELO3C017917.2	-2.893082269	9.37E-105	down	Calvin_cycle_protein_CP1 2,_chloroplastic
MELO3C017925.2	-1.256499762	1.03E-06	down	Myb_transcription_factor
MELO3C017940.2	1.580086984	1.60E-35	up	Ethylene- responsive_transcription_fa
MELO3C017945.2	-1.103905026	0.010766643	down	bifunctional_epoxide_hydr olase_2-like
MELO3C017972.2	-1.105737696	0.001961785	down	Gamete_expressed_protein _1,_putative
MELO3C018026.2	-1.012938153	3.00E-17	down	Tubulin_alpha_chain
MELO3C018057.2	-1.011548454	0.001249671	down	proline-rich_receptor- like_protein_kinase_PERK
MELO3C018065.2	-2.585714567	0.004891475	down	BnaA07g05330D_protein
MELO3C018122.2	1.490172638	0.008556678	up	thioredoxin- like_protein_CXXS1
MELO3C018131.2	-1.042624051	2.04E-05	down	Photosynthetic_NDH_subc omplex_L_3
MELO3C018144.2	1.79607502	3.57E-51	up	Scarecrow-like_protein
MELO3C018166.2	1.135603923	2.92E-05	up	Indole-3-acetic_acid- amido_synthetase_GH3.3
MELO3C018185.2	-1.629519005	2.00E-05	down	Dynein_light_chain_family _protein
MELO3C018228.2	1.30464071	1.87E-29	up	Heat_shock_21- like_protein

MELO3C018234.2	1.871329228	1.78E-15	up	BnaA05g24030D_protein
MELO3C018314.2	2.406792212	8.68E-05	up	subtilisin-like_protease_SBT4.14
MELO3C018316.2	2.426974388	9.55E-44	up	subtilisin-like_protease_SBT1.1
MELO3C018380.2	2.864928494	0.000242513	up	LOB_domain-containing_protein_12-like
MELO3C018422.2	-1.63414196	5.33E-40	down	1-aminocyclopropane-1-carboxylate_oxidase_2
MELO3C018423.2	-1.350008775	9.40E-06	down	Salicylic_acid-binding_protein_2
MELO3C018447.2	5.656237433	0.004528903	up	ethylene-responsive_transcription_fa
MELO3C018464.2	-1.229997185	1.18E-17	down	Glycosyltransferase
MELO3C018467.2	-1.041864269	0.001004234	down	Glycosyltransferase
MELO3C018476.2	-1.640831836	1.69E-18	down	cyclin-D3-3
MELO3C018485.2	-1.030164975	2.35E-09	down	22.0_kDa_class_IV_heat_shock_protein
MELO3C018490.2	-1.380094805	2.26E-13	down	Glycosyltransferase
MELO3C018507.2	1.122944155	6.65E-21	up	Protein_phosphatase_2c,_putative
MELO3C018528.2	-2.142871318	2.53E-15	down	Photosystem_I_reaction_center_subunit_N
MELO3C018539.2	1.839580365	3.01E-23	up	pathogenesis-related_protein_1-like
MELO3C018565.2	1.028471132	1.38E-14	up	Glutamyl-tRNA_reductase
MELO3C018598.2	-2.766286882	1.44E-05	down	Hydroxyproline-rich_glycoprotein_family_
MELO3C018599.2	-2.677774216	0.000323503	down	IgA_FC_receptor
MELO3C018600.2	-1.553554979	6.92E-05	down	IgA_FC_receptor
MELO3C018631.2	-2.159633674	9.47E-21	down	7-deoxyloganetin_glucosyltra
MELO3C018632.2	-1.975307792	6.59E-40	down	7-deoxyloganetin_glucosyltra
MELO3C018656.2	-1.207736793	4.31E-10	down	Peroxidase
MELO3C018693.2	-3.304396422	0.001926911	down	O-acyltransferase_WSD1-like
MELO3C018709.2	1.165345155	7.11E-06	up	zinc_finger_CCCH_domain-containing_protein_2-
MELO3C018733.2	1.337008272	5.55E-06	up	MLP_protein
MELO3C018739.2	5.640464	0.005076241	up	GPI-anchored_protein_LOREL
MELO3C018746.2	-1.366193261	0.008673814	down	Late_embryogenesis_abundant_protein
MELO3C018754.2	-1.229854458	2.11E-05	down	CTTNBP_2_amino-terminal-like_protein
MELO3C018768.2	1.342039681	1.17E-27	up	Wound-responsive_family_protein
MELO3C018769.2	1.161295129	5.49E-15	up	Wound-responsive_family_protein
MELO3C018770.2	1.809662936	9.91E-34	up	Wound-responsive_family_protein
MELO3C018784.2	2.101088042	3.08E-34	up	Unknown_protein
MELO3C018796.2	1.586282147	1.54E-08	up	Cysteine-rich_receptor-kinase-like_protein
MELO3C018799.2	2.531514584	2.79E-44	up	Cysteine-rich_receptor-like_kinase
MELO3C018814.2	-1.075676581	1.73E-07	down	Protein_DETOXIFICATION
MELO3C018822.2	-1.167532859	9.59E-05	down	glycine-rich_cell_wall_structural_p
MELO3C018825.2	1.724018903	6.34E-05	up	mitogen-activated_protein_kinase_k

MELO3C018839.2	-1.249884452	1.49E-11	down	Lycopene_beta/epsilon_cyclase
MELO3C018843.2	1.141949197	1.00E-21	up	Cytochrome_b561_and_domon_domain-
MELO3C018862.2	1.315200745	4.68E-13	up	Fasciclin-like_arabinogalactan_prote
MELO3C018877.2	-1.832332642	0.001157692	down	Calcium-binding_EF-hand_family_protein
MELO3C018878.2	-1.212535386	5.15E-26	down	Pathogen-induced_protein_CuPi1
MELO3C018882.2	1.102467347	1.49E-14	up	Unknown_protein
MELO3C018886.2	1.119023833	0.002028339	up	BnaA09g54870D_protein
MELO3C018890.2	-2.701392996	6.93E-08	down	glutamic_acid-rich_protein-like
MELO3C018921.2	1.843195946	2.70E-10	up	Glutamine_dumper,_putative
MELO3C018937.2	-1.01622088	6.91E-20	down	RHOMBOID-like_protein_9,_chloroplast
MELO3C018979.2	1.997311266	0.003168538	up	UNC93-like_protein
MELO3C019099.2	-1.044417059	7.60E-05	down	CDPK-related_kinase_1
MELO3C019168.2	1.135265582	3.89E-23	up	Metacaspase-9
MELO3C019221.2	-7.310791382	1.17E-05	down	cytochrome_P450_81D1-like
MELO3C019231.2	-1.152984891	2.42E-27	down	Zinc_finger,_B-box
MELO3C019234.2	-3.510123434	0.00053736	down	cyclin-U1-1
MELO3C019247.2	2.091545802	3.44E-07	up	CTD_small_phosphatase-like_protein
MELO3C019254.2	-1.474008283	1.24E-13	down	septum-promoting_GTP-binding_protein_1
MELO3C019310.2	-1.185100695	3.15E-28	down	protein_NRT1/_PTR_FAMILY_6.4
MELO3C019326.2	2.113217612	5.23E-39	up	Glutaredoxin
MELO3C019335.2	1.006061423	1.02E-09	up	Unknown_protein
MELO3C019337.2	-1.096481511	0.000233585	down	Two-component_response_regul
MELO3C019384.2	-1.124140753	4.14E-18	down	Metal-nicotianamine_transporter
MELO3C019401.2	-3.976994078	0.001288201	down	NAC_domain_protein,
MELO3C019441.2	-1.195063013	9.99E-19	down	ferredoxin-like
MELO3C019469.2	-1.100938457	3.52E-29	down	glutelin_type-A_2-like
MELO3C019470.2	-2.656234616	6.15E-83	down	glutelin_type-A_2-like
MELO3C019506.2	1.678914556	7.38E-37	up	ethylene-responsive_transcription_fa
MELO3C019552.2	-2.693417725	2.51E-79	down	Neuronal_PAS_domain_protein
MELO3C019611.2	1.320793781	1.40E-09	up	DNA-3-methyladenine_glycosylase
MELO3C019772.2	1.639593698	1.68E-14	up	protein_NRT1/_PTR_FAMILY_4.6-like
MELO3C019796.2	1.000502521	2.32E-21	up	Mannan_endo-1,4-beta-mannosidase
MELO3C019835.2	-1.345114938	5.62E-09	down	Protein_nuclear_fusion_defective_4
MELO3C019992.2	-4.82689474	1.75E-07	down	Hypoxia-responsive_family_protein
MELO3C019994.2	-1.655850995	3.11E-09	down	Peroxidase
MELO3C020131.2	-1.395038088	6.78E-41	down	Potassium_transporter
MELO3C020166.2	1.607743997	1.32E-29	up	LOW_QUALITY_PROTEIN:_probable_WRKY_trans

MELO3C020180.2	-3.949247149	0.010179061	down	Cytochrome_P450
MELO3C020245.2	1.917118267	1.27E-38	up	RING-type_E3_ubiquitin_transfer
MELO3C020264.2	-1.811222318	8.06E-13	down	kirola-like
MELO3C020268.2	-3.051266977	1.04E-11	down	MLP-like_protein_28
MELO3C020289.2	1.249404108	0.000319077	up	Nutrient_reservoir,_putative
MELO3C020306.2	2.481162558	6.60E-05	up	heavy_metal-associated_isoprenylated_p
MELO3C020357.2	-1.571346869	4.56E-26	down	Sucrose-phosphate_synthase
MELO3C020501.2	4.404938252	1.13E-44	up	Peroxidase
MELO3C020516.2	1.524541082	0.000462927	up	Cation/H(+)_antiporter
MELO3C020536.2	1.631740022	6.56E-49	up	Protein_phosphatase_2C
MELO3C020559.2	-1.710774372	0.002844479	down	GDP-mannose_transporter,_puta
MELO3C020660.2	2.226441612	1.11E-09	up	p-hydroxybenzoic_acid_efflu
MELO3C020684.2	1.230102986	0.000251237	up	zinc_finger_protein_CONSTANS-LIKE_3-like
MELO3C020689.2	-6.249100594	0.001085679	down	Respiratory_burst_oxidase-like_protein
MELO3C020699.2	4.100935796	0.003565665	up	Myb_transcription_factor
MELO3C020718.2	1.692991202	6.87E-59	up	Mitogen-activated_protein_kinase
MELO3C020745.2	-1.040118694	1.40E-12	down	BnaC08g28080D_protein
MELO3C020769.2	1.841929229	0.003949616	up	auxin-responsive_protein_SAUR
MELO3C020772.2	-1.444819105	1.24E-12	down	p-loop_nucleoside_triphosph
MELO3C020804.2	1.115396459	0.006495762	up	endo-1,4-beta-xylanase_B
MELO3C020857.2	-1.562315065	4.58E-19	down	F-box_family_protein
MELO3C020862.2	-3.465090337	0.010835804	down	T-box_transcription_factor,_
MELO3C020884.2	1.251995351	2.16E-12	up	HTH-type_transcriptional_regula
MELO3C020907.2	1.986912447	0.000832353	up	GRAS_family_transcription_factor
MELO3C020922.2	-4.014289192	0.00757428	down	B3_domain-containing_protein_At3g18
MELO3C020941.2	-1.017688552	3.50E-25	down	Calcium_uniporter,_mitochondrial
MELO3C020970.2	3.207329169	7.72E-05	up	Root_meristem_growth_factor_6
MELO3C020971.2	1.730790679	1.41E-10	up	ethylene-responsive_transcription_fa
MELO3C020975.2	2.819064149	4.07E-06	up	cytochrome_P450_78A7
MELO3C020982.2	1.809072809	1.13E-32	up	At1g49310
MELO3C020983.2	1.844558315	2.17E-15	up	BURP_domain_protein_RD22
MELO3C021085.2	-1.840617067	4.35E-06	down	Membrane_protein_of_er_body-like_protein
MELO3C021119.2	1.045901478	3.28E-20	up	Cytochrome_b561_and_domon_domain-
MELO3C021131.2	-2.888373246	2.17E-44	down	NAC_domain-containing_protein_21/22-
MELO3C021144.2	1.044932377	3.28E-05	up	squamosa_promoter-binding-like_protein_8
MELO3C021147.2	-3.260444672	1.74E-09	down	14_kDa_proline-rich_protein_DC2.15,_puta
MELO3C021151.2	1.44390673	6.46E-05	up	PLATZ_transcription_factor_family_protein

MELO3C021156.2	-2.569482681	2.96E-17	down	crocetin_glucosyltransferase,_chloroplastic-like
MELO3C021164.2	2.305584836	1.77E-12	up	Glycosyltransferase
MELO3C021178.2	-1.149720508	0.002818203	down	Reticulon-like_protein
MELO3C021231.2	-1.472940215	2.37E-28	down	Non-specific_serine/threonine_p
MELO3C021249.2	-1.534651286	3.20E-46	down	Hexosyltransferase
MELO3C021264.2	-2.183634629	2.99E-12	down	Ubiquitin-conjugating_enzyme,_E2
MELO3C021274.2	-1.087353969	0.006025296	down	Outer_envelope_pore_protein_16-2,_chloroplastic
MELO3C021337.2	5.710116152	0.003997872	up	Protein_SRC2
MELO3C021341.2	-2.810437358	0.007873521	down	Protein_SRC2
MELO3C021386.2	-1.816635867	0.005786997	down	S-adenosylmethionine_decar
MELO3C021426.2	3.778877565	1.75E-20	up	Protein_TERMINAL_FLOWER_1
MELO3C021428.2	-1.927786402	1.10E-38	down	Transmembrane_9_superfamily_member
MELO3C021553.2	-1.096101561	2.21E-14	down	50S_ribosomal_protein_L12,_chloroplastic-like
MELO3C021578.2	3.148210079	5.75E-53	up	LOB_domain-containing_protein_25
MELO3C021600.2	-1.207567328	0.002314056	down	cytochrome_P450_724B1
MELO3C021607.2	-1.053374785	2.38E-10	down	pectate_lyase-like
MELO3C021626.2	1.3559124	1.77E-06	up	protein_kinase_APK1B,_chloroplastic-like
MELO3C021628.2	1.598213425	0.00267061	up	Transmembrane_protein,_putative
MELO3C021682.2	-1.318519997	8.50E-05	down	TNFR/CD27/30/40/95_cysteine-rich_region
MELO3C021697.2	-1.894123834	0.00019005	down	DUF506_family_protein_(DUF506)
MELO3C021708.2	-1.898140115	6.39E-51	down	Ubiquinol_oxidase
MELO3C021744.2	-1.019838987	0.000213848	down	Pentatricopeptide_repeat-containing_protein
MELO3C021811.2	-1.055249864	4.21E-09	down	Ribose-5-phosphate_isomerase_A
MELO3C021821.2	1.139436215	3.66E-10	up	Cellulose_synthase
MELO3C021843.2	-1.616160305	2.31E-11	down	Cytochrome_P450,_putative
MELO3C021846.2	-1.017309771	0.008392996	down	cytochrome_P450_89A2-like
MELO3C021881.2	2.692519076	0.004648585	up	Lectin_receptor_kinase
MELO3C021886.2	-1.100745507	7.46E-24	down	S-norcoclaurine_synthase_1-
MELO3C021901.2	-1.091110611	3.57E-10	down	Alpha/beta_hydrolase-3
MELO3C021904.2	-1.061268414	0.001575624	down	2-hydroxyisoflavanone_dehy
MELO3C021912.2	1.585988667	7.29E-06	up	Peptidyl-prolyl_cis-trans_isomerase
MELO3C021914.2	2.325650394	7.86E-17	up	peroxidase_10-like
MELO3C021918.2	-2.150690804	0.000287751	down	Serine/threonine_protein_kinase
MELO3C021932.2	-2.358933858	8.93E-05	down	Unknown_protein
MELO3C021951.2	-2.771006815	8.95E-05	down	protein_EMBRYO_SAC_DEVELOPMENT_ARRES
MELO3C021982.2	1.03344084	8.91E-13	up	ABC_transporter_B_family_protein
MELO3C021998.2	2.048721091	1.89E-51	up	Calcium_ion_binding_protein

MELO3C021999.2	1.855455557	3.49E-09	up	Expansin_protein
MELO3C022007.2	-1.058147033	0.001173241	down	transmembrane_emp24_domain-
MELO3C022028.2	-2.026928787	4.38E-05	down	Transcription_factor_bHLH151,_putative
MELO3C022044.2	-1.263909339	1.55E-06	down	PI-PLC_X_domain-containing_protein_At5g67
MELO3C022088.2	-1.037892503	4.72E-16	down	Thylakoid_membrane_protein_slr0575
MELO3C022091.2	6.098220778	0.001096757	up	TCP_transcription_factor
MELO3C022098.2	1.900463627	2.77E-14	up	Cytochrome_P450,_putative
MELO3C022133.2	-1.075936304	3.39E-09	down	Beta-1,3-glucanase_2
MELO3C022181.2	1.613874196	1.65E-12	up	ethylene-responsive_transcription_fa
MELO3C022207.2	-1.299075421	1.35E-06	down	Retrovirus-related_Pol_polyprotein_fr
MELO3C022233.2	-1.782492006	2.26E-59	down	transcription_factor_UNE10
MELO3C022234.2	1.37539561	0.001358365	up	cyclin-U2-1
MELO3C022247.2	-2.739154814	3.87E-05	down	cytochrome_b5
MELO3C022251.2	-1.032878093	0.00721629	down	S-adenosyl-L-methionine-dependent_methyltransfera
MELO3C022259.2	-1.958558462	5.11E-46	down	Calcium-binding_family_protein
MELO3C022291.2	2.124629689	3.03E-41	up	Carotenoid_cleavage_dioxygenase
MELO3C022325.2	-2.258660087	2.53E-05	down	proton_pump-interactor_2-like_isoform_X1
MELO3C022341.2	-3.073430644	2.74E-15	down	Bidirectional_sugar_transporter_SWEET
MELO3C022372.2	-3.287448571	9.60E-26	down	Cytochrome_P450
MELO3C022373.2	-3.345659764	7.95E-07	down	3'-N-debenzoyl-2'-deoxytaxol_N-
MELO3C022374.2	-6.243705516	8.58E-05	down	Terpene_cyclase/mutase_family_member
MELO3C022375.2	-1.386503837	1.48E-05	down	Cytochrome_P450
MELO3C022376.2	-3.412036726	2.24E-38	down	Cytochrome_P450
MELO3C022377.2	-3.498338031	6.85E-12	down	Cytochrome_P450
MELO3C022429.2	-2.40949421	2.23E-105	down	Amaranthin-like_lectin
MELO3C022430.2	-1.817058761	2.56E-58	down	Amaranthin-like_lectin
MELO3C022433.2	-2.232044332	2.81E-23	down	Dirigent_protein
MELO3C022436.2	-1.258510792	4.10E-15	down	Amaranthin-like_lectin
MELO3C022493.2	-1.043537339	0.000107275	down	protein_NRT1/_PTR_FAMILY_7.3-like_isoform_X1
MELO3C022516.2	1.9357268	0.006823234	up	MADS-box_transcription_factor
MELO3C022518.2	-1.027288546	0.001647808	down	gibberellin_20_oxidase_1-like
MELO3C022542.2	-1.34893274	0.001581338	down	MYB_transcription_factor-like
MELO3C022568.2	-1.144326663	2.85E-06	down	protein_indeterminate-domain_5,_chloroplastic
MELO3C022574.2	1.078573381	3.30E-17	up	transcription_factor_bHLH111
MELO3C022772.2	-1.1601192	2.00E-18	down	Nitrate_reductase
MELO3C022791.2	-1.722941591	1.64E-15	down	Protein_MARD1
MELO3C022804.2	-1.414891893	0.007308775	down	heavy_metal-associated_isoprenylated_p

MELO3C022820.2	1.397960828	3.95E-38	up	potassium_channel_SKOR
MELO3C022841.2	1.749253224	1.92E-09	up	Serinethreonine-protein_kinase_ctr1
MELO3C022985.2	1.154139262	2.67E-10	up	ethylene-responsive_transcription_fa
MELO3C022987.2	-1.132370458	2.73E-13	down	repetitive_proline-rich_cell_wall_protein_1-
MELO3C022997.2	1.276614433	1.83E-07	up	MYB-related_transcription_factor
MELO3C023032.2	-1.489942719	3.41E-14	down	Cyclopropane-fatty-acyl-phospholipid_synthase_fa
MELO3C023067.2	-1.093042745	5.54E-26	down	Beta-amylase
MELO3C023118.2	1.134375639	0.001829065	up	Myb/SANT-like_DNA-binding_domain_protein
MELO3C023120.2	1.074591317	9.22E-21	up	zinc_finger_AN1_and_C2H2_domain-
MELO3C023161.2	-1.464240634	1.90E-18	down	nuclear_transcription_factor_Y_subunit_A-7-like
MELO3C023234.2	1.358807931	1.42E-06	up	Pectin_lyase-like_superfamily_protein
MELO3C023239.2	1.128015218	1.72E-14	up	GDP-mannose_transporter
MELO3C023275.2	-2.307670175	1.66E-09	down	terpene_synthase_10-like_isoform_X1
MELO3C023288.2	-2.098732655	4.77E-40	down	terpene_synthase_10-like_isoform_X1
MELO3C023310.2	-1.536264667	1.43E-46	down	Glucose-1-phosphate_adenylyltransfer
MELO3C023354.2	-1.08110579	8.55E-25	down	Fructose-bisphosphate_aldolase
MELO3C023360.2	-1.776854701	2.54E-33	down	Thionin-like_protein_2
MELO3C023361.2	1.080045887	3.81E-06	up	Thionin-like_protein_2
MELO3C023391.2	1.021651636	7.89E-18	up	Plant_Tudor-like_protein
MELO3C023392.2	1.867548262	1.80E-05	up	Serine/threonine-protein_kinase
MELO3C023394.2	-1.287250168	2.26E-15	down	(6-4)DNA_photolyase_isofor
MELO3C023401.2	1.615490675	9.66E-17	up	Ran-binding_zinc_finger_protei
MELO3C023431.2	-1.898761691	2.16E-43	down	haloalkane_dehalogenase
MELO3C023436.2	1.206919379	7.30E-24	up	VQ_motif-containing_protein
MELO3C023440.2	2.388209689	0.010344812	up	LRR_receptor-like_serine/threonine-
MELO3C023441.2	2.106641972	2.85E-35	up	Receptor-kinase,_putative
MELO3C023493.2	1.060962825	1.85E-14	up	4-coumarate:CoA_ligase
MELO3C023498.2	1.78787162	0.004074162	up	Protein_UPSTREAM_OF_FLC
MELO3C023522.2	-1.110218813	9.86E-12	down	At4g12735
MELO3C023530.2	3.827775891	9.59E-70	up	cytochrome_P450_705A5-like
MELO3C023532.2	1.532692837	5.68E-29	up	transcription_factor_bHLH130-like_isoform_X2
MELO3C023539.2	-1.103027801	2.41E-12	down	Unknown_protein
MELO3C023573.2	1.085646632	1.91E-18	up	At3g57450
MELO3C023579.2	1.165408386	1.65E-15	up	disease_resistance_protein_RGA2-like_isoform_X1
MELO3C023586.2	-1.337585447	0.000478001	down	Carboxypeptidase
MELO3C023609.2	-1.049499557	4.68E-21	down	TolB_protein-like_protein
MELO3C023615.2	-1.938097833	0.00152013	down	Peroxidase

MELO3C023618.2	-2.143800177	2.61E-69	down	Short-chain_dehydrogenase/redu
MELO3C023619.2	-3.440911157	3.13E-122	down	Short-chain_dehydrogenase/redu
MELO3C023628.2	2.321807021	1.21E-12	up	NIM1-interacting_1,_putative
MELO3C023632.2	2.419942425	0.001123577	up	Pectinesterase
MELO3C023688.2	1.001950561	1.40E-07	up	proline-rich_receptor-like_protein_kinase_PERK
MELO3C023694.2	-2.71553358	2.36E-19	down	Pathogen-related_protein
MELO3C023781.2	-2.341450042	3.14E-11	down	At3g19660
MELO3C023796.2	3.474520878	0.005587235	up	Cytochrome_P450,_putative
MELO3C023802.2	1.783263657	9.20E-57	up	LOB_domain-containing_protein_41
MELO3C023818.2	2.565796569	1.74E-40	up	LOW_QUALITY_PROTEIN:_exocyst_complex_com
MELO3C023822.2	-1.676699235	4.92E-24	down	Caffeoylshikimate_esterase
MELO3C023840.2	1.327379637	1.91E-09	up	zinc_finger_protein_ZAT12-like
MELO3C023879.2	-1.448734502	5.59E-49	down	Ribulose_bisphosphate_carboxylase/oxygenase_activa
MELO3C023892.2	-1.384100717	0.008113228	down	Exostosin_domain-containing_protein/Mur_li
MELO3C023911.2	-1.048459089	3.51E-08	down	cucumisin-like
MELO3C023976.2	3.070561164	1.42E-95	up	Protein_DETOXIFICATION
MELO3C023980.2	2.606980486	4.77E-18	up	polygalacturonase-like
MELO3C023998.2	-1.077985354	2.28E-15	down	Protein_RETICULATA-RELATED_4,_chloroplasti
MELO3C024014.2	1.478719141	2.32E-23	up	Carboxypeptidase
MELO3C024075.2	1.550322893	9.28E-40	up	UPF0496_protein_4-like
MELO3C024163.2	2.026360552	7.51E-08	up	acanthoscurrin-1-like
MELO3C024168.2	-1.134219814	0.004181393	down	phototropin-2-like_isoform_X1
MELO3C024232.2	1.367029937	6.52E-23	up	Transcription_repressor_OFP14
MELO3C024263.2	1.087346206	4.19E-05	up	aquaporin_TIP2-1
MELO3C024264.2	-1.010068246	2.48E-07	down	Photosynthetic_NDH_subcomplex_B_3
MELO3C024267.2	1.353154121	1.38E-05	up	Transmembrane_protein
MELO3C024268.2	-1.111547596	1.08E-05	down	ethylene-responsive_transcription_fa
MELO3C024306.2	-1.136623259	1.18E-13	down	pectinesterase-like
MELO3C024387.2	1.069812698	0.000485714	up	LOB_domain-containing_protein_22
MELO3C024421.2	1.123618601	0.004351069	up	Receptor_protein_kinase,_putative
MELO3C024425.2	1.522105631	1.12E-19	up	Receptor-like_protein_kinase
MELO3C024444.2	-1.024281948	3.48E-09	down	Avr9/Cf-9_rapidly_elicited_protein
MELO3C024449.2	-2.868696282	0.001579027	down	UPF0352_protein_PSHAa1818
MELO3C024493.2	-2.329074065	5.94E-12	down	Glycosyltransferase
MELO3C024498.2	-1.447030188	2.14E-17	down	Unknown_protein
MELO3C024545.2	-1.418730294	7.57E-29	down	Transmembrane_9_superfamily_member
MELO3C024557.2	-1.169646167	1.29E-07	down	BnaAnng07610D_protein

MELO3C024581.2	1.604024377	2.41E-21	up	Harpin_inducing_protein_1-like_9
MELO3C024690.2	-1.271021381	5.92E-05	down	Rac-like_GTP_binding_protein
MELO3C024703.2	-3.171416735	2.15E-06	down	Protein_SPEAR1
MELO3C024724.2	-1.47286536	1.57E-14	down	4-coumarate--CoA_ligase_2
MELO3C024834.2	-3.311759937	0.007413705	down	protein_DMR6-LIKE_OXYGENASE_2-
MELO3C024841.2	-1.761264908	8.36E-06	down	proline-rich_receptor-like_protein_kinase_PERK
MELO3C024842.2	-2.956078117	0.001147019	down	BnaA03g52590D_protein
MELO3C024877.2	-1.484647987	8.21E-05	down	Protein_CHLORORESPIRATORY_REDUCTION_7,
MELO3C025012.2	1.486296968	1.20E-15	up	serine/threonine-protein_kinase-
MELO3C025025.2	-1.996644722	9.73E-32	down	Abscisic_stress_ripening-like_protein
MELO3C025079.2	1.247918707	2.34E-13	up	universal_stress_protein_A-like_protein
MELO3C025120.2	-3.491574268	6.48E-05	down	rRNA_N-glycosidase
MELO3C025149.2	-1.067892532	1.38E-27	down	Sedoheptulose-1,7-bisphosphatase
MELO3C025174.2	1.048680084	2.71E-11	up	Auxin_transporter-like_protein_2
MELO3C025225.2	-1.047227721	5.97E-06	down	high_mobility_group_B_protein_6
MELO3C025274.2	-1.02820955	8.76E-08	down	cell_division_control_protein_2_homolog_C
MELO3C025308.2	1.174825513	1.29E-11	up	auxin-responsive_protein_IAA16-
MELO3C025332.2	1.205342544	5.31E-19	up	Unknown_protein
MELO3C025369.2	-3.865278492	1.71E-32	down	Reactive_Intermediate_Deaminase_A,_chloroplastic
MELO3C025433.2	1.999071294	2.95E-36	up	calcium-binding_protein_PBP1-like
MELO3C025434.2	-1.656516805	1.92E-19	down	gibberellin_2-beta-dioxygenase_8-like
MELO3C025467.2	-1.00492416	3.85E-09	down	photosynthetic_NDH_subunit_of_lumenal_location_1,
MELO3C025473.2	-1.114156956	1.45E-28	down	sulfate_transporter_4.1,_chloroplastic-like
MELO3C025495.2	-1.488051477	0.000259181	down	Hydrolase_family_protein/HAD-superfamily_protein
MELO3C025521.2	1.035848452	4.00E-09	up	Hexosyltransferase
MELO3C025547.2	1.074870907	0.006004878	up	At2g42760
MELO3C025580.2	1.644557503	4.06E-54	up	Ribonuclease_CAF1
MELO3C025608.2	1.953751402	1.11E-51	up	ethylene-responsive_transcription_fa
MELO3C025655.2	1.032632468	3.69E-05	up	Protein_phosphatase_2c,_putative
MELO3C025664.2	-1.095465137	4.54E-07	down	Receptor-like_protein_kinase
MELO3C025688.2	1.92660886	4.29E-06	up	6,7-dimethyl-8-ribityllumazine_synthase
MELO3C025761.2	1.966262767	6.56E-29	up	MLO-like_protein
MELO3C025781.2	1.148703175	8.32E-06	up	Cyclic_nucleotide-gated_ion_channel-
MELO3C025797.2	-1.784743776	6.81E-19	down	Cytochrome_P450
MELO3C025814.2	1.975698213	2.57E-07	up	Ankyrin_repeat-containing_protein
MELO3C025824.2	-1.762557599	0.008725289	down	Sucrose-phosphate_synthase_family
MELO3C025855.2	-2.159248939	8.33E-65	down	Alkyl_transferase

MELO3C025865.2	-1.163799167	3.12E-20	down	Thylakoid_soluble_phosphoprotein
MELO3C025901.2	1.556382184	3.44E-45	up	E3_ubiquitin-protein_ligase_RING1
MELO3C025944.2	-1.062757385	1.60E-22	down	ABC1_family_protein
MELO3C025950.2	1.687530876	0.007963353	up	Nitrile-specifier_protein_5
MELO3C025995.2	-1.426511044	5.70E-11	down	trihelix_transcription_factor_GT-3b
MELO3C026001.2	-1.500973774	6.70E-06	down	Late_embryogenesis_abundant_protein
MELO3C026170.2	-1.74879967	1.34E-12	down	Acyl_carrier_protein
MELO3C026174.2	1.792386851	1.18E-64	up	Sterile_alpha_motif_domain-
MELO3C026212.2	-2.036439456	2.99E-17	down	cytochrome_P450_94B3
MELO3C026214.2	2.998359608	1.71E-41	up	Calcium-binding_protein
MELO3C026215.2	2.711111763	3.69E-16	up	adenine/guanine_permease_AZG2
MELO3C026235.2	-1.617731397	6.55E-37	down	haloacid_dehalogenase-like_hydrolase_domain-
MELO3C026260.2	-3.894782978	1.40E-57	down	cytochrome_P450_71A22-like
MELO3C026269.2	9.01202413	7.86E-10	up	LOB_domain-containing_protein_25
MELO3C026299.2	1.033968779	0.001792476	up	MADS-box_transcription_factor
MELO3C026305.2	2.440338439	0.000692609	up	B12D_protein
MELO3C026318.2	-1.446370085	0.001387384	down	Unknown_protein
MELO3C026367.2	-6.419901621	4.72E-12	down	Glycosyltransferase
MELO3C026378.2	-1.017583631	2.58E-17	down	zinc_transporter_5-like
MELO3C026420.2	-5.488740038	2.40E-42	down	Phosphomannomutase/phosphoglucomutase
MELO3C026423.2	-1.037354508	1.41E-05	down	Chlororespiratory_reduction_3
MELO3C026468.2	-2.183293141	1.20E-47	down	NADPH-dependent_pterin_aldehyde
MELO3C026484.2	-1.0484821	0.000915781	down	Cytochrome_P450_family_protein
MELO3C026485.2	-1.323175344	1.70E-06	down	Cytochrome_P450
MELO3C026488.2	-1.033075104	4.60E-25	down	cytochrome_P450_82A3-like
MELO3C026492.2	-1.174963118	8.42E-22	down	Cytochrome_P450
MELO3C026500.2	1.197252607	3.99E-31	up	Actin_cross-linking_protein
MELO3C026521.2	3.360358893	0.002640374	up	protein_CUP-SHAPED_COTYLEDON_
MELO3C026545.2	2.141926323	0.000243941	up	DNA-directed_RNA_polymerase
MELO3C026558.2	-1.724066322	5.49E-07	down	Glutamate_receptor
MELO3C026575.2	-1.096562448	0.000758259	down	Glycosyltransferase
MELO3C026644.2	-1.044170367	2.59E-05	down	Aspartate_racemase
MELO3C026677.2	3.506327006	1.46E-29	up	Receptor_protein_kinase
MELO3C026703.2	-5.158753025	8.43E-17	down	Protein_SULFUR_DEFICIENCY-INDUCED_1
MELO3C026722.2	-1.526687236	3.10E-57	down	Phosphoribulokinase
MELO3C026740.2	2.39456085	4.73E-67	up	WRKY_transcription_factor
MELO3C026754.2	1.250739057	4.70E-17	up	Respiratory_burst_oxidase-like_protein

MELO3C026784.2	-1.26453798	0.000120957	down	serine/threonine-protein_kinase_BLUS1-
MELO3C026788.2	1.604205609	6.90E-57	up	Zinc_finger_CCCH_domain-containing_protein_29-
MELO3C026808.2	6.787093578	7.67E-05	up	SABATH_methyltransferase_9
MELO3C026811.2	1.302872965	2.60E-32	up	endo-1,4-beta-xylanase_A-like
MELO3C026824.2	2.04584704	2.00E-13	up	cytochrome_P450_714A1-like
MELO3C026889.2	-1.479852167	1.89E-18	down	short-chain_type_dehydrogenase/
MELO3C026908.2	1.48590325	5.20E-52	up	heavy_metal-associated_isoprenylated_p
MELO3C026932.2	1.410215637	4.82E-10	up	WRKY_transcription_factor,_putative
MELO3C026968.2	-1.26484699	1.75E-19	down	Myosin-G_heavy_chain-like_protein
MELO3C026974.2	1.089848416	8.23E-20	up	aspartyl_protease_family_protein_2
MELO3C027015.2	-3.457297897	0.003194779	down	ACT_domain-containing_protein
MELO3C027020.2	-1.095787548	1.23E-25	down	Ubiquinol_oxidase
MELO3C027042.2	2.466587779	9.34E-34	up	Protein_SHI_RELATED_SEQUENCE_1
MELO3C027053.2	-3.528540171	0.010111855	down	Protein_TIC_214
MELO3C027055.2	-2.236990703	0.000988263	down	NAD(P)H-quinone_oxidoreductase_s
MELO3C027102.2	-3.997475077	0.007513026	down	DNA-directed_RNA_polymerase
MELO3C027124.2	-1.047032745	1.78E-18	down	17.5_kDa_class_I_heat_shock_protein
MELO3C027151.2	-3.392930446	0.004905334	down	alcohol_dehydrogenase-like
MELO3C027212.2	-1.065949576	8.07E-08	down	Pre-mRNA-processing-splicing_factor
MELO3C027216.2	1.927521283	2.73E-24	up	Transcription_factor,_putative
MELO3C027288.2	-1.330248387	1.81E-33	down	Threonine_dehydratase
MELO3C027302.2	2.45267256	5.49E-77	up	basic_blue_protein-like
MELO3C027328.2	1.060261158	2.35E-05	up	Carboxypeptidase
MELO3C027346.2	1.844184635	9.35E-27	up	Indole-3-acetic_acid-amido_synthetase_GH3.3
MELO3C027351.2	-1.491770397	0.001331721	down	Photosystem_I_assembly_protein_Ycf3
MELO3C027370.2	-1.71572413	2.61E-05	down	O-methyltransferase,_putative
MELO3C027417.2	-2.430697028	0.005443877	down	NADH_dehydrogenase_subunit_5
MELO3C027566.2	-5.665066512	0.006547141	down	Unknown_protein
MELO3C027617.2	5.710116152	0.003997872	up	Unknown_protein
MELO3C027727.2	-3.858407557	8.59E-53	down	(-)-germacrene_D_synthase-
MELO3C027737.2	-1.327622152	5.87E-10	down	Peroxisome_biogenesis_protein_5
MELO3C027760.2	-1.760127143	0.000616882	down	Pyruvate_kinase
MELO3C027807.2	-3.74513993	8.01E-05	down	Succinate_dehydrogenase_subunit_4
MELO3C027914.2	-1.240814661	2.42E-08	down	protein_NRT1/_PTR_FAMILY_1.2-like
MELO3C027941.2	-2.905727748	0.004714077	down	Orf101b
MELO3C028126.2	-2.029863758	2.63E-05	down	BHLH_transcription_factor
MELO3C028147.2	-3.122304161	0.000317557	down	Photosystem_I_P700_chlorophyll_a_apoprotein_A1

MELO3C028501.2	-3.502621848	1.71E-05	down	30S_ribosomal_protein_S16,_chloroplastic
MELO3C028543.2	-1.052865943	0.003987541	down	Ty3-gypsy_retrotransposon_pro
MELO3C028562.2	-1.952037232	1.11E-05	down	Protein_DETOXIFICATION
MELO3C028590.2	1.498479095	3.59E-05	up	Myb_transcription_factor
MELO3C028593.2	-1.674262063	8.72E-05	down	Protein_DETOXIFICATION
MELO3C028661.2	3.359365424	0.01066349	up	Unknown_protein
MELO3C028700.2	-5.576142626	0.008092146	down	Unknown_protein
MELO3C028718.2	6.780128325	8.04E-05	up	expansin-like_A1
MELO3C029220.2	-2.221769818	0.007429579	down	Unknown_protein
MELO3C029317.2	-1.331374141	2.47E-31	down	L-ascorbate_oxidase
MELO3C029341.2	2.391409491	4.04E-134	up	Harbinger_transposase-derived_nuclease
MELO3C029529.2	-1.275096611	7.31E-15	down	Plant_intracellular_Ras-group-
MELO3C029543.2	-2.251236111	9.41E-07	down	zinc_finger_BED_domain-containing_protein_RICES
MELO3C029682.2	-1.139488024	8.54E-08	down	Transcriptional_corepressor_LEUNIG
MELO3C029736.2	2.55090583	0.009621359	up	Defensin-like_protein_3
MELO3C029738.2	-1.040245767	4.51E-07	down	Terpene_cyclase/mutase_family_member
MELO3C029746.2	-1.836767789	0.0016033	down	UBN2_3_domain-containing_protein
MELO3C029753.2	-1.9803644	1.63E-18	down	Respiratory_burst_oxidase-like_protein
MELO3C029915.2	1.771878367	0.009701485	up	Retrovirus-related_Pol_polyprotein_fr
MELO3C029946.2	-5.915347387	0.002817732	down	Unknown_protein
MELO3C029951.2	1.122803608	6.29E-09	up	Retrotransposon_protein
MELO3C030107.2	2.367000688	2.75E-43	up	mitochondrial_uncoupling_protein_4-like
MELO3C030167.2	1.871218875	2.16E-07	up	Flavonol_synthase
MELO3C030241.2	-1.558318379	0.004926548	down	equilibrative_nucleotide_transporter_3-like
MELO3C030287.2	1.246409151	7.08E-07	up	WRKY_transcription_factor,_putative
MELO3C030308.2	-1.829677043	0.008790932	down	UPF0481_protein_At3g47200
MELO3C030339.2	-1.750042949	0.000155913	down	Phytosulfokines_3-like_protein
MELO3C030358.2	2.602257589	0.008110332	up	Unknown_protein
MELO3C030573.2	2.533790742	3.24E-80	up	Thionin-like_protein_2
MELO3C030668.2	1.052506001	2.84E-07	up	Cinnamoyl-CoA_reductase
MELO3C030719.2	-1.851876171	5.65E-16	down	cytochrome_P450_94A1-like
MELO3C030721.2	-3.341394501	4.44E-10	down	Beta-glucosidase,_putative
MELO3C030722.2	-3.79064125	4.46E-07	down	Beta-glucosidase,_putative
MELO3C030868.2	-1.352729393	8.73E-13	down	GDSL_esterase/lipase_1-like
MELO3C030922.2	-1.063933369	4.35E-10	down	Unknown_protein
MELO3C031003.2	2.034604871	1.54E-35	up	Homogentisate_phytyltransferase
MELO3C031037.2	2.80854761	2.27E-47	up	Unknown_protein

MELO3C031083.2	1.667376084	1.86E-16	up	Protein_LATERAL_ROOT_PRIMORDIUM_1
MELO3C031144.2	-1.267459031	1.17E-05	down	Unknown_protein
MELO3C031342.2	3.257927063	1.17E-54	up	UDP-sugar_transporter-like_protein
MELO3C031450.2	-1.233687549	3.70E-08	down	GDSL_esterase/lipase_At5g22810
MELO3C031571.2	1.490715063	1.36E-25	up	UDP-sugar_transporter_sqv-7-
MELO3C031616.2	2.761999131	0.001845881	up	exopolygalacturonase-like
MELO3C031621.2	-1.884492705	6.42E-21	down	Wall-associated_receptor_kinase
MELO3C031638.2	-1.851078802	0.01086728	down	LOB_domain-containing_protein_12
MELO3C031654.2	-1.147158562	9.93E-05	down	Serinc-domain_containing_serine
MELO3C031734.2	-1.839768811	1.51E-17	down	Bidirectional_sugar_transporter_SWEET
MELO3C031780.2	2.457391099	3.99E-24	up	Unknown_protein
MELO3C031850.2	-2.682961646	0.002002402	down	Unknown_protein
MELO3C031857.2	-1.197094704	0.000191008	down	organic_cation/carnitine_transporter_4-like
MELO3C031893.2	4.147112157	0.003071113	up	protein_disulfide_isomerase-like_1-6
MELO3C031941.2	1.882207104	5.00E-37	up	glycine-rich_cell_wall_structural_p
MELO3C031946.2	2.989293803	2.03E-43	up	basic_blue_protein-like
MELO3C031972.2	-4.735834824	2.58E-05	down	Unknown_protein
MELO3C032143.2	-1.385242729	0.006503612	down	Unknown_protein
MELO3C032257.2	10.12561606	7.08E-12	up	DEAD-box_ATP-dependent_RNA_helicase_
MELO3C032550.2	-2.590910045	1.15E-33	down	Aldo/keto_reductase_family_protein
MELO3C032740.2	-1.069107315	0.00196623	down	Unknown_protein
MELO3C032750.2	-1.018871012	2.14E-06	down	ADP-ribosylation_factor
MELO3C032836.2	2.92010712	1.48E-08	up	Unknown_protein
MELO3C032845.2	2.424983195	8.25E-05	up	Protein_LAZY_1
MELO3C032874.2	-4.409432597	0.001455318	down	Unknown_protein
MELO3C032879.2	-1.429094403	2.62E-05	down	Unknown_protein
MELO3C032882.2	2.800131456	2.24E-17	up	Protein_SRG1
MELO3C032937.2	-1.458212904	6.74E-06	down	glucomannan_4-beta-mannosyltransferase_9-like
MELO3C033082.2	1.5133597	0.002344199	up	Unknown_protein
MELO3C033125.2	1.17183874	3.89E-06	up	Gibberellin_20-oxidase,_putative
MELO3C033198.2	-1.786203528	7.13E-05	down	Glutathione_S-transferase
MELO3C033484.2	5.790839147	0.00291242	up	Unknown_protein
MELO3C033612.2	1.746597333	0.000868863	up	MYB-related_transcription_factor
MELO3C033753.2	-1.251210612	0.006364622	down	Purple_acid_phosphatase
MELO3C033947.2	-1.087021945	0.007148129	down	UDP-glucose:glycoprotein_gluco
MELO3C033988.2	-2.999897567	3.64E-63	down	Unknown_protein
MELO3C034013.2	1.949603044	3.60E-08	up	Myb_family_transcription_factor

MELO3C034251.2	1.319643901	5.25E-17	up	At2g01340
MELO3C034276.2	-1.184319067	0.003531789	down	katanin_p60_ATPase-containing_subunit_A1_iso
MELO3C034389.2	-1.163082218	0.002168774	down	Thioredoxin
MELO3C034589.2	-1.390319746	1.90E-07	down	Estradiol_17-beta-dehydrogenase_1
MELO3C034637.2	2.638039419	0.004526695	up	Root_meristem_growth_factor_9
MELO3C034859.2	-1.914320788	0.001716494	down	Unknown_protein
MELO3C034954.2	-2.234921044	1.47E-05	down	cellulose_synthase-like_protein_H2
MELO3C035179.2	-5.836110796	0.003724626	down	Fatty_acyl-CoA_reductase
MELO3C035278.2	-6.426768128	1.09E-15	down	abscisic_acid_8'-hydroxylase_2
MELO3C035349.2	-2.582286869	7.29E-27	down	alpha_carbonic_anhydrase_7-like
MELO3C035491.2	3.450804684	0.000504696	up	tetrahydrocannabinolic_acid_synthase-like
MELO3C035526.2	-2.641013489	0.000770875	down	(3S,6E)-nerolidol_synthase_1-like
MELO3C035538.2	-1.536364451	0.001069067	down	cytochrome_P450_71A1-like
MELO3C035583.2	-1.013457832	9.63E-09	down	Unknown_protein
MELO3C035674.2	-1.444657196	9.47E-06	down	Glucan_endo-1,3-beta-glucosidase
MELO3C035679.2	-1.958706758	0.000379502	down	twinkle_homolog_protein,_chloroplastic/mitochondrial
MELO3C035769.2	-1.076790168	0.001216149	down	Unknown_protein

Significantly enriched Go terms

Go Term	pvalue	FDR
Biological Process:oxidation reduction(GO:0055114)	1.30E-14	3.30E-12
Biological Process:cell wall macromolecule metabolic process(GO:0044036)	7.60E-07	8.10E-05
Biological Process:oxylipin biosynthetic process(GO:0031408)	9.70E-07	8.10E-05
Biological Process:oxylipin metabolic process(GO:0031407)	1.40E-06	8.50E-05
Biological Process:hormone metabolic process(GO:0042445)	4.50E-06	0.00021
Biological Process:response to biotic stimulus(GO:0009607)	1.10E-05	0.00021
Biological Process:auxin biosynthetic process(GO:0009851)	1.20E-05	0.00021
Biological Process:regulation of biosynthetic process(GO:0009889)	1.20E-05	0.00021
Biological Process:regulation of transcription, DNA-dependent(GO:0006355)	9.10E-06	0.00021
Biological Process:regulation of RNA metabolic process(GO:0051252)	1.10E-05	0.00021
Biological Process:regulation of cellular biosynthetic process(GO:0031326)	1.20E-05	0.00021
Biological Process:regulation of cellular metabolic process(GO:0031323)	6.70E-06	0.00021
Biological Process:regulation of transcription(GO:0045449)	9.00E-06	0.00021
Biological Process:regulation of macromolecule biosynthetic process(GO:0010556)	1.10E-05	0.00021
Biological Process:regulation of nucleobase, nucleoside, nucleotide and nucleic acid metabolic process(GO:0019219)	1.20E-05	0.00021
Biological Process:regulation of nitrogen compound metabolic process(GO:0051171)	1.30E-05	0.00021
Biological Process:defense response(GO:0006952)	1.70E-05	0.00025

Biological Process:auxin metabolic process(GO:0009850)	2.50E-05	0.00035
Biological Process:regulation of primary metabolic process(GO:0080090)	2.70E-05	0.00036
Biological Process:regulation of metabolic process(GO:0019222)	4.00E-05	0.0005
Biological Process:hormone biosynthetic process(GO:0042446)	6.50E-05	0.00076
Biological Process:regulation of gene expression(GO:0010468)	6.70E-05	0.00076
Biological Process:hemicellulose metabolic process(GO:0010410)	8.20E-05	0.00089
Biological Process:regulation of macromolecule metabolic process(GO:0060255)	9.80E-05	0.00095
Biological Process:xyloglucan metabolic process(GO:0010411)	9.30E-05	0.00095
Biological Process:cell wall polysaccharide metabolic process(GO:0010383)	9.80E-05	0.00095
Biological Process:response to stimulus(GO:0050896)	0.00011	0.001
Biological Process:RNA biosynthetic process(GO:0032774)	0.00014	0.0012
Biological Process:regulation of hormone levels(GO:0010817)	0.00016	0.0014
Biological Process:transcription(GO:0006350)	0.00018	0.0015
Biological Process:transcription, DNA-dependent(GO:0006351)	0.0002	0.0016
Biological Process:response to hydrogen peroxide(GO:0042542)	0.00031	0.0024
Biological Process:systemic acquired resistance(GO:0009627)	0.00032	0.0025
Biological Process:response to stress(GO:0006950)	0.00066	0.0049
Biological Process:cellular glucan metabolic process(GO:0006073)	0.00085	0.0061
Biological Process:hydrogen peroxide catabolic process(GO:0042744)	0.00096	0.0063
Biological Process:cellular response to hydrogen peroxide(GO:0070301)	0.00096	0.0063
Biological Process:cellular cell wall macromolecule metabolic process(GO:0010382)	0.00094	0.0063
Biological Process:response to reactive oxygen species(GO:0000302)	0.001	0.0067
Biological Process:hydrogen peroxide metabolic process(GO:0042743)	0.0011	0.0067
Biological Process:regulation of cellular process(GO:0050794)	0.0013	0.0077
Biological Process:defense response, incompatible interaction(GO:0009814)	0.0014	0.008
Biological Process:response to chemical stimulus(GO:0042221)	0.0014	0.008
Biological Process:carbohydrate metabolic process(GO:0005975)	0.0016	0.009
Biological Process:regulation of biological process(GO:0050789)	0.0019	0.011
Biological Process:auxin homeostasis(GO:0010252)	0.002	0.011
Biological Process:response to inorganic substance(GO:0010035)	0.0028	0.015
Biological Process:glucan metabolic process(GO:0044042)	0.003	0.016
Biological Process:polysaccharide metabolic process(GO:0005976)	0.0031	0.016
Biological Process:cellular response to reactive oxygen species(GO:0034614)	0.0031	0.016
Biological Process:oxygen and reactive oxygen species metabolic process(GO:0006800)	0.0034	0.017
Biological Process:cellular response to oxidative stress(GO:0034599)	0.0041	0.02
Biological Process:fatty acid biosynthetic process(GO:0006633)	0.0052	0.024
Biological Process:biological regulation(GO:0065007)	0.0061	0.028

Biological Process:cell wall organization or biogenesis(GO:0071554)	0.007	0.032
Biological Process:response to other organism(GO:0051707)	0.0076	0.034
Biological Process:metal ion transport(GO:0030001)	0.0082	0.036
Biological Process:multidrug transport(GO:0006855)	0.0087	0.036
Biological Process:response to drug(GO:0042493)	0.0087	0.036
Biological Process:drug transport(GO:0015893)	0.0087	0.036
Biological Process:cellular response to chemical stimulus(GO:0070887)	0.011	0.043
Biological Process:cell wall biogenesis(GO:0042546)	0.011	0.045
Mollecular Function:heme binding(GO:0020037)	3.20E-16	4.60E-14
Mollecular Function:tetrapyrrole binding(GO:0046906)	4.70E-15	3.40E-13
Mollecular Function:iron ion binding(GO:0005506)	1.20E-14	4.50E-13
Mollecular Function:oxidoreductase activity(GO:0016491)	1.10E-14	4.50E-13
Mollecular Function:oxidoreductase activity, acting on paired donors, with incorporation or reduction of molecular	2.90E-13	8.40E-12
Mollecular Function:transcription factor activity(GO:0003700)	1.80E-08	4.30E-07
Mollecular Function:transcription regulator activity(GO:0030528)	4.60E-08	9.50E-07
Mollecular Function:oxidoreductase activity, acting on single donors with incorporation of molecular oxygen, incorporation	5.60E-08	1.00E-06
Mollecular Function:oxidoreductase activity, acting on single donors with incorporation of molecular oxygen(GO:0016701)	2.00E-07	3.30E-06
Mollecular Function:transferase activity, transferring hexosyl groups(GO:0016758)	2.10E-06	3.00E-05
Mollecular Function:dioxygenase activity(GO:0051213)	2.70E-05	0.00036
Mollecular Function:chitin binding(GO:0008061)	4.80E-05	0.00053
Mollecular Function:indole-3-acetic acid amido synthetase activity(GO:0010279)	4.80E-05	0.00053
Mollecular Function:monooxygenase activity(GO:0004497)	5.20E-05	0.00054
Mollecular Function:xyloglucan:xyloglucosyl transferase activity(GO:0016762)	7.20E-05	0.0007
Mollecular Function:transferase activity, transferring glycosyl groups(GO:0016757)	0.00011	0.001
Mollecular Function:carbamoyl-phosphate synthase activity(GO:0004086)	0.00022	0.0017
Mollecular Function:carbamoyl-phosphate synthase (glutamine-hydrolyzing) activity(GO:0004088)	0.00022	0.0017
Mollecular Function:terpene synthase activity(GO:0010333)	0.00028	0.0021
Mollecular Function:carbon-oxygen lyase activity, acting on phosphates(GO:0016838)	0.00048	0.0035
Mollecular Function:polysaccharide binding(GO:0030247)	0.0008	0.0052
Mollecular Function:pattern binding(GO:0001871)	0.0008	0.0052
Mollecular Function:lyase activity(GO:0016829)	0.00083	0.0052
Mollecular Function:hydrolase activity, acting on glycosyl bonds(GO:0016798)	0.00089	0.0054
Mollecular Function:carbon-nitrogen ligase activity, with glutamine as amido-N-donor(GO:0016884)	0.0014	0.008
Mollecular Function:hydrolase activity, hydrolyzing O-glycosyl compounds(GO:0004553)	0.0015	0.0081
Mollecular Function:ion binding(GO:0043167)	0.0015	0.0081
Mollecular Function:metal ion binding(GO:0046872)	0.0017	0.0087
Mollecular Function:cation binding(GO:0043169)	0.0018	0.009

Mollecular Function:oxidoreductase activity, acting on peroxide as acceptor(GO:0016684)	0.0021	0.0097
Mollecular Function:peroxidase activity(GO:0004601)	0.0021	0.0097
Mollecular Function:calcium ion binding(GO:0005509)	0.0024	0.011
Mollecular Function:antioxidant activity(GO:0016209)	0.0045	0.02
Mollecular Function:drug transmembrane transporter activity(GO:0015238)	0.0087	0.037
Cellular Component:extracellular region(GO:0005576)	1.80E-06	9.00E-05
Cellular Component:apoplast(GO:0048046)	0.00061	0.015

Functional categorization

**Gene Function**	**Gene number**	**P value**
**misc**	115	1.33E-13
**stress**	65	9.06E-09
**hormone metabolism**	58	2.57E-08
**photosynthesis**	32	5.63E-08
**secondary metabolism**	44	3.98E-07
**RNA**	141	1.48E-05
**transport**	62	0.000101033
**cell wall**	33	0.001254765
**metal handling**	7	0.02408734
**development**	39	0.02748168
**signalling**	59	0.0311088
**redox**	13	0.04827115
**N-metabolism**	3	0.059715
**major CHO metabolism**	6	0.138438
**S-assimilation**	2	0.1612167
**fermentation**	2	0.1741473
**polyamine metabolism**	2	0.1741473
**tetrapyrrole synthesis**	3	0.21101
**mino acid metabolism**	1	0.2544197
**gluconeogenesis**	1	0.3071757
**C1-metabolism**	2	0.3079939
**minor CHO metabolism**	6	0.3216764
**mitochondrial electron transport**	5	0.4978854
**glycolysis**	3	0.5948012
**OPP**	1	0.6147659
**lipid metabolism**	13	0.6771358
**TCA**	2	0.7226277

**nucleotide metabolism**	4	0.7697857
**Biodegradation of Xenobiotics**	1	0.7777333
**amino acid metabolism**	6	0.9362174
**cell**	15	0.9944961
**DNA**	5	0.9998216
**protein**	60	0.9999993
**not assigned**	350	-

Table S6 The DEGs information, enriched Gene Ontology terms and functional categorization of Common DEGs

Common DEGs with same expression pattern between two parental lines and two bulks.

Gene_name	Status	Function annotation
MELO3C000200.2	down	Bidirectional_sugar_transporter_SWEET
MELO3C000668.2	down	Protein_DETOXIFICATION
MELO3C000922.2	down	NAC_domain-containing_protein
MELO3C001014.2	up	protein_NRT1/_PTR_FAMILY_4.6-like
MELO3C001058.2	down	Lipoxygenase
MELO3C002076.2	up	Transcription_factor_TEOSINTE_BRANCHED_1
MELO3C002191.2	down	Short-chain_dehydrogenase/reductase
MELO3C002220.2	up	AAA-ATPase_At3g28580-like
MELO3C002221.2	up	AAA-ATPase_At3g28580-like
MELO3C002286.2	up	Receptor_protein_kinase
MELO3C002310.2	down	cytochrome_P450_71A1-like
MELO3C002420.2	up	Retrovirus-related_Pol_polyprotein_from_transposon_TNT_1-94
MELO3C002460.2	up	At3g20340
MELO3C002514.2	up	BTB/POZ_domain-containing_protein_At3g22104
MELO3C002875.2	up	WRKY_family_transcription_factor
MELO3C003164.2	down	Cysteine/Histidine-rich_C1_domain_family_protein
MELO3C003188.2	down	glutathione_S-transferase_U8-like
MELO3C003195.2	down	17.5_kDa_class_I_heat_shock_protein
MELO3C003275.2	up	Peroxidase
MELO3C003817.2	up	DNA_polymerase_epsilon_catalytic_subunit_A
MELO3C003823.2	down	jacalin-related_lectin_3-like
MELO3C004003.2	down	La-related_protein_6_isoform_1
MELO3C004181.2	up	homeobox_protein_knotted-1-like_1_isoform_X1
MELO3C004242.2	up	Lipoxygenase
MELO3C004244.2	up	Lipoxygenase
MELO3C004245.2	down	Lipoxygenase
MELO3C004250.2	up	Lipoxygenase
MELO3C004556.2	down	heat_stress_transcription_factor_C-1-like
MELO3C004753.2	up	subtilisin-like_protease_SBT3.18
MELO3C004801.2	down	cytochrome_P450_71A1-like
MELO3C004914.2	up	Cysteine_proteinase_inhibitor
MELO3C005319.2	up	Calcium-binding_protein
MELO3C005476.2	up	Auxin-responsive_protein
MELO3C005611.2	down	transcription_factor_bHLH120-like
MELO3C005736.2	down	Cytoplasmic_tRNA_2-thiolation_protein
MELO3C005923.2	down	Chloroplast_small_heat_shock_protein
MELO3C005947.2	up	basic_blue_protein
MELO3C006353.2	down	Glutathione_S-transferase
MELO3C006365.2	down	FBT8
MELO3C006431.2	up	ethylene-responsive_transcription_factor_ERF098-like
MELO3C006501.2	down	heat_stress_transcription_factor_A-6b
MELO3C006546.2	up	BRI1_kinase_inhibitor_1-like
MELO3C006552.2	down	Glucose-1-phosphate_adenylyltransferase
MELO3C006773.2	up	Rapid_ALkalinization_Factor
MELO3C006944.2	down	ferric_reduction_oxidase_4-like
MELO3C007255.2	down	NAC_domain-containing_protein
MELO3C007279.2	down	Pollen_Ole_e_1_allergen_and_extensin_family_protein
MELO3C007315.2	down	Internal_alternative_NAD(P)H-ubiquinone_oxidoreductase_A1
MELO3C007337.2	up	Zinc_finger
MELO3C007391.2	up	Glycosyl_transferase
MELO3C007470.2	up	WRKY_family_transcription_factor
MELO3C007482.2	down	Cytochrome_P450_family_protein
MELO3C007630.2	up	protein_SHORT-ROOT
MELO3C007663.2	down	transcription_factor_RAX2
MELO3C007687.2	up	Phosphoenolpyruvate_carboxykinase
MELO3C007799.2	down	cytochrome_P450_CYP736A12-like
MELO3C007877.2	up	Protein_SHI_RELATED_SEQUENCE_1
MELO3C007949.2	down	thiosulfate_sulfurtransferase_16
MELO3C008203.2	up	protein_LYK2
MELO3C009141.2	down	PEBP_(Phosphatidylethanolamine-binding_protein)_family_protein
MELO3C009389.2	down	Glycosyltransferase
MELO3C009390.2	down	Glycosyltransferase
MELO3C009391.2	down	Glycosyltransferase
MELO3C009441.2	up	ethylene-responsive_transcription_factor_ERF024-like
MELO3C009674.2	down	Beta-glucosidase

MELO3C009681.2	up	At3g57450
MELO3C009686.2	up	Pleiotropic_drug_resistance_ABC_transporter
MELO3C010249.2	up	chitotriosidase-1-like
MELO3C010312.2	down	36.4_kDa_proline-rich_protein
MELO3C010318.2	down	Clathrin_assembly_protein
MELO3C010504.2	down	At1g77400
MELO3C010719.2	down	vinorine_synthase
MELO3C010984.2	up	Protein_SHI_RELATED_SEQUENCE_1
MELO3C011252.2	up	EG45-like_domain_containing_protein
MELO3C011270.2	up	Adenine_phosphoribosyltransferase
MELO3C011443.2	down	Glycosyltransferase
MELO3C011474.2	up	Ankyrin_repeat_family_protein
MELO3C011475.2	up	Ankyrin_repeat_family_protein
MELO3C011476.2	up	Ankyrin_repeat_family_protein
MELO3C011928.2	down	cytochrome_P450_71B19-like
MELO3C011971.2	up	Heme-binding_protein_2
MELO3C011991.2	down	Hexosyltransferase
MELO3C012015.2	down	WAT1-related_protein
MELO3C012016.2	up	MLP-like_protein_423
MELO3C012055.2	down	NRT1/PTR_family_protein_2.2
MELO3C012065.2	up	protein_NRT1/_PTR_FAMILY_4.6
MELO3C012127.2	up	protein_argonaute_7
MELO3C012162.2	up	BnaC07g22510D_protein
MELO3C012263.2	down	zinc_finger_protein_6
MELO3C012352.2	down	germin-like_protein_subfamily_3_member_2
MELO3C012550.2	down	polyol_transporter_5-like
MELO3C012626.2	down	Unknown_protein
MELO3C012712.2	down	NAD(P)H-quinone_oxidoreductase_subunit_M
MELO3C012987.2	down	Avr9/Cf-9_rapidly_elicited_protein
MELO3C013003.2	up	Auxin-responsive_protein
MELO3C013014.2	down	Inositol_oxygenase
MELO3C013159.2	up	casein_kinase_II_subunit_beta-2
MELO3C013246.2	up	Glutamate_receptor
MELO3C013562.2	down	Flavin-containing_monooxygenase
MELO3C013868.2	down	Cytochrome_P450_family_ent-
MELO3C013956.2	down	14_kDa_proline-rich_protein_DC2.15
MELO3C013960.2	up	protein_NRT1/_PTR_FAMILY_6.3-like
MELO3C014047.2	down	(-)-germacrene_D_synthase-like
MELO3C014257.2	up	Arabidopsis_thaliana_genomic_DNA
MELO3C014360.2	up	Glycosyltransferase
MELO3C014367.2	down	ABC1_family_protein
MELO3C014465.2	up	Xyloglucan_endotransglucosylase/hydrolase
MELO3C014505.2	up	NAC_domain-containing_protein_90
MELO3C014516.2	down	NDR1/HIN1-like_protein_12
MELO3C014568.2	down	Alanine:glyoxylate_aminotransferase
MELO3C014803.2	down	Phosphate_carrier
MELO3C014818.2	down	Cystinosin-like_protein
MELO3C014827.2	down	Class_I_heat_shock_protein
MELO3C015118.2	down	Unknown_protein
MELO3C015183.2	up	phospholipase_A1-Igamma3
MELO3C015221.2	down	cyanogenic_beta-glucosidase-like
MELO3C015388.2	down	cytochrome_P450_CYP72A219-like
MELO3C015414.2	up	receptor-like_protein_kinase_HSL1
MELO3C015852.2	up	adenylate_isopentenyltransferase_3
MELO3C015943.2	up	zinc_finger_protein_6-like
MELO3C016167.2	down	Glutathione_S-transferase
MELO3C016224.2	down	9-cis-epoxycarotenoid_dioxygenase
MELO3C016359.2	up	Protein_SPIRAL1
MELO3C016444.2	down	NAC_domain-containing_protein_55
MELO3C016588.2	down	(+)-gamma-cadinene_synthase
MELO3C016616.2	up	Indole-3-acetic_acid-amido_synthetase_GH3.3
MELO3C016932.2	down	Eukaryotic_initiation_factor_4F_subunit_p150_isofor
MELO3C016976.2	down	zinc_finger_protein_1-like
MELO3C016980.2	down	ethylene-responsive_transcription_factor_ERF003-like
MELO3C017013.2	up	DNA-directed_RNA_polymerase_subunit_beta
MELO3C017582.2	down	Protein_MARD1
MELO3C017831.2	up	aquaporin_NIP6-1
MELO3C017917.2	down	Calvin_cycle_protein_CP12
MELO3C018065.2	down	BnaA07g05330D_protein
MELO3C018166.2	up	Indole-3-acetic_acid-amido_synthetase_GH3.3
MELO3C018316.2	up	subtilisin-like_protease_SBT1.1
MELO3C018485.2	down	22.0_kDa_class_IV_heat_shock_protein

MELO3C018528.2	down	Photosystem_I_reaction_center_subunit_N
MELO3C018600.2	down	IgA_FC_receptor
MELO3C018733.2	up	MLP_protein
MELO3C018799.2	up	Cysteine-rich_receptor-like_kinase
MELO3C018814.2	down	Protein_DETOXIFICATION
MELO3C018886.2	up	BnaA09g54870D_protein
MELO3C019099.2	down	CDPK-related_kinase_1
MELO3C019337.2	down	Two-component_response_regulator-like_APRR2
MELO3C019552.2	down	Neuronal_PAS_domain_protein
MELO3C019835.2	down	Protein_nuclear_fusion_defective_4
MELO3C020264.2	down	kirola-like
MELO3C020268.2	down	MLP-like_protein_28
MELO3C020357.2	down	Sucrose-phosphate_synthase
MELO3C020559.2	down	GDP-mannose_transporter
MELO3C020975.2	up	cytochrome_P450_78A7
MELO3C021085.2	down	Membrane_protein_of_er_body-like_protein
MELO3C021151.2	up	PLATZ_transcription_factor_family_protein
MELO3C021249.2	down	Hexosyltransferase
MELO3C021426.2	up	Protein_TERMINAL_FLOWER_1
MELO3C021578.2	up	LOB_domain-containing_protein_25
MELO3C021607.2	down	pectate_lyase-like
MELO3C021811.2	down	Ribose-5-phosphate_isomerase_A
MELO3C021886.2	down	S-norcoclaurine_synthase_1-like
MELO3C021901.2	down	Alpha/beta_hydrolase-3
MELO3C021982.2	up	ABC_transporter_B_family_protein
MELO3C021999.2	up	Expansin_protein
MELO3C022007.2	down	transmembrane_emp24_domain-containing_protein_p24delta9
MELO3C022028.2	down	Transcription_factor_bHLH151
MELO3C022207.2	down	Retrovirus-related_Pol_polyprotein_from_transposon_TNT_1-94
MELO3C022234.2	up	cyclin-U2-1
MELO3C022341.2	down	Bidirectional_sugar_transporter_SWEET
MELO3C022372.2	down	Cytochrome_P450
MELO3C022376.2	down	Cytochrome_P450
MELO3C022377.2	down	Cytochrome_P450
MELO3C022429.2	down	Amaranthin-like_lectin
MELO3C022430.2	down	Amaranthin-like_lectin
MELO3C022542.2	down	MYB_transcription_factor-like
MELO3C022791.2	down	Protein_MARD1
MELO3C022804.2	down	heavy_metal-
MELO3C023161.2	down	nuclear_transcription_factor_Y_subunit_A-7-like
MELO3C023360.2	down	Thionin-like_protein_2
MELO3C023391.2	up	Plant_Tudor-like_protein
MELO3C023498.2	up	Protein_UPSTREAM_OF_FLC
MELO3C023879.2	down	Ribulose_bisphosphate_carboxylase/oxygenase_activase_family_protein
MELO3C024014.2	up	Carboxypeptidase
MELO3C024163.2	up	acanthoscurrin-1-like
MELO3C024232.2	up	Transcription_repressor_OFP14
MELO3C024263.2	up	aquaporin_TIP2-1
MELO3C024425.2	up	Receptor-like_protein_kinase
MELO3C024545.2	down	Transmembrane_9_superfamily_member
MELO3C024841.2	down	proline-rich_receptor-like_protein_kinase_PERK2
MELO3C025761.2	up	MLO-like_protein
MELO3C025814.2	up	Ankyrin_repeat-containing_protein
MELO3C025855.2	down	Alkyl_transferase
MELO3C026001.2	down	Late_embryogenesis_abundant_protein
MELO3C026269.2	up	LOB_domain-containing_protein_25
MELO3C026299.2	up	MADS-box_transcription_factor
MELO3C026488.2	down	cytochrome_P450_82A3-like
MELO3C026492.2	down	Cytochrome_P450
MELO3C026558.2	down	Glutamate_receptor
MELO3C026677.2	up	Receptor_protein_kinase
MELO3C026784.2	down	serine/threonine-protein_kinase_BLUS1-like
MELO3C026808.2	up	SABATH_methyltransferase_9
MELO3C027015.2	down	ACT_domain-containing_protein
MELO3C027124.2	down	17.5_kDa_class_I_heat_shock_protein
MELO3C027216.2	up	Transcription_factor
MELO3C027727.2	down	(-)-germacrene_D_synthase-like
MELO3C028593.2	down	Protein_DETOXIFICATION
MELO3C029682.2	down	Transcriptional_corepressor_LEUNIG

MELO3C029738.2	down	Terpene_cyclase/mutase_family_member
MELO3C030287.2	up	WRKY_transcription_factor
MELO3C030721.2	down	Beta-glucosidase
MELO3C030722.2	down	Beta-glucosidase
MELO3C030868.2	down	GDSL_esterase/lipase_1-like
MELO3C031037.2	up	Unknown_protein
MELO3C031144.2	down	Unknown_protein
MELO3C031342.2	up	UDP-sugar_transporter-like_protein
MELO3C031450.2	down	GDSL_esterase/lipase_At5g22810
MELO3C031571.2	up	UDP-sugar_transporter_sqv-7-like_isoform_X2
MELO3C031621.2	down	Wall-associated_receptor_kinase-like_20
MELO3C031780.2	up	Unknown_protein
MELO3C031893.2	up	protein_disulfide_isomerase-like_1-6
MELO3C032143.2	down	Unknown_protein
MELO3C032879.2	down	Unknown_protein
MELO3C032937.2	down	glucomannan_4-beta-mannosyltransferase_9-like
MELO3C033082.2	up	Unknown_protein
MELO3C033198.2	down	Glutathione_S-transferase
MELO3C033753.2	down	Purple_acid_phosphatase
MELO3C034389.2	down	Thioredoxin
MELO3C035349.2	down	alpha_carbonic_anhydrase_7-like
MELO3C035583.2	down	Unknown_protein

Significantly enriched Go terms

**Go Term**	**P value**	**FDR**
Biological Process:A236:D261oxylipin biosynthetic process(GO:0031408)	1.00E-05	0.00053
Biological Process:oxylipin metabolic process(GO:0031407)	1.20E-05	0.00053
Biological Process:oxidation reduction(GO:0055114)	0.0043	0.028
Biological Process:response to biotic stimulus(GO:0009607)	0.0048	0.028
Biological Process:regulation of biosynthetic process(GO:0009889)	0.0043	0.028
Biological Process:regulation of transcription, DNA-dependent(GO:0006355)	0.0019	0.028
Biological Process:regulation of macromolecule biosynthetic process(GO:0010556)	0.0042	0.028

Biological Process:fatty acid biosynthetic process(GO:0006633)	0.0021	0.028
Biological Process:fatty acid metabolic process(GO:0006631)	0.0038	0.028
Biological Process:RNA biosynthetic process(GO:0032774)	0.0044	0.028
Biological Process:regulation of RNA metabolic process(GO:0051252)	0.002	0.028
Biological Process:regulation of cellular biosynthetic process(GO:0031326)	0.0043	0.028
Biological Process:regulation of cellular metabolic process(GO:0031323)	0.005	0.028
Biological Process:regulation of transcription(GO:0045449)	0.0022	0.028
Biological Process:regulation of nucleobase, nucleoside, nucleotide and nucleic acid metabolic process(GO:0019219)	0.0029	0.028
Biological Process:regulation of nitrogen compound metabolic process(GO:0051171)	0.0029	0.028
Biological Process:regulation of primary metabolic process(GO:0080090)	0.0063	0.03
Biological Process:transcription, DNA-dependent(GO:0006351)	0.0061	0.03

Biological Process:regulation of gene expression(GO:0010468)	0.0064	0.03
Biological Process:transcription(GO:0006350)	0.0067	0.03
Biological Process:regulation of metabolic process(GO:0019222)	0.0093	0.037
Biological Process:regulation of macromolecule metabolic process(GO:0060255)	0.0092	0.037
Biological Process:monocarboxylic acid metabolic process(GO:0032787)	0.0095	0.037
Biological Process:signaling pathway(GO:0023033)	0.01	0.037
Mollecular Function:oxidoreductase activity, acting on single donors with incorporation of molecular oxygen(GO:0016701)	5.70E-06	0.00025
Mollecular Function:oxidoreductase activity, acting on paired donors, with incorporation or reduction of molecular oxygen(GO:0016705)	2.10E-05	0.00031
Mollecular Function:oxidoreductase activity, acting on single donors with incorporation of molecular oxygen, incorporation of two atoms of oxygen(GO:0016702)	1.80E-05	0.00031
Mollecular Function:heme binding(GO:0020037)	6.00E-05	0.00067

Mollecular Function:iron ion binding(GO:0005506)	0.00011	0.00096
Mollecular Function:tetrapyrrole binding(GO:0046906)	0.00023	0.0017
Mollecular Function:dioxygenase activity(GO:0051213)	0.00045	0.0029
Mollecular Function:monooxygenase activity(GO:0004497)	0.0018	0.0099
Mollecular Function:transferase activity, transferring hexosyl groups(GO:0016758)	0.0034	0.015
Mollecular Function:oxidoreductase activity(GO:0016491)	0.003	0.015
Mollecular Function:transferase activity, transferring glycosyl groups(GO:0016757)	0.0058	0.024
Mollecular Function:transcription regulator activity(GO:0030528)	0.011	0.042

Functional categorization

**Gene Function**	**Gene number**	**pvalue**
misc	26	3.09E-05
stress	17	0.000101546
hormone metabolism	14	0.000659284
development	14	0.001859251
transport	16	0.003396382
photosynthesis	5	0.04986368
minor CHO metabolism	3	0.06614363
gluconeogenesis	1	0.07076667
secondary metabolism	7	0.07094787
metal handling	2	0.1025893
RNA	24	0.1374383
major CHO metabolism	2	0.1487036
tetrapyrrole synthesis	1	0.2651602
signalling	11	0.2825896
TCA	1	0.3970676
cell wall	4	0.4699221
mitochondrial electron transport	1	0.5997861
nucleotide metabolism	1	0.6465512

redox	1	0.7719168
lipid metabolism	2	0.7732942
amino acid metabolism	1	0.8640269
cell	1	0.9947355
protein	7	0.9998278
not assigned	66	-

Table S7. Primers used in this study

Marker Name	Forward primer	Reverse primer	Comment
CmSSR17093	TTTTTGCCCTTCTGGATGTC	GATCTTGATAGCCCCAACGA	used for mapping
CmSSR17144	CCAATGCCTAACCTAACCGA	GAGAATGGAAGGAAAAGGGC	used for mapping
CmSSR17145	TTCTCATTTTGGCCCTTACAA	ACGGTTGCACATGAAATGAA	used for mapping
CmSSR17294	ACATGGCGTAGTTAGGGCAG	GTTAACCCTCCTCACCTCCC	used for mapping
CmSSR17293	TTCTTCTCTCATTTACCTCAACCA	TGTTTGAACCAAATAGCTCTCAG	used for mapping
CmSSR17424	CCATTTGCCCTTGTGAAGTT	CACAGAATTCTTTCCCCACC	used for mapping
CmSSR17626	TGCATGGTCCTGTGCATATC	AACCGCTATTTTGCAATGGA	used for mapping
CmSSR01501	GAAAAATGGTTGTTTGGGGA	GACTTGATTTCTTGAAGAGGGAAA	used for mapping
CmSSR17701	CCACATGGGGTGGTTCTTTA	CCCTATCATCACAAAATTAAGGG	used for mapping
CmSSR17736	AAAGGACTGCCTTTTGGGTT	CGAAAGCTAAATAGAGATGAATTGG	used for mapping
CmSSR18435	AACACCTTTGGTTTGTCAAATG	TTGACCTAACACGTACGGCA	used for mapping
CmSSR18451	TTTTGTGAACTCGAGAAAAGG	TCCCCAACTTTCTTCTTTCTTT	used for mapping
CmSSR18529	TGGGGAATTGGAAAAAGATG	TGAAAGGAAGAATGAGGGGA	used for mapping
CmSSR18542	TGGAAAAGAGTGGGTTTTGG	CTTGGAAGCAAAGTGAAGGG	used for mapping
CmSSR18547	CTTTGATGGTAAGCTTCAGTGC	CGTCCAATCATCCTTTTGTTG	used for mapping
CmSSR18578	CTTACGCTCAAAATCCCCAA	ACCCTTCCTCATTGTGTTCG	used for mapping
CmSSR18632	TGTCTCGTCCTTTCATGGTG	GTTTTGTTTTGGCCGTCTGT	used for mapping
CmSSR18656	TAGAGGACACCCACACCACA	CCAACTCCCAATTTCCAAGA	used for mapping
CmSSR18669	CTCTTTGGGCTTCAACTTCG	GGTCCCAAATTCTGATTGGA	used for mapping
CmSSR18726	TTCCAAATGCAATGTCAGGA	TGTTGCCCCATACCCATATT	used for mapping
CmSSR18969	GTCTTTGATCGCAAGCCACT	CCTACCACACACACCACTCG	used for mapping
CmSSR18972	AACCGCAAATACGAGACCTG	TCTCCTGCATAAACCCCAAG	used for mapping
CmSSR19068	ACAAGCTGAGGAATTGGGTG	TTCAACTTCCAATTTTGCCC	used for mapping
CmSSR19083	TTGAATCTAGGGAGGCTGGA	ACATGGGGCCTTCTTCTCTT	used for mapping
Indel1	TTAGGTAAGGCAAGCCGGTA	GCAAAGCATGTTTTCACTTTGA	used for mapping
Indel3	CACAGATTCATTTCGCCATC	TCGATTCGTGTTATTTGGTCA	used for mapping
Indel4	CACAACCATTCAAGTTAAGTTTCG	TGGGATTAAGGGAATGTTTCA	used for mapping
Indel8	TGATGTCCTGTCAACAAACCA	ATGTCACATTACCATAATTCAAACTT	used for mapping
Indel9	GCGCGTCTGAATTTAAACCT	GGAAGCATTCCAAATTCCAA	used for mapping
Indel12	TTATTGTTTCCTTTTTATCTTTTTCC	CTCGCACGTGTGTCATTCTT	used for mapping
Indel14	GCATTTAATTTGAAGTTATGGA	TTCACCCATTTCATTACAAAA	used for mapping
dCAPS1	TGTGCCTGGTGAAAACTCAG	TGTAGACTGTGCTTGAAGCTCG	used for mapping
dCAPS2	TCATCCAAAGTACTTCACATAGGG	GTTCTTGCATTAAATTTAACGGAGA	used for mapping
dCAPS4	TACCTGTGGGGACCAACACT	AAGATTTAAAAAATACATCTAC	used for mapping
CmSI-clone	ATGAAGTATGTGAAGAGGGCA	TTACTCACTGTTTTGTGATATTACC	used for cloning
CmSI-promoter-clone	GAAATGAAAAATGAAAAAAAAATGA	TTTACTCAATTTTTGCAGTGTTCAT	used for cloning
q-CmSI	GAGACCGAACTGGAAACCGT	GATGATCCCAGAGACTGCCG	Primers for qRT-
ACTIN	ATTCTTGCATCTCTAAGTACCTTCC	CCAACTAAAGGGAAATAACTCACC	Primers for qRT-
CmSI-in situ	GATTTAGGTGACACTATAGaatGCTTTCCACCTGTAATTGGTCTG	tgTAATACGACTCACTATAGGGCTAAGATTGTCAGGGATTGG	Primers for in situ probes
ProCmSI-1391	ctaagaggagtccaccatggGAAATGAAAAATGAAAAAAAAATGA	TCTACAGGACGTAAACTAGTTTTACTCAATTTTTGCAGTGTTCAT	Primers for GUS construct
O-CmSI	AGCTTCTGCAGGGGCCCGGGATGAAGTATGTGAAGAGGGCA	ATGGTACCGGATCCACTAGTCTCACTGTTTTGTGATATTACC	Primers for CmSI overexpression construct
O-CmSI-identification	CGGACGCAGAAGGCAATG	TGATGTGGAACGGGAAAA	Primers for PCR identification
CmSI-topmark-SUC	GGAATTCCATATGATGGGAAGGTCTCCTTACTGC	CGCGGATCCATTTCTGATCTCCTAGTTGTCGAAA	Primers for yeast two-hybrid construct
CmSI-406-SUC	GGAATTCCATATGATGGGAAGGTCTCCTTACTG	CGCGGATCCGACTAATGATCTTAAAGTTAAATTTTC	Primers for yeast two-hybrid construct
CmSI-*kinase-SUC*	GGAATTCCATATGATGGGAAGGTCTCCTTACTGCG	CGCGGATCCACTATCAAACATATATGATTTTTGTTTA	Primers for yeast two-hybrid construct
CmPIN2-pPR3-N	ATTAACAAGGCCATTACGGCCATGGCCGGAAGTATAGGAAGAGAC	AACTGATTGGCCGAGGCGGCCTTAGAAGTAATCAAAATGGTGATTAGGGA	Primers for yeast two-hybrid construct
